# Is schizotypic maternal personality linked to sensory gating abilities during infancy?

**DOI:** 10.1007/s00221-019-05554-7

**Published:** 2019-05-13

**Authors:** Eleanor. S. Smith, Trevor J. Crawford, Megan Thomas, Vincent M. Reid

**Affiliations:** 1 Department of Psychology, Lancaster University, Lancaster, LA1 4YF UK; 2 Blackpool Teaching Hospitals NHS Foundation Trust, Blackpool, FY3 8NR UK; 3 Department of Psychology, University of Cambridge, Downing Street, Cambridge, CB2 3EB UK

**Keywords:** EEG, Event-related potential, Sensory gating, Schizotypy, Infancy

## Abstract

Schizotypy is a personality dimension within the general population elevated among schizophrenia-spectrum patients and their first-degree relatives. Sensory gating is the pre-attentional habituation of responses distinguishing between important and irrelevant information. This is measured by event-related potentials, which have been found to display abnormalities in schizophrenic disorders. The current study investigated whether 6-month-old infants of mothers with schizotypic traits display sensory gating abnormalities. The paired-tone paradigm: two identical auditory tones (stimulus 1 and stimulus 2) played 500 ms apart, was used to probe the selective activation of the brain during 15-minutes of sleep. Their mothers completed the Oxford and Liverpool Inventory of Feelings and Experiences-Short Form as an index of schizotypy dimensionality, categorized into: infants of control, and infants of schizotypic, mothers. The findings revealed that although the infants’ P50 components displayed significant differences between stimulus 1 and stimulus 2 in the paired-tone paradigm, there was no clear difference between infants of schizotypic and infants of control mothers. In contrast, all mothers displayed significant differences between stimulus 1 and stimulus 2, as observed in the infants, but also significant differences between their sensory gating ability correlated with schizotypy dimensionality. These findings are consistent with sensory processes, such as sensory gating, evidencing impairment in schizophrenia-spectrum disorders. The present research supports the idea that first-degree relatives of individuals who identify on this spectrum, within the sub-clinical category, do not display the same deficit at 6 postnatal months of age.

**Electronic supplementary material:**

The online version of this article (10.1007/s00221-019-05554-7) contains supplementary material, which is available to authorized users.

## Introduction

The influence of maternal personality on childhood risk factors for mental health is widely acknowledged with links identified between specific parental psychopathology and event-related potential (ERP) components. Core neuropsychological dysfunctions of potential future psychopathologies may be present during childhood, which shape the development of the adult personality (Corr 2010). It is consequently of fundamental interest to determine whether maternal personality influences development during infancy.

Atypical P50 sensory gating is a highly established biological trait of schizophrenia (Raine 2006), observed in individuals with schizotypal personality disorder (Cadenhead et al. 2000) and infants and children of parents with psychoses, or severe anxiety disorders (Ross and Freedman 2015). This work supports its potential as a biomarker for the general risk of psychopathology that potentially extends into infancy (Freedman et al. 2002). However, whether, and to what extent, these dimensions of schizotypy are related to the risk of developing psychosis is still unresolved (Debbané and Barrantes-Vidal 2015). Schizotypal expression during adolescence and adulthood is critically linked to childhood risk markers and endophenotypes, which confer a role of potential developmental facilitators on the road to psychosis proneness (Debbané 2015, p. 88). A developmental model of schizotypy, addressing the progression of traits throughout childhood and adolescence, could hold the necessary ingredients to account for the progressive development of psychotic disorders, such as schizophrenia, throughout these key periods of development, which is a component of the literature that remains to be further understood. Atypical sensory gating is an endophenotype of the schizophrenia-spectrum, but is also an electrophysiological marker that can be identified during infancy. Although it is not clear what the specific effects atypical sensory gating may have on infants’ behaviour, the extent to which they display atypicalities in this ability may provide an indication of whether developmental endophenotypes could be identified as early as 6 months old.

The P50 ERP is strongly associated with sensory gating: the pre-attentional habituation of responses distinguishing between important and irrelevant information (Hall et al. 2011), a largely automatic process and an involuntary step in attentional mechanisms (Lijffijt et al. 2009). Sensory gating is generally observed using the paired-tone paradigm: two identical auditory tones [stimulus 1 (S1) and stimulus 2 (S2)] are played 500 ms apart, whereby participants hear a pair of single-sound stimuli within 50-milliseconds (ms) of each other. Both tones have the same intensity, frequency and pitch, with sensory gating efficacy measured using a ratio of the ERP amplitudes (S2/S1), or by the difference between the mean amplitudes (S1–S2). A low ratio or large difference represents better sensory gating abilities (Freedman et al. 1983; Olincy et al. 2010).

The notion that personality traits and clinical diagnoses lie on the same continuum is not new (Eysenck 1992; Corr 2000) and has stimulated research aimed at identifying core deficits shared by sub-clinical personality traits and clinical psychosis. Schizotypy describes a dynamic continuum of symptomatology, impairments and personality traits (Kwapil and Barrantes-Vidal 2012) that are cognitive, emotional and behavioural, and grouped into a multidimensional structure (i.e. positive, negative, and disorganised) similar to that in schizophrenia (Fonseca-Pedrero et al. 2010). Schizotypy is thought to mimic the subclinical expression of schizophrenia distributed along a continuum, rather than discrete categories (Claridge 1997), illustrating how vulnerability to mental illness can be expressed as a multidimensional personality organisation (Barrantes-Vidal et al. 2015). Schizotypy traits are elevated in children at-risk for the development of schizophrenia during infancy, 2, 10, and 15 years of age (Carlson and Fish 2005), and is therefore, considered to be a sensitive predictor for the later development of schizophrenia-spectrum disorders (Tyrka et al. 1995). As it is not possible to reliably diagnose psychiatric disorders in infants, risk status is generally inferred from parental psychopathology (Keshavan et al. 2008).

Atypical sensory gating shows potential as a candidate endophenotype because the same deficit is observed in non-affected first-degree relatives of schizophrenic patients (Waldo et al. 2000), individuals at-risk of development (Cadenhead et al. 2005), and in schizophrenia-spectrum disorders (Raine 2006; Cadenhead et al. 2000). Importantly, from a developmental standpoint, schizotypy has been associated with endophenotypes and biomarkers whose dimensions can already be assessed during infancy.

The primary aim of the present study was to measure the electrical brain activity of 6-month-old infants (experiment 1) and their mothers (experiment 2) in auditory-gating tasks. Prior research suggests a development trajectory of sensory gating capacities, although the details of these abilities are not clear at 6 months. We, therefore, set out to explore whether measurable changes in sensory gating functions in the offspring of mothers with schizotypic traits could be detected. We hypothesised that abnormalities previously observed in individuals diagnosed with schizophrenia may be present to some extent in those with sub-clinical schizotypy. It was also hypothesised that the infants of mothers displaying schizotypic traits would also exhibit these atypicalities; similarly to the manner in which first-degree relatives of those diagnosed with schizophrenia display sensory gating abnormalities. Specifically, we evaluated whether the 6-month-old infants of schizotypic mothers display smaller differences and larger suppression ratios in the P50 component when explored using the paired-tone paradigm.

## Methods and materials

### Experiment 1: infant cohort

#### Participants

One-hundred and one infants, aged 6 months (*M *= 5.8 months; SD = 9.23 days; 54 male) participated in the study. A 6-month-old infant population was chosen for the present research due to the developmental trajectories observed in the existing sensory gating literature. We know from the literature that sensory gating can be observed from as young as 2 (Hutchison et al. 2017) or 3 months of age (Hunter et al. 2015), although there are inconsistencies in the developmental trajectory due to large age-gaps in the published literature. Sixty-six infants were excluded from the final sample due to: no auditory data collected as the infant did not sleep (*n* = 24), technical issues (*n* = 4), the data not reaching the inclusion criteria: 20% good trials (range of 57–141 paired-stimuli repetitions, dependent on length of sleep period) for each tone (*n* = 27), and the Oxford–Liverpool Inventory of Feelings and Experiences-Short Form (sO-LIFE) scores not identifying with one of the two groups (*n* = 10). Thirty-five infants with a mean age of 5.88 months (SD= 8.57 days; 18 male) were included in the final analysis. The final sample included 14 participants who identified as being an infant of a schizotypic mother (iSZT) and the remaining 21 participants were infants of control mothers (iCON). For one EEG experiment with infants, this is a typical sample size for similar studies (e.g., Stets et al. 2012) or substantially greater than the sample size for studies on schizotypy during development (Hunter et al. 2015). Recruitment was carried out using the Lancaster University Psychology Department of Infant and Child Development infant database. Ethical approval was obtained with the Lancaster University Faculty of Science and Technology Ethics Committee (“Understanding Sensory Processing in Early Development”), and the North West-Lancaster Research Ethics Committee for the NHS.

#### Materials and stimuli

**Fig. 1:**
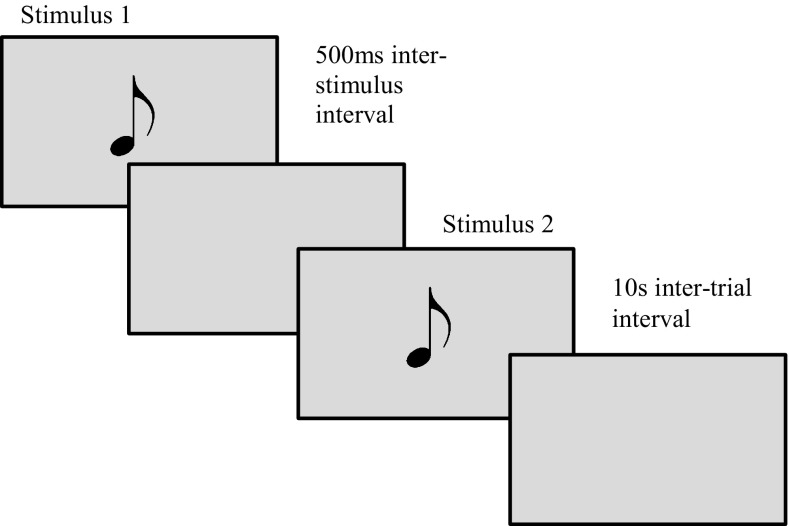
A graphical representation of the paired-tone paradigm. The tones were presented between 70 and 77 dB with a tonal quality of 1000 Hz

The participant experienced a pair of single-sound stimuli, each presented for 500 ms, that was based on Park et al. (2015). See Fig. 1 for a more detailed representation of the paired-tone paradigm. A 500 ms inter-tone interval was present between two identical tones and with a 10 s inter-trial interval, repeated continuously for 15-min or until the infant woke. The paired tones were presented between 70 and 77 dB and had a tonal quality of 1000 Hz. All electrophysiological signals were recorded using Electrical Geodesics Inc. amplifiers (input impedance = 80 kΩ; sampling rate = 500 Hz) and ERPs were measured using an EGI Hydrocel GSN-128 electrode 1.0 net and analysed using Netstation 4.5.4.

#### EEG recording and analysis

The online reference was located at the vertex and during data processing was re-referenced to the average reference. The baseline used for the baseline correction was 200 ms. EEG recordings were condensed to create epochs from 200 ms before to 1000 ms after stimulus-onset. For the elimination of electrical artefacts caused by eye and body movements, EEG data was rejected offline by the visual editing of trial by trial data. This was carried out in conjunction with an artefact detection toolset in Netstation, which highlighted whether a channel was ‘bad’ for more than 80% of the recording, which was determined by a threshold of 200 μV to remove outlier values resulting from artefacts, or if they contained more than 12 bad channels in a trial. Participants required a minimum of 20% good trials for each stimuli to be included in further analyses. Infants experienced a range of 57–141 paired-stimuli repetitions: equating to a minimum of 11–28 good trials per participant, dependent on the length of sleep period, and contributed an average of 44.02 (SD= 21.36; range = 28–105) artefact-free trials for S1, and on average 41.47 (SD= 24.25; range = 25–112) artefact-free trials for S2. A paired-samples *t* test illustrated no significant differences between the number of trials included for S1 and S2 (*t*(35) = 1.839, *p* = 0.074). Following averaging, data were re-referenced to the average reference, by averaging all included channels together, and high-pass filtered at 0.3 Hz, and low-pass filtered at 30 Hz. All infant ERPs computed a mean amplitude and maximum amplitude measure. Differences (S1–S2) and suppression ratios (S2/S1) were calculated and used for further analysis. All analyses were conducted blind to the participant group status.

### P50: stimulus 1

**Fig. 2:**
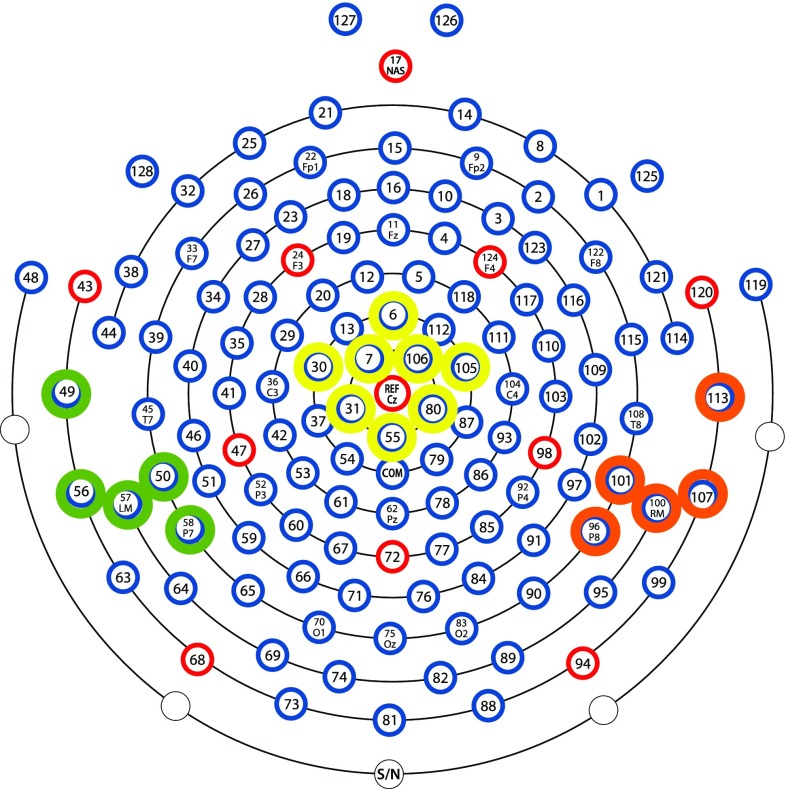
The P50 electrode groupings for the infant cohort: central (6, 7, 30, 31, 55, 80, 105, 106), left-temporal (49, 50, 56, 57, 58), and right-temporal (96, 100, 101, 107, 113)

The P50 ERP stimulus 1 (S1) was measured over the central (the average of channels 6, 7, 30, 31, 55, 80, 105, 106, which are roughly similar to C1, C2, FCZ and other central electrodes; Fig. 2), left-temporal (the average of channels 49, 50, 56, 57, 58, which are roughly similar to P7, TP7 and other left temporal–parietal electrodes; Fig. 2), and right-temporal (the average of channels 96, 100, 101, 107, 113, which are roughly similar to P10, CP10 and other right temporal–parietal electrodes; Fig. 2) regions, following inspection of the individual and grand averages. The central region of interest was chosen to expand the existing literature, which focuses primarily on CZ; thus, selecting a group of central electrodes allows us to explore whether sensory gating is observed in the central region in general, rather than just at CZ (For example, Park et al. 2015; Hunter et al. 2015). Additionally, prior literature (Korzyukov et al. 2007) proposed sensory gating ability could also be observed in the temporal regions, thus including a right- and left-temporal region of interest was incorporated. Upon visual inspection of the data, the P50 amplitude was visible in the temporal areas, supporting the inclusion of these regions. A time window of 150–230 ms was chosen for the left-temporal, 165–210 ms for the right-temporal, and 80–210 ms for the central electrodes.

### P50: stimulus 2

The P50 ERP stimulus 2 (S2) was measured over the central (the average of channels 6, 7, 30, 31, 55, 80, 105, 106; Fig. 2), left-temporal (the average of channels 49, 50, 56, 57, 58; Fig. 2), and right-temporal (the average of channels 96, 100, 101, 107, 113; Fig. 2) regions. A time window of 250–355 ms was chosen for the left-temporal, 260–335 ms for the right-temporal, and 260–355 ms for the central electrodes, following inspection of the individual and grand averages.

The time-windows chosen for the infant ERP’s were chosen following inspection of the individual and grand averages, and as such a latency effect was observed within the infant cohort, which differed slightly from the existing infancy P50 literature (Ross et al. 2013; Hunter et al. 2015).

### Questionnaires

#### Schizotypy

The Oxford-Inventory of Feelings and Experiences-Short Form (sO-LIFE; Mason et al. 2005) assessed schizotypy dimensionality and divided the participant cohort into iSZT and iCON. The sO-LIFE was chosen as the present measure of schizotypy dimensionality due to its fully dimensional approach, proposing that symptoms occurring in the schizophrenia-spectrum also occur in the typical population as well, with the sO-LIFE questionnaire measuring such symptoms. The reliability of the sO-LIFE, estimated with ordinal alpha, was disclosed to be above 0.78 (Fonseca-Pedrero et al. 2010). These levels of internal consistency are in line with the internal consistency values reported in previous studies; for example, previous work using ordinal alpha have found good reliability estimates (Lin et al. 2013; Ortuño-Sierra et al. 2013). Moreover, the sO-LIFE scores showed good convergent and discriminant validity with the Schizotypal Personality Questionnaire-brief revised (Goulding 2004; Mason et al. 1997; Burch et al. 2006). The mean across the present population was calculated (total *M *= 8.15, total SD = 6.26). The iSZT condition was determined by the *M* + 0.5 SD (sO-LIFE scores > 11.28) and included 14 participants and the iCON condition by the *M* − 0.5 SD (sO-LIFE Scores 5.02 > 0.0), included 21 participants.

#### Additional demographic variables

A general assessment questionnaire was used to gain an overall assessment of smoking habits, hearing deficits, birth complications, and whether they, or their family have experienced mental illness. Several independent samples *T* tests presented no significant differences between both iSZT and iCON groups (Table 1).

**Table 1:** A table to illustrate the demographic variables across both infant and adult cohorts

	Non-schizotypy *M* (SD)	Schizotypy *M* (SD)	*p* values
Infant age (days)	178.57 (8.07)	179.50 (9.70)	0.693
Infant gender			0.508
Female	*n* = 12	*n* = 6	
Male	*n* = 10	*n* = 8	
Mother’s age (years)	32.76 (3.11)	33.09 (5.48)	0.785
Maternal mental health experiences	1.14 (0.36)	1.43 (0.51)	0.061
Maternal family history of mental health	1.52 (0.51)	1.5 (0.52)	0.894
Birth complications	1.64 (0.79)	2.00 (0.96)	0.224

Note how the non-schizotypy and schizotypy groups in both infants and adults were age-matched and experienced no significant differences in mental health experiences

### Procedure

Prior to participation, the caregiver completed a series of questionnaires. The EEG cap was soaked in a warm water, sodium chloride solution and baby shampoo before fitting to the infant’s head prior to the infant falling asleep. Once fitted and following confirmation that each electrode responded to electrical activity, the trial procedure began. The auditory stimuli was presented 80-cm away, between 70 and 77 dB (Wan et al. 2008; Dalecki et al. 2011) until the infant woke or became restless. The infant was then left to complete their natural sleep period. Throughout the testing period the infant’s status was video-recorded to index activity. The mothers were invited back to participate in the same paradigm at a later date.

### Experiment 2: adult cohort

Experiment 1 showed no significant effects of maternal schizotypy dimensionality on sensory gating in infants although the infants did show significant differences between S1 and S2. The principal aim of experiment 2 was to examine these effects of schizotypy status on the mothers themselves.

#### Participants

Fifty-five mothers of the 6-month-old infants (*M* age = 32.9 years; SD= 4.25 years) participated. Fifty-three mothers were included in the final analysis following data editing, with exclusions due to sO-LIFE scores not identifying with one of the two groups (*n* = 2). The final sample included 23 participants identified as schizotypic mothers (SZT; *M* age = 33.09 years, SD= 5.48 years) and the remaining 30 participants were control mothers (CON; *M* age = 32.76 years, SD= 3.11 years). The entire maternal cohort were non-smokers. Recruitment and ethical approval was carried out using the same method as Experiment 1.

The same stimuli and materials, procedure, and EEG data reduction were used for Experiment 2 as per Experiment 1. The same criteria were used as with the infants to allow a direct comparison to be made between infant and mother data, although it could be assumed that the adults average trial contribution would be significantly more than that of the infants. Thus, participants required a minimum of 20% good trials for each stimuli to be included in further analyses. The adult cohort experienced a range of 56–64 paired-stimuli repetitions: equating to a minimum of 11–12 good trials, and contributed an average of 44.96 (SD = 7.11; range = 29–59) artefact-free trials for S1, and on average 45.20 (SD = 7.39; range = 25–57) artefact-free trials for S2. A paired-samples *t* test displayed no significant differences between the number of trials included for S1 and S2 (*t*(53) = − 0.486, *p* = 0.629).

### P50: stimulus 1

**Fig. 3:**
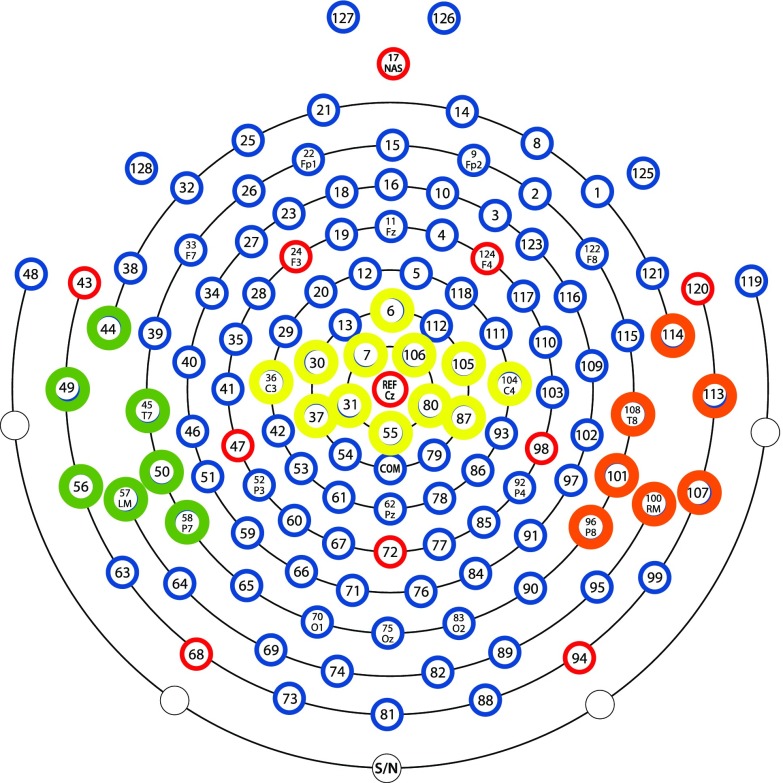
The P50 electrode groupings for the maternal cohort: central (6, 7, 30, 31, 36, 37, 55, 80, 87, 104, 105, 106), left-temporal (44, 45, 49, 50, 56, 57, 58), and right-temporal (96, 100, 101, 107, 108, 113, 114)

The P50 S1 was measured over the central (the average of channels 6, 7, 30, 31, 36, 37, 55, 80, 87, 104, 105, 106, which are roughly similar to C1, C2, FCZ and other central electrodes; Fig. 3), left-temporal (the average of channels 44, 45, 49, 50, 56, 57, 58, which are roughly similar to P7, TP7 and other left temporal–parietal electrodes; Fig. 3), and right-temporal (the average of channels 96, 100, 101, 107, 108, 113, 114, which are roughly similar to P10, CP10 and other right temporal-parietal electrodes; Fig. 3) regions. The central region of interest was again chosen to expand the existing literature, with prior literature (Korzyukov et al. 2007) suggesting that sensory gating ability could also be observed in the temporal regions, thus the inclusion of temporal regions of interest. A time window of 45–85 ms was chosen for the left-temporal, 50–80 ms for the right-temporal, and 45–90 ms for the central electrodes, following inspection of the individual and grand averages.

### P50: stimulus 2

The P50 S2 was measured over the central (the average of channels 6, 7, 30, 31, 36, 37, 55, 80, 87, 104, 105, 106; Fig. 3) left-temporal (the average of channels 44, 45, 49, 50, 56, 57, 58; Fig. 3), and right-temporal (the average of channels 96, 100, 101, 107, 108, 113, 114; Fig. 3) regions. A time window of 100–145 ms was chosen for the left-temporal, 105–140 ms for the right-temporal, and 100–145 ms for the central electrodes, following inspection of the individual and grand averages.

## Results

### Experiment 1: infant cohort

#### P50

**Fig. 4:**
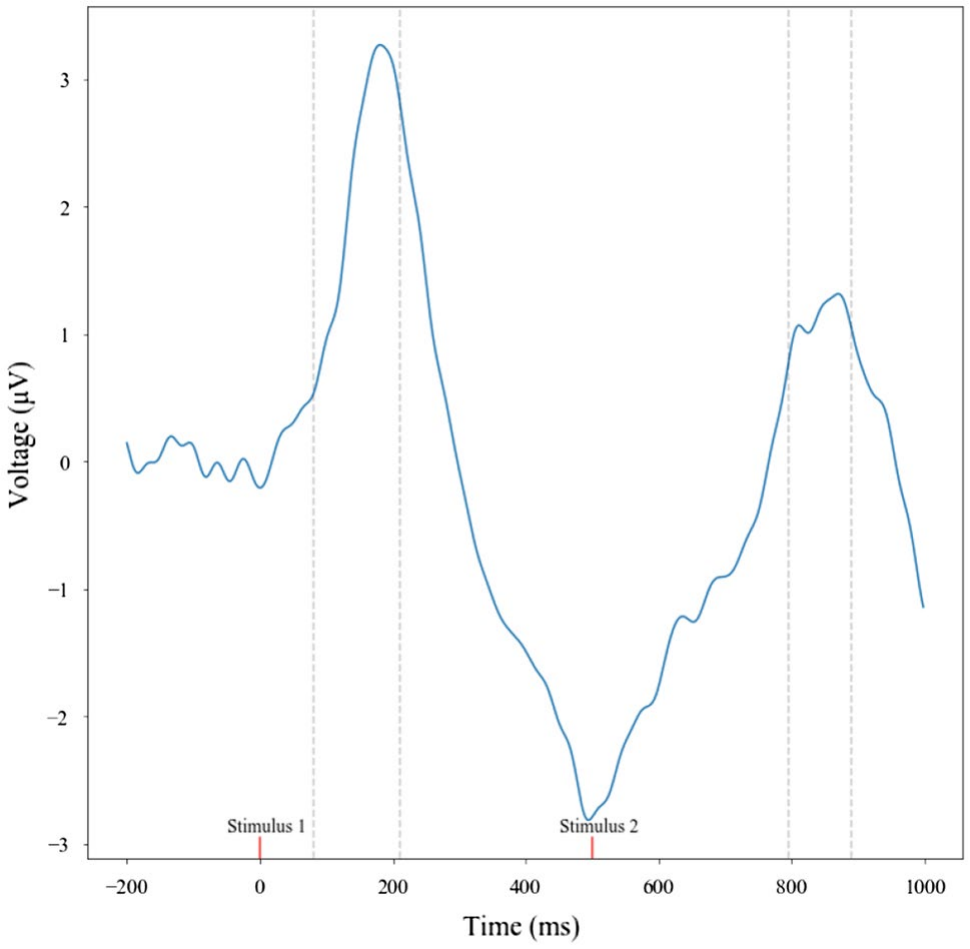
The P50 ERP component across the whole infant cohort in the central region. For the complete trial sequence, including S1 and S2, a time window of 80–210 ms can be observed for S1 in the central region and 760–855 ms for S2

A full factorial 2 (group: SZT or CON) × 2 (paired-tone: S1 or S2) × 3 (electrode grouping: central, left-temporal, or right-temporal) repeated-measures ANOVA with Bonferroni corrections for pairwise comparisons was carried out exploring both mean amplitude and maximum amplitude measures. Significant differences were observed in P50 amplitudes between the central, right-temporal, and left-temporal regions (*F*(2,66) = 12.467, *p* > 0.001, *η*^2^ = 0.274). To explore the differences between P50 amplitudes further, a paired-samples *t* test demonstrated a significant difference between S1 (maximum amplitude: *M* = 5.45, SD = 4.39) and S2 (maximum amplitude: *M* = 0.18, SD = 4.81) in the central region when examined using the maximum amplitude (*t*(34) = 2.062, *p* = 0.047) measure (Fig. 4). These effects were corrected for multiple comparisons using Bonferroni post-hoc tests. No further significant effects were found. No significant group differences were observed between the infants of schizotypic and infants of control mothers. In sum, significant differences in P50 amplitude were observed between stimulus 1 and stimulus 2 in the central region, but no further differences were detected.

A series of Pearson correlations were carried out to explore the relationship between the infants’ P50 ERP amplitude differences/suppression ratios and their mothers’ sO-LIFE scores. A significant relationship was observed between the mean amplitude suppression ratio in the right-temporal region and the sO-LIFE total score (*r* = − 0.347, *p* = 0.038), the Unusual Experiences dimension (*r* = − 0.410, *p* = 0.013), and the Cognitive Disorganisation dimension (*r* = − 0.362, *p* = 0.030).

### Experiment 2: maternal cohort

#### P50

A full factorial 2 (group: SZT or CON) × 2 (paired-tone: S1 or S2) × 3 (electrode grouping: central, left-temporal, or right-temporal) repeated-measures ANOVA with Bonferroni corrections for pairwise comparisons was carried out exploring both mean amplitude and maximum amplitude measures. A significant difference was observed between the P50 amplitudes produced for S1 and S2 (*F*(1,51) = 4.280, *p* = 0.044, *η*^2^ = 0.077), and a paired-tone by group interaction was also observed (*F*(1,51) = 6.171, *p* = 0.016, *η*^2^ = 0.108). A significant difference in P50 amplitude was observed between the different electrode regions of interest (*F*(2,102) = 150.055, *p* > 0.001, *η*^2^ = 0.746), and a paired-tone by region of interest interaction was also observed (*F*(2,102) = 2.008, *p* < 0.001, *η*^2^ = 0.038).

A paired-samples *t* test was used to follow-up these effects, and illustrated a significant difference between S1 (mean amplitude: *M* = 2.92, SD = 1.62; maximum amplitude: *M* = 4.11, SD = 1.73) and S2 (mean amplitude: *M* = 2.19, SD = 2.38; maximum amplitude: *M* = 3.12, SD = 2.37) in the left-temporal region when examined using the mean amplitude (*t*(52) = 2.39, *p* = 0.020) and maximum amplitude (*t*(52) = 3.24, *p* = 0.002) measures. These effects were corrected for multiple comparisons using Bonferroni post-hoc tests. Significant differences were also observed between S1 (mean amplitude: *M* = − 3.29, SD = 1.66; maximum amplitude: *M* = − 1.31, SD = 1.38) and S2 (mean amplitude: *M* = − 1.92, SD = 1.42; maximum amplitude: *M* = − 0.68, SD = 1.27) in the central region when examined using the mean amplitude (*t*(52) = − 7.81, *p* > 0.001) and maximum amplitude (*t*(52) = − 3.13, *p* = 0.003) measures. See Table 2 for a breakdown of the means and standard deviations associated with these significant differences.

**Table 2:** Mean and standard deviation between SZT and CON groups comparing S1 and S2

Electrode region	Measure	Group (*n*)	*M*	SD
Central	S1 mean amplitude	SZT (23)	− 3.367	1.944
		CON (30)	− 3.22	1.45
Central	S2 mean amplitude	SZT (23)	− 2.203	1.694
		CON (30)	− 1.708	1.144
Central	S1 maximum amplitude	SZT (23)	− 1.114	1.544
		CON (30)	− 1.453	1.242
Central	S2 maximum amplitude	SZT (23)	− 1.029	1.441
		CON (30)	− 0.419	1.069

An interim summary suggests that the maternal cohorts also illustrate amplitude differences between S1 and S2, with larger P50 amplitudes towards S1 as hypothesized from prior research.

**Fig. 5:**
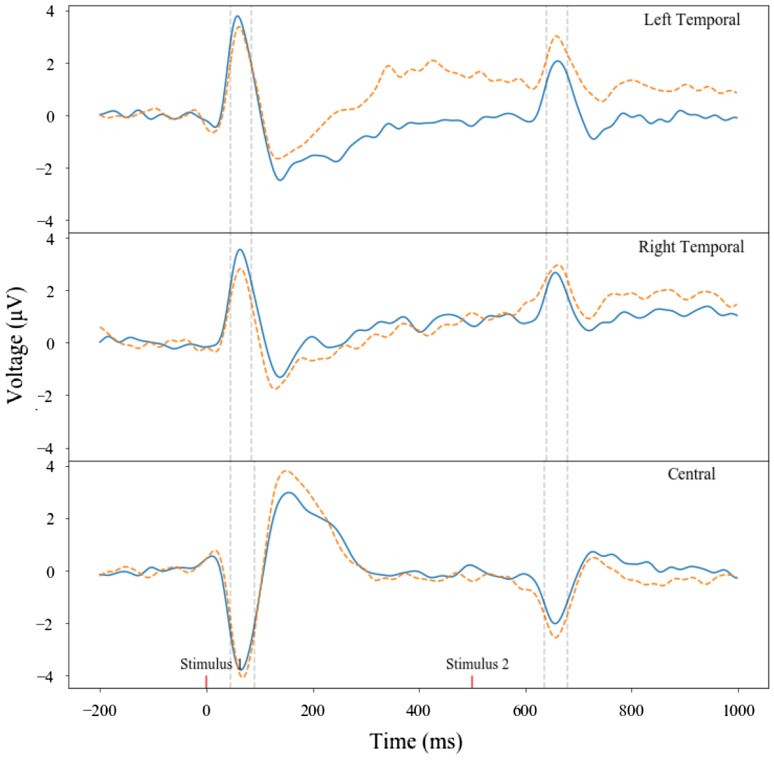
Maternal P50 mean amplitude paired-tone comparisons. Note how across the left-temporal, right-temporal, and central regions the SZT S1 and S2 peaks show smaller differences than the CON S1 and S2 peaks. For the complete trial sequence, time windows for stimulus 1 and stimulus 2 were chosen as 45–85 ms and 600–645 ms for left-temporal, 50–80 ms and 605–640 ms for right-temporal, and 45–90 ms and 600–645 ms for the central region. *SZT* dashed line, *CON* block line; Red block markers show S1 and S2 presentation

A significant difference between the amplitudes of S1 and S2 was observed in the mean amplitude measure in the left-temporal region (*F*(1,52) = 4.76, *p *= 0.034), with a trend towards a significant paired-tone by group interaction (*F*(1,51) = 3.69, *p *= 0.060). After the Bonferroni correction only a significant difference was observed between the pairwise comparisons made for S1 and S2 in the CON group (*p *= 0.003). A significant difference was observed between the paired-tones in the maximum amplitude measure in the left-temporal region also (*F*(1,51) = 9.23, *p *= 0.004), with a trend towards a significant paired-tone by group interaction (*F*(1,51) = 8.42, *p *= 0.064). After the Bonferroni correction only a significant difference was observed between the pairwise comparisons made for S1 and S2 in the CON group (*p *> 0.001). A significant difference was observed between the paired-tones in the maximum amplitude measure in the central region (*F*(1,51) = 8.56, *p *= 0.005), with a significant paired-tone by group interaction also observed (*F*(1,51) = 6.14, *p *= 0.017; Fig. 5). After the Bonferroni correction there was no significant pairwise comparisons between the two groups in S1, but a trend towards a difference between the two groups in S2 was observed (*p *= 0.083). Additionally, only a significant difference was observed between S1 and S2 in the CON group (*p *> 0.001).

In sum, significant differences were observed between S1 and S2 as predicted by a priori hypotheses. However, following corrections for multiple comparisons it was observed that only the CON mothers illustrated significant differences between amplitudes produced in response to S1 and S2; advocating an intact sensory gating ability, which contrasts the lack of amplitude differences between S1 and S2 for SZT mothers, suggesting they exhibit the sensory gating deficit observed across the schizophrenia-spectrum. A series of correlational analyses were conducted, see Table 3 for a summary of significant relationships.

**Table 3:** A summary of significant correlational relationships between the mothers ERP amplitudes and their sO-LIFE scores

	Central mean amplitude difference	Central maximum amplitude difference	Central mean amplitude suppression ratio	Right-temporal mean amplitude difference	Right-temporal maximum amplitude difference
sO-LIFE total score	–	*r *= 0.331, *p *= 0.017	–	–	–
Unusual experiences dimension	–	–	–	*r *= − 0.280, *p *= 0.044	–
Cognitive disorganisation dimension	–	*r *= 0.294, *p *= 0.034	–	–	–
Introvertive anhedonia dimension	*r *= 0.295, *p *= 0.034	–	*r *= − 0.548, *p *= 0.000	–	–
Impulsive non-conformity dimension	–	–	–	–	*r *= − 0.297, *p *= 0.032

The maternal P50 ERP observed in the central region illustrates a dipole difference that is observed across the regions that the present paper indexes. These dipole differences reflect a positive P50 peak in the temporal regions, but a negative peak at approximately 50 ms post stimulus is observed in the central region surrounding CZ. Thus, the differences reflected in this central region among the adults cohort is reflective of this dipole.

## General discussion

The present research investigated whether measurable changes in sensory gating function in the offspring of mothers with schizotypic traits could be detected in comparison to their control counterparts. Specifically, it was hypothesised that these mothers and their offspring would display smaller differences and larger ratios in the P50 event-related potential component. We have demonstrated two important findings in this research. Firstly, that sensory gating can be detected in infants as early as 6 months of age. Data revealed that although the 6-month-old infants’ P50 components displayed significant differences between S1 and S2, there was no clear difference between infants of schizotypic and infants of control mothers. Therefore, the infants of mothers presenting with schizotypic traits appear not to be at higher risk than normal, at least at 6 months of age.

Despite a lack of clear group differences in the 6-month cohort, a series of significant correlations were observed between suppression ratio/difference measures and the maternal sO-LIFE dimensions. This could be perceived as the beginning of differences between groups at this age. It is possible to conclude that these deficits are just not present at 6 months of age, or that maternal personality impacts the development of sensory gating, but this influence is not yet robust enough to illustrate clear group differences. Schizotypic traits are present in the general population and can go undetected by the unaided eye; thus, at 6 months it is likely that maternal schizotypy has not been extensively experienced enough to influence a measure as sensitive as sensory gating. Moreover, the event-related potential analysis utilised in this sensory gating paradigm may be hindered by the neuronal development of the 6-month-old infant. At this age, there are a quantity of neuronal and synaptic connections which are later pruned throughout development to adulthood to gain maximum efficiency (Singer 1995; Huttenlocher 2002). Thus, with increased neuronal connectivity, the EEG data collected and analysed are more ‘noisy’ than that collected by an adult cohort.

A second key finding was a clear dissociation in the brain activity of the SZT and CON mothers. The Bonferroni corrected pairwise comparisons illustrated how the CON mothers had significant differences between S1 and S2, illustrating typical sensory gating ability, whereas the lack of significant difference between the S1 and S2 for SZT mothers illustrates the sensory gating deficit observed across the schizophrenia-spectrum. This suggests that experiencing schizotypic traits, as characterised through the sO-LIFE, also influences sensory gating ability; whereby a smaller difference or larger suppression ratio is observed between S1 and S2. This supports prior literature (for example, Wan et al. 2017); whereby individuals who exhibit schizotypic traits also illustrate a reduced inability to inhibit, or ‘gate out’, the second tone in a paired-tone paradigm. The mothers experiencing schizotypic traits, may feel as though they would benefit from follow-up guidance, additional family support and education to assist them in mitigating any potential and future impact of their schizotypy status on their parenting skills.

Schizotypal expression during adolescence and adulthood is critically linked to childhood risk markers, which confer a role of potential developmental facilitators on the road to psychosis proneness (Debbané 2015, p. 88), thus, establishing brain-behaviour links in both clinically significant behaviours and those of typical development is an important step in further understanding the relationship between typical and pathological behaviour (Hengartner and Lehmann 2017). Prior literature focuses on deficits observed in schizophrenic patients and their biological relatives (for example, Ross and Freedman 2015), but a more recent shift in the literature explores the same deficits, albeit to a milder degree, in schizotypic individuals (for example, Debbané and Barrantes-Vidal 2015; Ross and Freedman 2015). These deficits can be described as endophenotypes and their continuous nature make it difficult to escape the conclusion that there is considerable overlap between the clinical schizophrenia-spectrum and sub-clinical schizotypy. Exploring endophenotypes among the sub-clinical realm of the spectrum is advantageous in removing the difficulties associated with schizophrenic cohorts, for example, medication. If schizotypic traits are present in the general population then it is also important to understand the influence these traits have on the people surrounding them; hence the focus of the present research. Moreover, the successful adaptation of tasks for use in early infancy will, therefore, increase our understanding of the developmental timeline of these disorders and perhaps allow for the development of novel prevention strategies.

To focus on the continuities and discontinuities that exist between typical and pathological behaviour, perhaps a focus on individual sub-dimensions would have provided a more accurate reflection of the relationship schizotypy has with the clinical continuum. This is a potential limitation of the present work. Focusing on individual sub-dimensions would have allowed for a direct mapping of the ‘positive’, ‘negative’, and ‘disorganised’ traits/symptoms outlined across the entire spectrum (e.g., Lenzenweger and Dworkin 1996; Kwapil et al. 2008). However, a lack of reliability in these measures is observed throughout the spectrum (for example, Cochrane et al. 2010). While the use of the combined dimensions total-score, as in the present research, does not provide a segregated reflection on the differential elements of schizotypy, it does nevertheless provide a way of ‘grouping’ schizotypic individuals. For future analyses, where exploring the continuity of endophenotypic traits/symptoms is a primary focus, addressing individual sub-dimensions of schizotypic personality may well be a more profitable approach.

It should be articulated that schizotypy, for the purpose of the present research, was defined using the sO-LIFE, with mothers classed as schizotypic if their sO-LIFE score was half a standard deviation above the total participant mean (as outlined previously). This approach was also adopted by Park et al. (2015) and weighs in favour of the fully dimensional approach: schizotypic features are observed in the general population and linked with typical development and atypical clinical disorders (Claridge et al. 1996). However, this could limit our ability to fully understand schizotypy as a personality construct. There is evidence that schizotypy is a construct with separable and well-identified components (Kwapil et al. 2008); thus, these dimensions, when combined, do not present a clear and distinguishable reflection of positive, negative, or disorganised schizotypy. The present experiment attempts to control for this limitation through the use of correlational analyses with the four separate dimensions, providing an additional measure of the four scales separately. Moving forward in the schizotypy literature, this is an important element to consider.

The sensory gating literature is unclear (Dalecki et al. 2011) with respect to the best method of suppression presentation and as such, the inclusion of both differences and suppression ratios within the analysis provides comparable clarity for understanding infant sensory gating; contrasting previous work that has relied on a single suppression parameter. Here, significant effects were observed in the suppression ratio scores in the infant population, and in both difference and suppression ratio measures in the maternal cohort. An additional strength, multiple electrode sites were utilised for analysis when contrasted with prior research, which explored sensory gating in the central regions, specifically CZ, and utilised a mastoid or earlobe reference (Toyomaki et al. 2015; Hunter et al. 2015; Thoma et al. 2017). An advantage of the current research is the quantity of electrodes in the high-density array. Upon visual inspection of both individual and grand averages, a clear P50 component could be observed in the central regions (Park et al. 2015), as predicted from prior literature, but also in the temporal regions as would be expected in concordance with prior auditory paradigms (Korzyukov et al. 2007). The current study also highlighted the complexity of recording electrical activity during sleep, where infants produce unpredictable movements, increasing quantities of artefacts and a reduced number of infants included in the final analysis. A future exploration could track, alongside the EEG P50 recordings, the sleep cycles of the infants, similarly to Hunter et al. (2015), to explore, for example, whether sensory gating is more efficient during different types of sleep.

A strength of this work was the non-specific differences in the demographic, social and clinical factors associated with the mothers, where the mothers and infants were matched across a range of demographic and clinical factors. This supports the hypothesis that the critical explanatory factor was the schizotypy status of the mother. Lack of specificity in the questionnaire responses restricted the analyses carried out to further understand the influence of prior mental illness on sensory gating ability. Perhaps a future replication could explore more detailed histories of mental illness in the adult populations to address whether schizotypy was more prevalent among those with a history of mental illness, as would be expected.

In summary, 6-month-old infants, in general, display the ability to gate out irrelevant stimuli. It is known that core neuropsychological dysfunctions for the potential development of clinical disorders are present during childhood and shape adult personality (Corr 2010). However, these relationships between the ERP differences and suppression ratio measures in the infants and the maternal sO-LIFE measures suggests a potential emergence of differences, which may be observed to a greater degree with continued developmental change.

## Electronic supplementary material

Below is the link to the electronic supplementary material.

Supplementary material 1 (DOC 22743 kb)
