# Supplementary material for: Is schizotypic maternal personality linked to sensory gating abilities during infancy?
Source: Exp Brain Res. 2019 May 13;237(7):1869–79. doi: 10.1007/s00221-019-05554-7 (PMC6584245; doi:10.1007/s00221-019-05554-7)
Supplement: Supplementary file 1 — Supplementary material 1 (DOC 22743 kb) [file 221_2019_5554_MOESM1_ESM.doc]

General Linear Model - 2 (group) 3 (region of interest) x 2 (paired-tone) RM-ANOVA exploring the P50 mean amplitudes in the infant cohort.

[DataSet1] /Volumes/NO NAME/SG_REDO_MaternalData/Infants_AUD_P50.sav


Within-Subjects Factors	
Measure:   MEASURE_1  	
RegionofInterest	PairedTone	Dependent Variable	
1	1	BEEP1_MEANAMP_LEFTtemporal	
	2	BEEP2_MEANAMP_LEFTtemporal	
2	1	BEEP1_MEANAMP_RIGHTtemporal	
	2	BEEP2_MEANAMP_RIGHTtemporal	
3	1	BEEP1_MEANAMP_CENTRAL	
	2	BEEP2_MEANAMP_CENTRAL	


Between-Subjects Factors	
	Value Label	N	
FINALGROUPS	1.00	SZT	14	
	2.00	CONTROL	21	


Descriptive Statistics	
	FINALGROUPS	Mean	Std. Deviation	N	
BEEP1_MEANAMP_LEFTtemporal	SZT	-2.7692	5.79274	14	
	CONTROL	-6.0481	6.36817	21	
	Total	-4.7365	6.27228	35	
BEEP2_MEANAMP_LEFTtemporal	SZT	-3.3288	3.94257	14	
	CONTROL	-3.7809	5.63827	21	
	Total	-3.6000	4.96929	35	
BEEP1_MEANAMP_RIGHTtemporal	SZT	-3.6646	7.72424	14	
	CONTROL	-3.8075	5.62157	21	
	Total	-3.7503	6.43484	35	
BEEP2_MEANAMP_RIGHTtemporal	SZT	-3.6267	8.05685	14	
	CONTROL	-2.2418	6.73899	21	
	Total	-2.7958	7.21163	35	
BEEP1_MEANAMP_CENTRAL	SZT	2.2991	3.66372	14	
	CONTROL	2.1740	4.42946	21	
	Total	2.2240	4.08379	35	
BEEP2_MEANAMP_CENTRAL	SZT	1.2822	6.18101	14	
	CONTROL	1.0132	5.58720	21	
	Total	1.1208	5.74355	35	


Box's Test of Equality of Covariance Matricesa	
Box's M	30.441	
F	1.142	
df1	21	
df2	2848.971	
Sig.	.295	

Tests the null hypothesis that the observed covariance matrices of the dependent variables are equal across groups.a	
a. Design: Intercept + FINALGROUPS 
 Within Subjects Design: RegionofInterest + PairedTone + RegionofInterest * PairedTone	


Multivariate Testsa	
Effect	Value	F	Hypothesis df	Error df	Sig.	
RegionofInterest	Pillai's Trace	.367	9.283b	2.000	32.000	.001	
	Wilks' Lambda	.633	9.283b	2.000	32.000	.001	
	Hotelling's Trace	.580	9.283b	2.000	32.000	.001	
	Roy's Largest Root	.580	9.283b	2.000	32.000	.001	
RegionofInterest * FINALGROUPS	Pillai's Trace	.047	.794b	2.000	32.000	.461	
	Wilks' Lambda	.953	.794b	2.000	32.000	.461	
	Hotelling's Trace	.050	.794b	2.000	32.000	.461	
	Roy's Largest Root	.050	.794b	2.000	32.000	.461	
PairedTone	Pillai's Trace	.005	.150b	1.000	33.000	.701	
	Wilks' Lambda	.995	.150b	1.000	33.000	.701	
	Hotelling's Trace	.005	.150b	1.000	33.000	.701	
	Roy's Largest Root	.005	.150b	1.000	33.000	.701	
PairedTone * FINALGROUPS	Pillai's Trace	.059	2.066b	1.000	33.000	.160	
	Wilks' Lambda	.941	2.066b	1.000	33.000	.160	
	Hotelling's Trace	.063	2.066b	1.000	33.000	.160	
	Roy's Largest Root	.063	2.066b	1.000	33.000	.160	
RegionofInterest * PairedTone	Pillai's Trace	.030	.503b	2.000	32.000	.609	
	Wilks' Lambda	.970	.503b	2.000	32.000	.609	
	Hotelling's Trace	.031	.503b	2.000	32.000	.609	
	Roy's Largest Root	.031	.503b	2.000	32.000	.609	
RegionofInterest * PairedTone * FINALGROUPS	Pillai's Trace	.027	.445b	2.000	32.000	.645	
	Wilks' Lambda	.973	.445b	2.000	32.000	.645	
	Hotelling's Trace	.028	.445b	2.000	32.000	.645	
	Roy's Largest Root	.028	.445b	2.000	32.000	.645	

Multivariate Testsa	
Effect	Partial Eta Squared	Noncent. Parameter	Observed Powerc	
RegionofInterest	Pillai's Trace	.367	18.565	.965	
	Wilks' Lambda	.367	18.565	.965	
	Hotelling's Trace	.367	18.565	.965	
	Roy's Largest Root	.367	18.565	.965	
RegionofInterest * FINALGROUPS	Pillai's Trace	.047	1.588	.173	
	Wilks' Lambda	.047	1.588	.173	
	Hotelling's Trace	.047	1.588	.173	
	Roy's Largest Root	.047	1.588	.173	
PairedTone	Pillai's Trace	.005	.150	.066	
	Wilks' Lambda	.005	.150	.066	
	Hotelling's Trace	.005	.150	.066	
	Roy's Largest Root	.005	.150	.066	
PairedTone * FINALGROUPS	Pillai's Trace	.059	2.066	.287	
	Wilks' Lambda	.059	2.066	.287	
	Hotelling's Trace	.059	2.066	.287	
	Roy's Largest Root	.059	2.066	.287	
RegionofInterest * PairedTone	Pillai's Trace	.030	1.006	.125	
	Wilks' Lambda	.030	1.006	.125	
	Hotelling's Trace	.030	1.006	.125	
	Roy's Largest Root	.030	1.006	.125	
RegionofInterest * PairedTone * FINALGROUPS	Pillai's Trace	.027	.889	.116	
	Wilks' Lambda	.027	.889	.116	
	Hotelling's Trace	.027	.889	.116	
	Roy's Largest Root	.027	.889	.116	

a. Design: Intercept + FINALGROUPS 
 Within Subjects Design: RegionofInterest + PairedTone + RegionofInterest * PairedTone	
b. Exact statistic	
c. Computed using alpha = .05	


Mauchly's Test of Sphericitya	
Measure:   MEASURE_1  	
Within Subjects Effect	Mauchly's W	Approx. Chi-Square	df	Sig.	Epsilonb	
					Greenhouse-Geisser	Huynh-Feldt	
RegionofInterest	.864	4.686	2	.096	.880	.954	
PairedTone	1.000	.000	0	.	1.000	1.000	
RegionofInterest * PairedTone	.586	17.102	2	.000	.707	.752	

Mauchly's Test of Sphericitya	
Measure:   MEASURE_1  	
Within Subjects Effect	Epsilon	
	Lower-bound	
RegionofInterest	.500	
PairedTone	1.000	
RegionofInterest * PairedTone	.500	

Tests the null hypothesis that the error covariance matrix of the orthonormalized transformed dependent variables is proportional to an identity matrix.a	
a. Design: Intercept + FINALGROUPS 
 Within Subjects Design: RegionofInterest + PairedTone + RegionofInterest * PairedTone	
b. May be used to adjust the degrees of freedom for the averaged tests of significance. Corrected tests are displayed in the Tests of Within-Subjects Effects table.	


Tests of Within-Subjects Effects	
Measure:   MEASURE_1  	
Source	Type III Sum of Squares	df	Mean Square	F	Sig.	
RegionofInterest	Sphericity Assumed	1296.619	2	648.309	12.467	.000	
	Greenhouse-Geisser	1296.619	1.760	736.625	12.467	.000	
	Huynh-Feldt	1296.619	1.908	679.546	12.467	.000	
	Lower-bound	1296.619	1.000	1296.619	12.467	.001	
RegionofInterest * FINALGROUPS	Sphericity Assumed	53.962	2	26.981	.519	.598	
	Greenhouse-Geisser	53.962	1.760	30.656	.519	.575	
	Huynh-Feldt	53.962	1.908	28.281	.519	.589	
	Lower-bound	53.962	1.000	53.962	.519	.476	
Error(RegionofInterest)	Sphericity Assumed	3432.092	66	52.001			
	Greenhouse-Geisser	3432.092	58.087	59.085			
	Huynh-Feldt	3432.092	62.966	54.507			
	Lower-bound	3432.092	33.000	104.003			
PairedTone	Sphericity Assumed	1.799	1	1.799	.150	.701	
	Greenhouse-Geisser	1.799	1.000	1.799	.150	.701	
	Huynh-Feldt	1.799	1.000	1.799	.150	.701	
	Lower-bound	1.799	1.000	1.799	.150	.701	
PairedTone * FINALGROUPS	Sphericity Assumed	24.821	1	24.821	2.066	.160	
	Greenhouse-Geisser	24.821	1.000	24.821	2.066	.160	
	Huynh-Feldt	24.821	1.000	24.821	2.066	.160	
	Lower-bound	24.821	1.000	24.821	2.066	.160	
Error(PairedTone)	Sphericity Assumed	396.394	33	12.012			
	Greenhouse-Geisser	396.394	33.000	12.012			
	Huynh-Feldt	396.394	33.000	12.012			
	Lower-bound	396.394	33.000	12.012			
RegionofInterest * PairedTone	Sphericity Assumed	41.165	2	20.583	.605	.549	
	Greenhouse-Geisser	41.165	1.414	29.104	.605	.495	
	Huynh-Feldt	41.165	1.504	27.371	.605	.505	
	Lower-bound	41.165	1.000	41.165	.605	.442	
RegionofInterest * PairedTone * FINALGROUPS	Sphericity Assumed	18.630	2	9.315	.274	.761	
	Greenhouse-Geisser	18.630	1.414	13.172	.274	.684	
	Huynh-Feldt	18.630	1.504	12.387	.274	.698	
	Lower-bound	18.630	1.000	18.630	.274	.604	
Error(RegionofInterest*PairedTone)	Sphericity Assumed	2244.971	66	34.015			
	Greenhouse-Geisser	2244.971	46.676	48.097			
	Huynh-Feldt	2244.971	49.632	45.232			
	Lower-bound	2244.971	33.000	68.029			

Tests of Within-Subjects Effects	
Measure:   MEASURE_1  	
Source	Partial Eta Squared	Noncent. Parameter	Observed Powera	
RegionofInterest	Sphericity Assumed	.274	24.934	.995	
	Greenhouse-Geisser	.274	21.945	.990	
	Huynh-Feldt	.274	23.788	.994	
	Lower-bound	.274	12.467	.929	
RegionofInterest * FINALGROUPS	Sphericity Assumed	.015	1.038	.132	
	Greenhouse-Geisser	.015	.913	.127	
	Huynh-Feldt	.015	.990	.130	
	Lower-bound	.015	.519	.108	
Error(RegionofInterest)	Sphericity Assumed				
	Greenhouse-Geisser				
	Huynh-Feldt				
	Lower-bound				
PairedTone	Sphericity Assumed	.005	.150	.066	
	Greenhouse-Geisser	.005	.150	.066	
	Huynh-Feldt	.005	.150	.066	
	Lower-bound	.005	.150	.066	
PairedTone * FINALGROUPS	Sphericity Assumed	.059	2.066	.287	
	Greenhouse-Geisser	.059	2.066	.287	
	Huynh-Feldt	.059	2.066	.287	
	Lower-bound	.059	2.066	.287	
Error(PairedTone)	Sphericity Assumed				
	Greenhouse-Geisser				
	Huynh-Feldt				
	Lower-bound				
RegionofInterest * PairedTone	Sphericity Assumed	.018	1.210	.147	
	Greenhouse-Geisser	.018	.856	.130	
	Huynh-Feldt	.018	.910	.133	
	Lower-bound	.018	.605	.118	
RegionofInterest * PairedTone * FINALGROUPS	Sphericity Assumed	.008	.548	.092	
	Greenhouse-Geisser	.008	.387	.085	
	Huynh-Feldt	.008	.412	.086	
	Lower-bound	.008	.274	.080	
Error(RegionofInterest*PairedTone)	Sphericity Assumed				
	Greenhouse-Geisser				
	Huynh-Feldt				
	Lower-bound				

a. Computed using alpha = .05	


Tests of Within-Subjects Contrasts	
Measure:   MEASURE_1  	
Source	RegionofInterest	PairedTone	Type III Sum of Squares	df	Mean Square	F	
RegionofInterest	Linear		1081.684	1	1081.684	18.693	
	Quadratic		214.935	1	214.935	4.659	
RegionofInterest * FINALGROUPS	Linear		23.385	1	23.385	.404	
	Quadratic		30.577	1	30.577	.663	
Error(RegionofInterest)	Linear		1909.579	33	57.866		
	Quadratic		1522.513	33	46.137		
PairedTone		Linear	1.799	1	1.799	.150	
PairedTone * FINALGROUPS		Linear	24.821	1	24.821	2.066	
Error(PairedTone)		Linear	396.394	33	12.012		
RegionofInterest * PairedTone	Linear	Linear	31.700	1	31.700	1.007	
	Quadratic	Linear	9.465	1	9.465	.259	
RegionofInterest * PairedTone * FINALGROUPS	Linear	Linear	18.533	1	18.533	.589	
	Quadratic	Linear	.097	1	.097	.003	
Error(RegionofInterest*PairedTone)	Linear	Linear	1039.214	33	31.491		
	Quadratic	Linear	1205.757	33	36.538		

Tests of Within-Subjects Contrasts	
Measure:   MEASURE_1  	
Source	RegionofInterest	PairedTone	Sig.	Partial Eta Squared	Noncent. Parameter	Observed Powera	
RegionofInterest	Linear		.000	.362	18.693	.987	
	Quadratic		.038	.124	4.659	.554	
RegionofInterest * FINALGROUPS	Linear		.529	.012	.404	.095	
	Quadratic		.421	.020	.663	.124	
Error(RegionofInterest)	Linear						
	Quadratic						
PairedTone		Linear	.701	.005	.150	.066	
PairedTone * FINALGROUPS		Linear	.160	.059	2.066	.287	
Error(PairedTone)		Linear					
RegionofInterest * PairedTone	Linear	Linear	.323	.030	1.007	.164	
	Quadratic	Linear	.614	.008	.259	.078	
RegionofInterest * PairedTone * FINALGROUPS	Linear	Linear	.448	.018	.589	.116	
	Quadratic	Linear	.959	.000	.003	.050	
Error(RegionofInterest*PairedTone)	Linear	Linear					
	Quadratic	Linear					

a. Computed using alpha = .05	


Levene's Test of Equality of Error Variancesa	
	F	df1	df2	Sig.	
BEEP1_MEANAMP_LEFTtemporal	.457	1	33	.504	
BEEP2_MEANAMP_LEFTtemporal	.867	1	33	.359	
BEEP1_MEANAMP_RIGHTtemporal	2.325	1	33	.137	
BEEP2_MEANAMP_RIGHTtemporal	1.139	1	33	.294	
BEEP1_MEANAMP_CENTRAL	.691	1	33	.412	
BEEP2_MEANAMP_CENTRAL	.335	1	33	.567	

Tests the null hypothesis that the error variance of the dependent variable is equal across groups.a	
a. Design: Intercept + FINALGROUPS 
 Within Subjects Design: RegionofInterest + PairedTone + RegionofInterest * PairedTone	


Tests of Between-Subjects Effects	
Measure:   MEASURE_1  	
Transformed Variable:   Average  	
Source	Type III Sum of Squares	df	Mean Square	F	Sig.	Partial Eta Squared	Noncent. Parameter	
Intercept	708.688	1	708.688	27.212	.000	.452	27.212	
FINALGROUPS	11.637	1	11.637	.447	.508	.013	.447	
Error	859.427	33	26.043					

Tests of Between-Subjects Effects	
Measure:   MEASURE_1  	
Transformed Variable:   Average  	
Source	Observed Powera	
Intercept	.999	
FINALGROUPS	.100	
Error		

a. Computed using alpha = .05	


Estimated Marginal Means

1. Grand Mean	
Measure:   MEASURE_1  	
Mean	Std. Error	95% Confidence Interval	
		Lower Bound	Upper Bound	
-1.875	.359	-2.606	-1.144	


2. FINALGROUPS

Estimates	
Measure:   MEASURE_1  	
FINALGROUPS	Mean	Std. Error	95% Confidence Interval	
			Lower Bound	Upper Bound	
SZT	-1.635	.557	-2.768	-.502	
CONTROL	-2.115	.455	-3.040	-1.190	


Pairwise Comparisons	
Measure:   MEASURE_1  	
(I) FINALGROUPS	(J) FINALGROUPS	Mean Difference (I-J)	Std. Error	Sig.a	95% Confidence Interval for Differencea	
					Lower Bound	Upper Bound	
SZT	CONTROL	.481	.719	.508	-.982	1.943	
CONTROL	SZT	-.481	.719	.508	-1.943	.982	

Based on estimated marginal means	
a. Adjustment for multiple comparisons: Bonferroni.	


Univariate Tests	
Measure:   MEASURE_1  	
	Sum of Squares	df	Mean Square	F	Sig.	Partial Eta Squared	Noncent. Parameter	
Contrast	1.940	1	1.940	.447	.508	.013	.447	
Error	143.238	33	4.341					

Univariate Tests	
Measure:   MEASURE_1  	
	Observed Powera	
Contrast	.100	
Error		

The F tests the effect of FINALGROUPS. This test is based on the linearly independent pairwise comparisons among the estimated marginal means.	
a. Computed using alpha = .05	

3. RegionofInterest

Estimates	
Measure:   MEASURE_1  	
RegionofInterest	Mean	Std. Error	95% Confidence Interval	
			Lower Bound	Upper Bound	
1	-3.982	.841	-5.693	-2.270	
2	-3.335	.922	-5.211	-1.459	
3	1.692	.614	.442	2.942	

Pairwise Comparisons	
Measure:   MEASURE_1  	
(I) RegionofInterest	(J) RegionofInterest	Mean Difference (I-J)	Std. Error	Sig.b	95% Confidence Interval for Differenceb	
					Lower Bound	Upper Bound	
1	2	-.647	.995	1.000	-3.155	1.862	
	3	-5.674*	1.312	.000	-8.984	-2.364	
2	1	.647	.995	1.000	-1.862	3.155	
	3	-5.027*	1.390	.003	-8.533	-1.522	
3	1	5.674*	1.312	.000	2.364	8.984	
	2	5.027*	1.390	.003	1.522	8.533	

Based on estimated marginal means	
*. The mean difference is significant at the .05 level.	
b. Adjustment for multiple comparisons: Bonferroni.	

Multivariate Tests	
	Value	F	Hypothesis df	Error df	Sig.	Partial Eta Squared	Noncent. Parameter	
Pillai's trace	.367	9.283a	2.000	32.000	.001	.367	18.565	
Wilks' lambda	.633	9.283a	2.000	32.000	.001	.367	18.565	
Hotelling's trace	.580	9.283a	2.000	32.000	.001	.367	18.565	
Roy's largest root	.580	9.283a	2.000	32.000	.001	.367	18.565	

Multivariate Tests	
	Observed Powerb	
Pillai's trace	.965	
Wilks' lambda	.965	
Hotelling's trace	.965	
Roy's largest root	.965	

Each F tests the multivariate effect of RegionofInterest. These tests are based on the linearly independent pairwise comparisons among the estimated marginal means.	
a. Exact statistic	
b. Computed using alpha = .05	

4. PairedTone

Estimates	
Measure:   MEASURE_1  	
PairedTone	Mean	Std. Error	95% Confidence Interval	
			Lower Bound	Upper Bound	
1	-1.969	.481	-2.948	-.991	
2	-1.780	.382	-2.558	-1.003	

Pairwise Comparisons	
Measure:   MEASURE_1  	
(I) PairedTone	(J) PairedTone	Mean Difference (I-J)	Std. Error	Sig.a	95% Confidence Interval for Differencea	
					Lower Bound	Upper Bound	
1	2	-.189	.488	.701	-1.182	.804	
2	1	.189	.488	.701	-.804	1.182	

Based on estimated marginal means	
a. Adjustment for multiple comparisons: Bonferroni.	


Multivariate Tests	
	Value	F	Hypothesis df	Error df	Sig.	Partial Eta Squared	Noncent. Parameter	
Pillai's trace	.005	.150a	1.000	33.000	.701	.005	.150	
Wilks' lambda	.995	.150a	1.000	33.000	.701	.005	.150	
Hotelling's trace	.005	.150a	1.000	33.000	.701	.005	.150	
Roy's largest root	.005	.150a	1.000	33.000	.701	.005	.150	

Multivariate Tests	
	Observed Powerb	
Pillai's trace	.066	
Wilks' lambda	.066	
Hotelling's trace	.066	
Roy's largest root	.066	

Each F tests the multivariate effect of PairedTone. These tests are based on the linearly independent pairwise comparisons among the estimated marginal means.	
a. Exact statistic	
b. Computed using alpha = .05	


5. FINALGROUPS * RegionofInterest	
Measure:   MEASURE_1  	
FINALGROUPS	RegionofInterest	Mean	Std. Error	95% Confidence Interval	
				Lower Bound	Upper Bound	
SZT	1	-3.049	1.303	-5.701	-.397	
	2	-3.646	1.428	-6.552	-.740	
	3	1.791	.952	-.145	3.727	
CONTROL	1	-4.915	1.064	-7.080	-2.749	
	2	-3.025	1.166	-5.397	-.652	
	3	1.594	.777	.013	3.174	


6. FINALGROUPS * PairedTone	
Measure:   MEASURE_1  	
FINALGROUPS	PairedTone	Mean	Std. Error	95% Confidence Interval	
				Lower Bound	Upper Bound	
SZT	1	-1.378	.745	-2.894	.138	
	2	-1.891	.592	-3.096	-.686	
CONTROL	1	-2.561	.608	-3.798	-1.323	
	2	-1.670	.484	-2.654	-.686	


7. RegionofInterest * PairedTone	
Measure:   MEASURE_1  	
RegionofInterest	PairedTone	Mean	Std. Error	95% Confidence Interval	
				Lower Bound	Upper Bound	
1	1	-4.409	1.061	-6.566	-2.251	
	2	-3.555	.869	-5.323	-1.786	
2	1	-3.736	1.127	-6.028	-1.444	
	2	-2.934	1.257	-5.492	-.377	
3	1	2.237	.715	.782	3.691	
	2	1.148	1.005	-.898	3.193	


8. FINALGROUPS * RegionofInterest * PairedTone	
Measure:   MEASURE_1  	
FINALGROUPS	RegionofInterest	PairedTone	Mean	Std. Error	95% Confidence Interval	
					Lower Bound	Upper Bound	
SZT	1	1	-2.769	1.643	-6.112	.574	
		2	-3.329	1.347	-6.069	-.589	
	2	1	-3.665	1.746	-7.216	-.113	
		2	-3.627	1.947	-7.589	.335	
	3	1	2.299	1.108	.045	4.553	
		2	1.282	1.558	-1.887	4.451	
CONTROL	1	1	-6.048	1.342	-8.778	-3.319	
		2	-3.781	1.100	-6.018	-1.544	
	2	1	-3.807	1.425	-6.707	-.908	
		2	-2.242	1.590	-5.477	.993	
	3	1	2.174	.904	.334	4.014	
		2	1.013	1.272	-1.574	3.601	

General Linear Model - 2 (group) 3 (region of 
    interest) x 2 (paired-tone) RM-ANOVA exploring the P50 maximum amplitudes in 
    the infant cohort.


Within-Subjects Factors	
Measure:   MEASURE_1  	
RegionofInterest	PairedTone	Dependent Variable	
1	1	BEEP1_MAXAMP_CENTRAL	
	2	BEEP2_MAXAMP_CENTRAL	
2	1	BEEP1_MAXAMP_LEFTtemporal	
	2	BEEP2_MAXAMP_LEFTtemporal	
3	1	BEEP1_MAXAMP_RIGHTtemporal	
	2	BEEP2_MAXAMP_RIGHTtemporal	


Between-Subjects Factors	
	Value Label	N	
FINALGROUPS	1.00	SZT	14	
	2.00	CONTROL	21	


Descriptive Statistics	
	FINALGROUPS	Mean	Std. Deviation	N	
BEEP1_MAXAMP_CENTRAL	SZT	5.5203	3.86024	14	
	CONTROL	5.5485	4.85131	21	
	Total	5.5372	4.42064	35	
BEEP2_MAXAMP_CENTRAL	SZT	3.6150	5.84864	14	
	CONTROL	2.8519	5.49569	21	
	Total	3.1571	5.56679	35	
BEEP1_MAXAMP_LEFTtemporal	SZT	1.6875	5.32572	14	
	CONTROL	-1.5636	5.05136	21	
	Total	-.2632	5.33531	35	
BEEP2_MAXAMP_LEFTtemporal	SZT	.7430	4.09066	14	
	CONTROL	-.4444	5.39952	21	
	Total	.0306	4.88839	35	
BEEP1_MAXAMP_RIGHTtemporal	SZT	-.2364	7.63313	14	
	CONTROL	-.9120	5.60005	21	
	Total	-.6418	6.39045	35	
BEEP2_MAXAMP_RIGHTtemporal	SZT	-.4367	7.41079	14	
	CONTROL	.9341	6.27861	21	
	Total	.3858	6.68220	35	


Box's Test of Equality of Covariance Matricesa	
Box's M	35.828	
F	1.344	
df1	21	
df2	2848.971	
Sig.	.135	

Tests the null hypothesis that the observed covariance matrices of the dependent variables are equal across groups.a	
a. Design: Intercept + FINALGROUPS 
 Within Subjects Design: RegionofInterest + PairedTone + RegionofInterest * PairedTone	

Multivariate Testsa	
Effect	Value	F	Hypothesis df	Error df	Sig.	
RegionofInterest	Pillai's Trace	.293	6.644b	2.000	32.000	.004	
	Wilks' Lambda	.707	6.644b	2.000	32.000	.004	
	Hotelling's Trace	.415	6.644b	2.000	32.000	.004	
	Roy's Largest Root	.415	6.644b	2.000	32.000	.004	
RegionofInterest * FINALGROUPS	Pillai's Trace	.053	.887b	2.000	32.000	.422	
	Wilks' Lambda	.947	.887b	2.000	32.000	.422	
	Hotelling's Trace	.055	.887b	2.000	32.000	.422	
	Roy's Largest Root	.055	.887b	2.000	32.000	.422	
PairedTone	Pillai's Trace	.029	.974b	1.000	33.000	.331	
	Wilks' Lambda	.971	.974b	1.000	33.000	.331	
	Hotelling's Trace	.030	.974b	1.000	33.000	.331	
	Roy's Largest Root	.030	.974b	1.000	33.000	.331	
PairedTone * FINALGROUPS	Pillai's Trace	.040	1.387b	1.000	33.000	.247	
	Wilks' Lambda	.960	1.387b	1.000	33.000	.247	
	Hotelling's Trace	.042	1.387b	1.000	33.000	.247	
	Roy's Largest Root	.042	1.387b	1.000	33.000	.247	
RegionofInterest * PairedTone	Pillai's Trace	.055	.933b	2.000	32.000	.404	
	Wilks' Lambda	.945	.933b	2.000	32.000	.404	
	Hotelling's Trace	.058	.933b	2.000	32.000	.404	
	Roy's Largest Root	.058	.933b	2.000	32.000	.404	
RegionofInterest * PairedTone * FINALGROUPS	Pillai's Trace	.020	.322b	2.000	32.000	.727	
	Wilks' Lambda	.980	.322b	2.000	32.000	.727	
	Hotelling's Trace	.020	.322b	2.000	32.000	.727	
	Roy's Largest Root	.020	.322b	2.000	32.000	.727	

Multivariate Testsa	
Effect	Partial Eta Squared	Noncent. Parameter	Observed Powerc	
RegionofInterest	Pillai's Trace	.293	13.287	.885	
	Wilks' Lambda	.293	13.287	.885	
	Hotelling's Trace	.293	13.287	.885	
	Roy's Largest Root	.293	13.287	.885	
RegionofInterest * FINALGROUPS	Pillai's Trace	.053	1.774	.189	
	Wilks' Lambda	.053	1.774	.189	
	Hotelling's Trace	.053	1.774	.189	
	Roy's Largest Root	.053	1.774	.189	
PairedTone	Pillai's Trace	.029	.974	.160	
	Wilks' Lambda	.029	.974	.160	
	Hotelling's Trace	.029	.974	.160	
	Roy's Largest Root	.029	.974	.160	
PairedTone * FINALGROUPS	Pillai's Trace	.040	1.387	.208	
	Wilks' Lambda	.040	1.387	.208	
	Hotelling's Trace	.040	1.387	.208	
	Roy's Largest Root	.040	1.387	.208	
RegionofInterest * PairedTone	Pillai's Trace	.055	1.865	.197	
	Wilks' Lambda	.055	1.865	.197	
	Hotelling's Trace	.055	1.865	.197	
	Roy's Largest Root	.055	1.865	.197	
RegionofInterest * PairedTone * FINALGROUPS	Pillai's Trace	.020	.644	.097	
	Wilks' Lambda	.020	.644	.097	
	Hotelling's Trace	.020	.644	.097	
	Roy's Largest Root	.020	.644	.097	

a. Design: Intercept + FINALGROUPS 
 Within Subjects Design: RegionofInterest + PairedTone + RegionofInterest * PairedTone	
b. Exact statistic	
c. Computed using alpha = .05	


Mauchly's Test of Sphericitya	
Measure:   MEASURE_1  	
Within Subjects Effect	Mauchly's W	Approx. Chi-Square	df	Sig.	Epsilonb	
					Greenhouse-Geisser	Huynh-Feldt	
RegionofInterest	.886	3.860	2	.145	.898	.975	
PairedTone	1.000	.000	0	.	1.000	1.000	
RegionofInterest * PairedTone	.638	14.381	2	.001	.734	.783	

Mauchly's Test of Sphericitya	
Measure:   MEASURE_1  	
Within Subjects Effect	Epsilon	
	Lower-bound	
RegionofInterest	.500	
PairedTone	1.000	
RegionofInterest * PairedTone	.500	

Tests the null hypothesis that the error covariance matrix of the orthonormalized transformed dependent variables is proportional to an identity matrix.a	
a. Design: Intercept + FINALGROUPS 
 Within Subjects Design: RegionofInterest + PairedTone + RegionofInterest * PairedTone	
b. May be used to adjust the degrees of freedom for the averaged tests of significance. Corrected tests are displayed in the Tests of Within-Subjects Effects table.	


Tests of Within-Subjects Effects	
Measure:   MEASURE_1  	
Source	Type III Sum of Squares	df	Mean Square	F	Sig.	
RegionofInterest	Sphericity Assumed	874.677	2	437.339	9.160	.000	
	Greenhouse-Geisser	874.677	1.796	487.040	9.160	.001	
	Huynh-Feldt	874.677	1.950	448.489	9.160	.000	
	Lower-bound	874.677	1.000	874.677	9.160	.005	
RegionofInterest * FINALGROUPS	Sphericity Assumed	58.960	2	29.480	.617	.542	
	Greenhouse-Geisser	58.960	1.796	32.830	.617	.526	
	Huynh-Feldt	58.960	1.950	30.232	.617	.539	
	Lower-bound	58.960	1.000	58.960	.617	.438	
Error(RegionofInterest)	Sphericity Assumed	3151.083	66	47.744			
	Greenhouse-Geisser	3151.083	59.265	53.170			
	Huynh-Feldt	3151.083	64.359	48.961			
	Lower-bound	3151.083	33.000	95.487			
PairedTone	Sphericity Assumed	10.831	1	10.831	.974	.331	
	Greenhouse-Geisser	10.831	1.000	10.831	.974	.331	
	Huynh-Feldt	10.831	1.000	10.831	.974	.331	
	Lower-bound	10.831	1.000	10.831	.974	.331	
PairedTone * FINALGROUPS	Sphericity Assumed	15.421	1	15.421	1.387	.247	
	Greenhouse-Geisser	15.421	1.000	15.421	1.387	.247	
	Huynh-Feldt	15.421	1.000	15.421	1.387	.247	
	Lower-bound	15.421	1.000	15.421	1.387	.247	
Error(PairedTone)	Sphericity Assumed	366.794	33	11.115			
	Greenhouse-Geisser	366.794	33.000	11.115			
	Huynh-Feldt	366.794	33.000	11.115			
	Lower-bound	366.794	33.000	11.115			
RegionofInterest * PairedTone	Sphericity Assumed	89.620	2	44.810	1.384	.258	
	Greenhouse-Geisser	89.620	1.468	61.031	1.384	.256	
	Huynh-Feldt	89.620	1.567	57.209	1.384	.257	
	Lower-bound	89.620	1.000	89.620	1.384	.248	
RegionofInterest * PairedTone * FINALGROUPS	Sphericity Assumed	22.684	2	11.342	.350	.706	
	Greenhouse-Geisser	22.684	1.468	15.448	.350	.640	
	Huynh-Feldt	22.684	1.567	14.480	.350	.654	
	Lower-bound	22.684	1.000	22.684	.350	.558	
Error(RegionofInterest*PairedTone)	Sphericity Assumed	2136.810	66	32.376			
	Greenhouse-Geisser	2136.810	48.458	44.096			
	Huynh-Feldt	2136.810	51.695	41.335			
	Lower-bound	2136.810	33.000	64.752			

Tests of Within-Subjects Effects	
Measure:   MEASURE_1  	
Source	Partial Eta Squared	Noncent. Parameter	Observed Powera	
RegionofInterest	Sphericity Assumed	.217	18.320	.971	
	Greenhouse-Geisser	.217	16.451	.958	
	Huynh-Feldt	.217	17.865	.968	
	Lower-bound	.217	9.160	.836	
RegionofInterest * FINALGROUPS	Sphericity Assumed	.018	1.235	.149	
	Greenhouse-Geisser	.018	1.109	.143	
	Huynh-Feldt	.018	1.204	.148	
	Lower-bound	.018	.617	.119	
Error(RegionofInterest)	Sphericity Assumed				
	Greenhouse-Geisser				
	Huynh-Feldt				
	Lower-bound				
PairedTone	Sphericity Assumed	.029	.974	.160	
	Greenhouse-Geisser	.029	.974	.160	
	Huynh-Feldt	.029	.974	.160	
	Lower-bound	.029	.974	.160	
PairedTone * FINALGROUPS	Sphericity Assumed	.040	1.387	.208	
	Greenhouse-Geisser	.040	1.387	.208	
	Huynh-Feldt	.040	1.387	.208	
	Lower-bound	.040	1.387	.208	
Error(PairedTone)	Sphericity Assumed				
	Greenhouse-Geisser				
	Huynh-Feldt				
	Lower-bound				
RegionofInterest * PairedTone	Sphericity Assumed	.040	2.768	.287	
	Greenhouse-Geisser	.040	2.032	.247	
	Huynh-Feldt	.040	2.168	.254	
	Lower-bound	.040	1.384	.208	
RegionofInterest * PairedTone * FINALGROUPS	Sphericity Assumed	.011	.701	.104	
	Greenhouse-Geisser	.011	.514	.096	
	Huynh-Feldt	.011	.549	.098	
	Lower-bound	.011	.350	.089	
Error(RegionofInterest*PairedTone)	Sphericity Assumed				
	Greenhouse-Geisser				
	Huynh-Feldt				
	Lower-bound				

a. Computed using alpha = .05	


Tests of Within-Subjects Contrasts	
Measure:   MEASURE_1  	
Source	RegionofInterest	PairedTone	Type III Sum of Squares	df	Mean Square	F	
RegionofInterest	Linear		694.590	1	694.590	12.096	
	Quadratic		180.087	1	180.087	4.731	
RegionofInterest * FINALGROUPS	Linear		4.294	1	4.294	.075	
	Quadratic		54.665	1	54.665	1.436	
Error(RegionofInterest)	Linear		1894.892	33	57.421		
	Quadratic		1256.191	33	38.066		
PairedTone		Linear	10.831	1	10.831	.974	
PairedTone * FINALGROUPS		Linear	15.421	1	15.421	1.387	
Error(PairedTone)		Linear	366.794	33	11.115		
RegionofInterest * PairedTone	Linear	Linear	81.971	1	81.971	1.593	
	Quadratic	Linear	7.649	1	7.649	.575	
RegionofInterest * PairedTone * FINALGROUPS	Linear	Linear	16.910	1	16.910	.329	
	Quadratic	Linear	5.774	1	5.774	.434	
Error(RegionofInterest*PairedTone)	Linear	Linear	1697.937	33	51.453		
	Quadratic	Linear	438.874	33	13.299		

Tests of Within-Subjects Contrasts	
Measure:   MEASURE_1  	
Source	RegionofInterest	PairedTone	Sig.	Partial Eta Squared	Noncent. Parameter	Observed Powera	
RegionofInterest	Linear		.001	.268	12.096	.921	
	Quadratic		.037	.125	4.731	.560	
RegionofInterest * FINALGROUPS	Linear		.786	.002	.075	.058	
	Quadratic		.239	.042	1.436	.214	
Error(RegionofInterest)	Linear						
	Quadratic						
PairedTone		Linear	.331	.029	.974	.160	
PairedTone * FINALGROUPS		Linear	.247	.040	1.387	.208	
Error(PairedTone)		Linear					
RegionofInterest * PairedTone	Linear	Linear	.216	.046	1.593	.232	
	Quadratic	Linear	.454	.017	.575	.114	
RegionofInterest * PairedTone * FINALGROUPS	Linear	Linear	.570	.010	.329	.086	
	Quadratic	Linear	.515	.013	.434	.098	
Error(RegionofInterest*PairedTone)	Linear	Linear					
	Quadratic	Linear					

a. Computed using alpha = .05	


Levene's Test of Equality of Error Variancesa	
	F	df1	df2	Sig.	
BEEP1_MAXAMP_CENTRAL	.767	1	33	.387	
BEEP2_MAXAMP_CENTRAL	.032	1	33	.860	
BEEP1_MAXAMP_LEFTtemporal	.400	1	33	.531	
BEEP2_MAXAMP_LEFTtemporal	.725	1	33	.401	
BEEP1_MAXAMP_RIGHTtemporal	2.570	1	33	.118	
BEEP2_MAXAMP_RIGHTtemporal	1.429	1	33	.241	

Tests the null hypothesis that the error variance of the dependent variable is equal across groups.a	
a. Design: Intercept + FINALGROUPS 
 Within Subjects Design: RegionofInterest + PairedTone + RegionofInterest * PairedTone	


Tests of Between-Subjects Effects	
Measure:   MEASURE_1  	
Transformed Variable:   Average  	
Source	Type III Sum of Squares	df	Mean Square	F	Sig.	Partial Eta Squared	Noncent. Parameter	
Intercept	419.356	1	419.356	22.136	.000	.401	22.136	
FINALGROUPS	28.076	1	28.076	1.482	.232	.043	1.482	
Error	625.183	33	18.945					

Tests of Between-Subjects Effects	
Measure:   MEASURE_1  	
Transformed Variable:   Average  	
Source	Observed Powera	
Intercept	.995	
FINALGROUPS	.219	
Error		

a. Computed using alpha = .05	


Estimated Marginal Means

1. Grand Mean	
Measure:   MEASURE_1  	
Mean	Std. Error	95% Confidence Interval	
		Lower Bound	Upper Bound	
1.442	.307	.819	2.066	


2. FINALGROUPS

Estimates	
Measure:   MEASURE_1  	
FINALGROUPS	Mean	Std. Error	95% Confidence Interval	
			Lower Bound	Upper Bound	
SZT	1.815	.475	.849	2.782	
CONTROL	1.069	.388	.280	1.858	


Pairwise Comparisons	
Measure:   MEASURE_1  	
(I) FINALGROUPS	(J) FINALGROUPS	Mean Difference (I-J)	Std. Error	Sig.a	95% Confidence Interval for Differencea	
					Lower Bound	Upper Bound	
SZT	CONTROL	.746	.613	.232	-.501	1.994	
CONTROL	SZT	-.746	.613	.232	-1.994	.501	

Based on estimated marginal means	
a. Adjustment for multiple comparisons: Bonferroni.	


Univariate Tests	
Measure:   MEASURE_1  	
	Sum of Squares	df	Mean Square	F	Sig.	Partial Eta Squared	Noncent. Parameter	
Contrast	4.679	1	4.679	1.482	.232	.043	1.482	
Error	104.197	33	3.157					

Univariate Tests	
Measure:   MEASURE_1  	
	Observed Powera	
Contrast	.219	
Error		

The F tests the effect of FINALGROUPS. This test is based on the linearly independent pairwise comparisons among the estimated marginal means.	
a. Computed using alpha = .05	

3. RegionofInterest

Estimates	
Measure:   MEASURE_1  	
RegionofInterest	Mean	Std. Error	95% Confidence Interval	
			Lower Bound	Upper Bound	
1	4.384	.645	3.071	5.697	
2	.106	.753	-1.426	1.637	
3	-.163	.848	-1.889	1.563	

Pairwise Comparisons	
Measure:   MEASURE_1  	
(I) RegionofInterest	(J) RegionofInterest	Mean Difference (I-J)	Std. Error	Sig.b	95% Confidence Interval for Differenceb	
					Lower Bound	Upper Bound	
1	2	4.278*	1.268	.006	1.080	7.477	
	3	4.547*	1.307	.004	1.249	7.844	
2	1	-4.278*	1.268	.006	-7.477	-1.080	
	3	.268	.972	1.000	-2.184	2.721	
3	1	-4.547*	1.307	.004	-7.844	-1.249	
	2	-.268	.972	1.000	-2.721	2.184	

Based on estimated marginal means	
*. The mean difference is significant at the .05 level.	
b. Adjustment for multiple comparisons: Bonferroni.	

Multivariate Tests	
	Value	F	Hypothesis df	Error df	Sig.	Partial Eta Squared	Noncent. Parameter	
Pillai's trace	.293	6.644a	2.000	32.000	.004	.293	13.287	
Wilks' lambda	.707	6.644a	2.000	32.000	.004	.293	13.287	
Hotelling's trace	.415	6.644a	2.000	32.000	.004	.293	13.287	
Roy's largest root	.415	6.644a	2.000	32.000	.004	.293	13.287	

Multivariate Tests	
	Observed Powerb	
Pillai's trace	.885	
Wilks' lambda	.885	
Hotelling's trace	.885	
Roy's largest root	.885	

Each F tests the multivariate effect of RegionofInterest. These tests are based on the linearly independent pairwise comparisons among the estimated marginal means.	
a. Exact statistic	
b. Computed using alpha = .05	

4. PairedTone

Estimates	
Measure:   MEASURE_1  	
PairedTone	Mean	Std. Error	95% Confidence Interval	
			Lower Bound	Upper Bound	
1	1.674	.390	.880	2.468	
2	1.210	.382	.433	1.988	


Pairwise Comparisons	
Measure:   MEASURE_1  	
(I) PairedTone	(J) PairedTone	Mean Difference (I-J)	Std. Error	Sig.a	95% Confidence Interval for Differencea	
					Lower Bound	Upper Bound	
1	2	.464	.470	.331	-.492	1.419	
2	1	-.464	.470	.331	-1.419	.492	

Based on estimated marginal means	
a. Adjustment for multiple comparisons: Bonferroni.	

Multivariate Tests	
	Value	F	Hypothesis df	Error df	Sig.	Partial Eta Squared	Noncent. Parameter	
Pillai's trace	.029	.974a	1.000	33.000	.331	.029	.974	
Wilks' lambda	.971	.974a	1.000	33.000	.331	.029	.974	
Hotelling's trace	.030	.974a	1.000	33.000	.331	.029	.974	
Roy's largest root	.030	.974a	1.000	33.000	.331	.029	.974	

Multivariate Tests	
	Observed Powerb	
Pillai's trace	.160	
Wilks' lambda	.160	
Hotelling's trace	.160	
Roy's largest root	.160	

Each F tests the multivariate effect of PairedTone. These tests are based on the linearly independent pairwise comparisons among the estimated marginal means.	
a. Exact statistic	
b. Computed using alpha = .05	

5. FINALGROUPS * RegionofInterest	
Measure:   MEASURE_1  	
FINALGROUPS	RegionofInterest	Mean	Std. Error	95% Confidence Interval	
				Lower Bound	Upper Bound	
SZT	1	4.568	1.000	2.534	6.601	
	2	1.215	1.166	-1.158	3.588	
	3	-.337	1.314	-3.010	2.337	
CONTROL	1	4.200	.816	2.540	5.861	
	2	-1.004	.952	-2.942	.934	
	3	.011	1.073	-2.172	2.194	

6. FINALGROUPS * PairedTone	
Measure:   MEASURE_1  	
FINALGROUPS	PairedTone	Mean	Std. Error	95% Confidence Interval	
				Lower Bound	Upper Bound	
SZT	1	2.324	.604	1.094	3.553	
	2	1.307	.592	.103	2.512	
CONTROL	1	1.024	.493	.020	2.028	
	2	1.114	.483	.130	2.097	

7. RegionofInterest * PairedTone	
Measure:   MEASURE_1  	
RegionofInterest	PairedTone	Mean	Std. Error	95% Confidence Interval	
				Lower Bound	Upper Bound	
1	1	5.534	.774	3.959	7.109	
	2	3.233	.973	1.255	5.212	
2	1	.062	.890	-1.750	1.873	
	2	.149	.850	-1.579	1.878	
3	1	-.574	1.117	-2.848	1.699	
	2	.249	1.164	-2.120	2.617	

8. FINALGROUPS * RegionofInterest * PairedTone	
Measure:   MEASURE_1  	
FINALGROUPS	RegionofInterest	PairedTone	Mean	Std. Error	95% Confidence Interval	
					Lower Bound	Upper Bound	
SZT	1	1	5.520	1.199	3.080	7.960	
		2	3.615	1.507	.550	6.680	
	2	1	1.687	1.379	-1.119	4.494	
		2	.743	1.316	-1.935	3.421	
	3	1	-.236	1.731	-3.759	3.286	
		2	-.437	1.803	-4.106	3.232	
CONTROL	1	1	5.548	.979	3.556	7.541	
		2	2.852	1.230	.349	5.355	
	2	1	-1.564	1.126	-3.855	.728	
		2	-.444	1.075	-2.631	1.742	
	3	1	-.912	1.414	-3.788	1.964	
		2	.934	1.472	-2.062	3.930	


T-Test - Paired-samples t-test exploring the maximum amplitude difference in the central region for the infant cohort.

Paired Samples Statistics	
	Mean	N	Std. Deviation	Std. Error Mean	
Pair 1	BEEP1_MAXAMP_CENTRAL	5.5372	35	4.42064	.74722	
	BEEP2_MAXAMP_CENTRAL	3.1571	35	5.56679	.94096	

Paired Samples Correlations	
	N	Correlation	Sig.	
Pair 1	BEEP1_MAXAMP_CENTRAL & BEEP2_MAXAMP_CENTRAL	35	.080	.649	

Paired Samples Test	
	Paired Differences	t	
	Mean	Std. Deviation	Std. Error Mean	95% Confidence Interval of the Difference		
				Lower	Upper		
Pair 1	BEEP1_MAXAMP_CENTRAL - BEEP2_MAXAMP_CENTRAL	2.38009	6.82734	1.15403	.03481	4.72536	2.062	

Paired Samples Test	
	df	Sig. (2-tailed)	
			
			
Pair 1	BEEP1_MAXAMP_CENTRAL - BEEP2_MAXAMP_CENTRAL	34	.047	


T-Test  - Paired-samples t-test exploring the mean amplitude difference in the central region for the 
    infant cohort.
  

Paired Samples Statistics	
	Mean	N	Std. Deviation	Std. Error Mean	
Pair 1	BEEP1_MEANAMP_CENTRAL	2.2240	35	4.08379	.69029	
	BEEP2_MEANAMP_CENTRAL	1.1208	35	5.74355	.97084	

Paired Samples Correlations	
	N	Correlation	Sig.	
Pair 1	BEEP1_MEANAMP_CENTRAL & BEEP2_MEANAMP_CENTRAL	35	-.009	.961	

Paired Samples Test	
	Paired Differences	t	
	Mean	Std. Deviation	Std. Error Mean	95% Confidence Interval of the Difference		
				Lower	Upper		
Pair 1	BEEP1_MEANAMP_CENTRAL - BEEP2_MEANAMP_CENTRAL	1.10321	7.07608	1.19608	-1.32751	3.53393	.922	

Paired Samples Test	
	df	Sig. (2-tailed)	
			
			
Pair 1	BEEP1_MEANAMP_CENTRAL - BEEP2_MEANAMP_CENTRAL	34	.363	

Correlations - Pearson Correlations between the mothers' sO-LIFE total & dimension scores and the infants' P50 amplitude differences and suppression ratios.

Correlations	
	OLIFE_TOTAL	OLIFE_UE	OLIFE_CD	OLIFE_IA	OLIFE_IN	MEANAMP_LEFTtemporal_DIFFERENCE	meanamp_lefttemporal_suppressioneffect	
OLIFE_TOTAL	Pearson Correlation	1	.863**	.913**	.760**	.739**	.177	.197	
	Sig. (2-tailed)		.000	.000	.000	.000	.309	.257	
	N	35	35	35	35	35	35	35	
OLIFE_UE	Pearson Correlation	.863**	1	.766**	.440**	.547**	.242	.261	
	Sig. (2-tailed)	.000		.000	.008	.001	.161	.131	
	N	35	35	35	35	35	35	35	
OLIFE_CD	Pearson Correlation	.913**	.766**	1	.703**	.465**	.160	.196	
	Sig. (2-tailed)	.000	.000		.000	.005	.359	.259	
	N	35	35	35	35	35	35	35	
OLIFE_IA	Pearson Correlation	.760**	.440**	.703**	1	.468**	.161	.197	
	Sig. (2-tailed)	.000	.008	.000		.005	.356	.256	
	N	35	35	35	35	35	35	35	
OLIFE_IN	Pearson Correlation	.739**	.547**	.465**	.468**	1	.013	-.013	
	Sig. (2-tailed)	.000	.001	.005	.005		.942	.940	
	N	35	35	35	35	35	35	35	
MEANAMP_LEFTtemporal_DIFFERENCE	Pearson Correlation	.177	.242	.160	.161	.013	1	.040	
	Sig. (2-tailed)	.309	.161	.359	.356	.942		.819	
	N	35	35	35	35	35	35	35	
meanamp_lefttemporal_suppressioneffect	Pearson Correlation	.197	.261	.196	.197	-.013	.040	1	
	Sig. (2-tailed)	.257	.131	.259	.256	.940	.819		
	N	35	35	35	35	35	35	35	
MEANAMP_RIGHTtemporal_DIFFERENCE	Pearson Correlation	.187	.297	.156	.121	.020	.441**	.127	
	Sig. (2-tailed)	.283	.083	.370	.487	.907	.008	.467	
	N	35	35	35	35	35	35	35	
meanamp_righttemporal_suppressioneffect	Pearson Correlation	-.345*	-.409*	-.361*	-.137	-.164	-.047	.008	
	Sig. (2-tailed)	.042	.015	.033	.434	.347	.787	.965	
	N	35	35	35	35	35	35	35	
MEANAMP_CENTRAL_DIFFERENCE	Pearson Correlation	.003	-.124	.050	-.049	.117	-.523**	-.084	
	Sig. (2-tailed)	.988	.478	.776	.780	.504	.001	.632	
	N	35	35	35	35	35	35	35	
meanamp_central_suppressioneffect	Pearson Correlation	.225	.275	.162	.314	.025	.269	.032	
	Sig. (2-tailed)	.195	.110	.352	.066	.885	.118	.856	
	N	35	35	35	35	35	35	35	
maxampl_central_difference	Pearson Correlation	-.065	-.127	-.016	-.142	.041	-.566**	-.019	
	Sig. (2-tailed)	.709	.467	.927	.415	.813	.000	.913	
	N	35	35	35	35	35	35	35	
maxampl_lefttemporal_difference	Pearson Correlation	.052	.262	.021	-.071	-.084	.791**	.103	
	Sig. (2-tailed)	.767	.128	.905	.683	.633	.000	.558	
	N	35	35	35	35	35	35	35	
maxampl_righttemporal_difference	Pearson Correlation	.220	.320	.192	.156	.037	.411*	.086	
	Sig. (2-tailed)	.204	.061	.268	.371	.834	.014	.623	
	N	35	35	35	35	35	35	35	

Correlations	
	MEANAMP_RIGHTtemporal_DIFFERENCE	meanamp_righttemporal_suppressioneffect	MEANAMP_CENTRAL_DIFFERENCE	meanamp_central_suppressioneffect	maxampl_central_difference	maxampl_lefttemporal_difference	
OLIFE_TOTAL	Pearson Correlation	.187	-.345*	.003	.225	-.065	.052	
	Sig. (2-tailed)	.283	.042	.988	.195	.709	.767	
	N	35	35	35	35	35	35	
OLIFE_UE	Pearson Correlation	.297	-.409*	-.124	.275	-.127	.262	
	Sig. (2-tailed)	.083	.015	.478	.110	.467	.128	
	N	35	35	35	35	35	35	
OLIFE_CD	Pearson Correlation	.156	-.361*	.050	.162	-.016	.021	
	Sig. (2-tailed)	.370	.033	.776	.352	.927	.905	
	N	35	35	35	35	35	35	
OLIFE_IA	Pearson Correlation	.121	-.137	-.049	.314	-.142	-.071	
	Sig. (2-tailed)	.487	.434	.780	.066	.415	.683	
	N	35	35	35	35	35	35	
OLIFE_IN	Pearson Correlation	.020	-.164	.117	.025	.041	-.084	
	Sig. (2-tailed)	.907	.347	.504	.885	.813	.633	
	N	35	35	35	35	35	35	
MEANAMP_LEFTtemporal_DIFFERENCE	Pearson Correlation	.441**	-.047	-.523**	.269	-.566**	.791**	
	Sig. (2-tailed)	.008	.787	.001	.118	.000	.000	
	N	35	35	35	35	35	35	
meanamp_lefttemporal_suppressioneffect	Pearson Correlation	.127	.008	-.084	.032	-.019	.103	
	Sig. (2-tailed)	.467	.965	.632	.856	.913	.558	
	N	35	35	35	35	35	35	
MEANAMP_RIGHTtemporal_DIFFERENCE	Pearson Correlation	1	.319	-.692**	.232	-.687**	.352*	
	Sig. (2-tailed)		.062	.000	.181	.000	.038	
	N	35	35	35	35	35	35	
meanamp_righttemporal_suppressioneffect	Pearson Correlation	.319	1	-.206	-.227	-.164	-.151	
	Sig. (2-tailed)	.062		.235	.190	.347	.386	
	N	35	35	35	35	35	35	
MEANAMP_CENTRAL_DIFFERENCE	Pearson Correlation	-.692**	-.206	1	-.444**	.967**	-.505**	
	Sig. (2-tailed)	.000	.235		.007	.000	.002	
	N	35	35	35	35	35	35	
meanamp_central_suppressioneffect	Pearson Correlation	.232	-.227	-.444**	1	-.403*	.403*	
	Sig. (2-tailed)	.181	.190	.007		.016	.016	
	N	35	35	35	35	35	35	
maxampl_central_difference	Pearson Correlation	-.687**	-.164	.967**	-.403*	1	-.452**	
	Sig. (2-tailed)	.000	.347	.000	.016		.006	
	N	35	35	35	35	35	35	
maxampl_lefttemporal_difference	Pearson Correlation	.352*	-.151	-.505**	.403*	-.452**	1	
	Sig. (2-tailed)	.038	.386	.002	.016	.006		
	N	35	35	35	35	35	35	
maxampl_righttemporal_difference	Pearson Correlation	.967**	.298	-.659**	.233	-.652**	.308	
	Sig. (2-tailed)	.000	.082	.000	.178	.000	.072	
	N	35	35	35	35	35	35	

Correlations	
	maxampl_righttemporal_difference	
OLIFE_TOTAL	Pearson Correlation	.220	
	Sig. (2-tailed)	.204	
	N	35	
OLIFE_UE	Pearson Correlation	.320	
	Sig. (2-tailed)	.061	
	N	35	
OLIFE_CD	Pearson Correlation	.192	
	Sig. (2-tailed)	.268	
	N	35	
OLIFE_IA	Pearson Correlation	.156	
	Sig. (2-tailed)	.371	
	N	35	
OLIFE_IN	Pearson Correlation	.037	
	Sig. (2-tailed)	.834	
	N	35	
MEANAMP_LEFTtemporal_DIFFERENCE	Pearson Correlation	.411*	
	Sig. (2-tailed)	.014	
	N	35	
meanamp_lefttemporal_suppressioneffect	Pearson Correlation	.086	
	Sig. (2-tailed)	.623	
	N	35	
MEANAMP_RIGHTtemporal_DIFFERENCE	Pearson Correlation	.967**	
	Sig. (2-tailed)	.000	
	N	35	
meanamp_righttemporal_suppressioneffect	Pearson Correlation	.298	
	Sig. (2-tailed)	.082	
	N	35	
MEANAMP_CENTRAL_DIFFERENCE	Pearson Correlation	-.659**	
	Sig. (2-tailed)	.000	
	N	35	
meanamp_central_suppressioneffect	Pearson Correlation	.233	
	Sig. (2-tailed)	.178	
	N	35	
maxampl_central_difference	Pearson Correlation	-.652**	
	Sig. (2-tailed)	.000	
	N	35	
maxampl_lefttemporal_difference	Pearson Correlation	.308	
	Sig. (2-tailed)	.072	
	N	35	
maxampl_righttemporal_difference	Pearson Correlation	1	
	Sig. (2-tailed)		
	N	35	

**. Correlation is significant at the 0.01 level (2-tailed).	
*. Correlation is significant at the 0.05 level (2-tailed).	


General Linear Model - 2 (groups) x 3 (region of interest) x 2 (pairedtone) RM ANOVA to explore the maternal P50 mean amplitudes.

Within-Subjects Factors	
Measure:   MEASURE_1  	
RegionofInterest	PairedTone	Dependent Variable	
1	1	BEEP1_MEANAMP_lefttemporal	
	2	BEEP2_MEANAMP_lefttemporal	
2	1	BEEP1_MeanAmp_rightemporal	
	2	BEEP2_MeanAmp_righttemporal	
3	1	beep1_meanamp_central	
	2	beep2_meanamp_central	

Between-Subjects Factors	
	Value Label	N	
finalgroups	1.00	szt	23	
	2.00	control	30	

Descriptive Statistics	
	finalgroups	Mean	Std. Deviation	N	
BEEP1_MEANAMP_lefttemporal	szt	2.7620	1.69528	23	
	control	3.0336	1.57914	30	
	Total	2.9157	1.62021	53	
BEEP2_MEANAMP_lefttemporal	szt	2.6844	2.45118	23	
	control	1.8045	2.29816	30	
	Total	2.1863	2.38355	53	
BEEP1_MeanAmp_rightemporal	szt	2.5226	1.80713	23	
	control	3.2246	2.74129	30	
	Total	2.9200	2.38661	53	
BEEP2_MeanAmp_righttemporal	szt	3.0999	2.12575	23	
	control	2.7876	1.99611	30	
	Total	2.9231	2.03920	53	
beep1_meanamp_central	szt	-3.3668	1.94423	23	
	control	-3.2225	1.44626	30	
	Total	-3.2851	1.66462	53	
beep2_meanamp_central	szt	-2.2028	1.69364	23	
	control	-1.7081	1.14399	30	
	Total	-1.9228	1.41587	53	

Box's Test of Equality of Covariance Matricesa	
Box's M	27.023	
F	1.119	
df1	21	
df2	8229.753	
Sig.	.318	

Tests the null hypothesis that the observed covariance matrices of the dependent variables are equal across groups.a	
a. Design: Intercept + finalgroups 
 Within Subjects Design: RegionofInterest + PairedTone + RegionofInterest * PairedTone	

Multivariate Testsa	
Effect	Value	F	Hypothesis df	Error df	Sig.	
RegionofInterest	Pillai's Trace	.809	105.557b	2.000	50.000	.000	
	Wilks' Lambda	.191	105.557b	2.000	50.000	.000	
	Hotelling's Trace	4.222	105.557b	2.000	50.000	.000	
	Roy's Largest Root	4.222	105.557b	2.000	50.000	.000	
RegionofInterest * finalgroups	Pillai's Trace	.023	.585b	2.000	50.000	.561	
	Wilks' Lambda	.977	.585b	2.000	50.000	.561	
	Hotelling's Trace	.023	.585b	2.000	50.000	.561	
	Roy's Largest Root	.023	.585b	2.000	50.000	.561	
PairedTone	Pillai's Trace	.077	4.280b	1.000	51.000	.044	
	Wilks' Lambda	.923	4.280b	1.000	51.000	.044	
	Hotelling's Trace	.084	4.280b	1.000	51.000	.044	
	Roy's Largest Root	.084	4.280b	1.000	51.000	.044	
PairedTone * finalgroups	Pillai's Trace	.108	6.171b	1.000	51.000	.016	
	Wilks' Lambda	.892	6.171b	1.000	51.000	.016	
	Hotelling's Trace	.121	6.171b	1.000	51.000	.016	
	Roy's Largest Root	.121	6.171b	1.000	51.000	.016	
RegionofInterest * PairedTone	Pillai's Trace	.383	15.496b	2.000	50.000	.000	
	Wilks' Lambda	.617	15.496b	2.000	50.000	.000	
	Hotelling's Trace	.620	15.496b	2.000	50.000	.000	
	Roy's Largest Root	.620	15.496b	2.000	50.000	.000	
RegionofInterest * PairedTone * finalgroups	Pillai's Trace	.094	2.586b	2.000	50.000	.085	
	Wilks' Lambda	.906	2.586b	2.000	50.000	.085	
	Hotelling's Trace	.103	2.586b	2.000	50.000	.085	
	Roy's Largest Root	.103	2.586b	2.000	50.000	.085	

Multivariate Testsa	
Effect	Partial Eta Squared	Noncent. Parameter	Observed Powerc	
RegionofInterest	Pillai's Trace	.809	211.113	1.000	
	Wilks' Lambda	.809	211.113	1.000	
	Hotelling's Trace	.809	211.113	1.000	
	Roy's Largest Root	.809	211.113	1.000	
RegionofInterest * finalgroups	Pillai's Trace	.023	1.171	.142	
	Wilks' Lambda	.023	1.171	.142	
	Hotelling's Trace	.023	1.171	.142	
	Roy's Largest Root	.023	1.171	.142	
PairedTone	Pillai's Trace	.077	4.280	.528	
	Wilks' Lambda	.077	4.280	.528	
	Hotelling's Trace	.077	4.280	.528	
	Roy's Largest Root	.077	4.280	.528	
PairedTone * finalgroups	Pillai's Trace	.108	6.171	.683	
	Wilks' Lambda	.108	6.171	.683	
	Hotelling's Trace	.108	6.171	.683	
	Roy's Largest Root	.108	6.171	.683	
RegionofInterest * PairedTone	Pillai's Trace	.383	30.992	.999	
	Wilks' Lambda	.383	30.992	.999	
	Hotelling's Trace	.383	30.992	.999	
	Roy's Largest Root	.383	30.992	.999	
RegionofInterest * PairedTone * finalgroups	Pillai's Trace	.094	5.173	.493	
	Wilks' Lambda	.094	5.173	.493	
	Hotelling's Trace	.094	5.173	.493	
	Roy's Largest Root	.094	5.173	.493	

a. Design: Intercept + finalgroups 
 Within Subjects Design: RegionofInterest + PairedTone + RegionofInterest * PairedTone	
b. Exact statistic	
c. Computed using alpha = .05	

Mauchly's Test of Sphericitya	
Measure:   MEASURE_1  	
Within Subjects Effect	Mauchly's W	Approx. Chi-Square	df	Sig.	Epsilonb	
					Greenhouse-Geisser	Huynh-Feldt	
RegionofInterest	.833	9.150	2	.010	.857	.901	
PairedTone	1.000	.000	0	.	1.000	1.000	
RegionofInterest * PairedTone	.937	3.274	2	.195	.940	.994	

Mauchly's Test of Sphericitya	
Measure:   MEASURE_1  	
Within Subjects Effect	Epsilon	
	Lower-bound	
RegionofInterest	.500	
PairedTone	1.000	
RegionofInterest * PairedTone	.500	

Tests the null hypothesis that the error covariance matrix of the orthonormalized transformed dependent variables is proportional to an identity matrix.a	
a. Design: Intercept + finalgroups 
 Within Subjects Design: RegionofInterest + PairedTone + RegionofInterest * PairedTone	
b. May be used to adjust the degrees of freedom for the averaged tests of significance. Corrected tests are displayed in the Tests of Within-Subjects Effects table.	

Tests of Within-Subjects Effects	
Measure:   MEASURE_1  	
Source	Type III Sum of Squares	df	Mean Square	F	Sig.	
RegionofInterest	Sphericity Assumed	2004.442	2	1002.221	150.055	.000	
	Greenhouse-Geisser	2004.442	1.713	1169.820	150.055	.000	
	Huynh-Feldt	2004.442	1.802	1112.354	150.055	.000	
	Lower-bound	2004.442	1.000	2004.442	150.055	.000	
RegionofInterest * finalgroups	Sphericity Assumed	5.673	2	2.837	.425	.655	
	Greenhouse-Geisser	5.673	1.713	3.311	.425	.624	
	Huynh-Feldt	5.673	1.802	3.148	.425	.634	
	Lower-bound	5.673	1.000	5.673	.425	.518	
Error(RegionofInterest)	Sphericity Assumed	681.262	102	6.679			
	Greenhouse-Geisser	681.262	87.387	7.796			
	Huynh-Feldt	681.262	91.901	7.413			
	Lower-bound	681.262	51.000	13.358			
PairedTone	Sphericity Assumed	4.960	1	4.960	4.280	.044	
	Greenhouse-Geisser	4.960	1.000	4.960	4.280	.044	
	Huynh-Feldt	4.960	1.000	4.960	4.280	.044	
	Lower-bound	4.960	1.000	4.960	4.280	.044	
PairedTone * finalgroups	Sphericity Assumed	7.151	1	7.151	6.171	.016	
	Greenhouse-Geisser	7.151	1.000	7.151	6.171	.016	
	Huynh-Feldt	7.151	1.000	7.151	6.171	.016	
	Lower-bound	7.151	1.000	7.151	6.171	.016	
Error(PairedTone)	Sphericity Assumed	59.105	51	1.159			
	Greenhouse-Geisser	59.105	51.000	1.159			
	Huynh-Feldt	59.105	51.000	1.159			
	Lower-bound	59.105	51.000	1.159			
RegionofInterest * PairedTone	Sphericity Assumed	52.981	2	26.491	11.854	.000	
	Greenhouse-Geisser	52.981	1.881	28.170	11.854	.000	
	Huynh-Feldt	52.981	1.989	26.642	11.854	.000	
	Lower-bound	52.981	1.000	52.981	11.854	.001	
RegionofInterest * PairedTone * finalgroups	Sphericity Assumed	8.977	2	4.488	2.008	.139	
	Greenhouse-Geisser	8.977	1.881	4.773	2.008	.143	
	Huynh-Feldt	8.977	1.989	4.514	2.008	.140	
	Lower-bound	8.977	1.000	8.977	2.008	.163	
Error(RegionofInterest*PairedTone)	Sphericity Assumed	227.941	102	2.235			
	Greenhouse-Geisser	227.941	95.920	2.376			
	Huynh-Feldt	227.941	101.422	2.247			
	Lower-bound	227.941	51.000	4.469			

Tests of Within-Subjects Effects	
Measure:   MEASURE_1  	
Source	Partial Eta Squared	Noncent. Parameter	Observed Powera	
RegionofInterest	Sphericity Assumed	.746	300.109	1.000	
	Greenhouse-Geisser	.746	257.113	1.000	
	Huynh-Feldt	.746	270.396	1.000	
	Lower-bound	.746	150.055	1.000	
RegionofInterest * finalgroups	Sphericity Assumed	.008	.849	.117	
	Greenhouse-Geisser	.008	.728	.112	
	Huynh-Feldt	.008	.765	.114	
	Lower-bound	.008	.425	.098	
Error(RegionofInterest)	Sphericity Assumed				
	Greenhouse-Geisser				
	Huynh-Feldt				
	Lower-bound				
PairedTone	Sphericity Assumed	.077	4.280	.528	
	Greenhouse-Geisser	.077	4.280	.528	
	Huynh-Feldt	.077	4.280	.528	
	Lower-bound	.077	4.280	.528	
PairedTone * finalgroups	Sphericity Assumed	.108	6.171	.683	
	Greenhouse-Geisser	.108	6.171	.683	
	Huynh-Feldt	.108	6.171	.683	
	Lower-bound	.108	6.171	.683	
Error(PairedTone)	Sphericity Assumed				
	Greenhouse-Geisser				
	Huynh-Feldt				
	Lower-bound				
RegionofInterest * PairedTone	Sphericity Assumed	.189	23.708	.994	
	Greenhouse-Geisser	.189	22.295	.991	
	Huynh-Feldt	.189	23.574	.994	
	Lower-bound	.189	11.854	.922	
RegionofInterest * PairedTone * finalgroups	Sphericity Assumed	.038	4.017	.406	
	Greenhouse-Geisser	.038	3.778	.393	
	Huynh-Feldt	.038	3.994	.405	
	Lower-bound	.038	2.008	.285	
Error(RegionofInterest*PairedTone)	Sphericity Assumed				
	Greenhouse-Geisser				
	Huynh-Feldt				
	Lower-bound				

a. Computed using alpha = .05	

Tests of Within-Subjects Contrasts	
Measure:   MEASURE_1  	
Source	RegionofInterest	PairedTone	Type III Sum of Squares	df	Mean Square	F	
RegionofInterest	Linear		1406.058	1	1406.058	192.103	
	Quadratic		598.384	1	598.384	99.090	
RegionofInterest * finalgroups	Linear		5.065	1	5.065	.692	
	Quadratic		.608	1	.608	.101	
Error(RegionofInterest)	Linear		373.285	51	7.319		
	Quadratic		307.978	51	6.039		
PairedTone		Linear	4.960	1	4.960	4.280	
PairedTone * finalgroups		Linear	7.151	1	7.151	6.171	
Error(PairedTone)		Linear	59.105	51	1.159		
RegionofInterest * PairedTone	Linear	Linear	51.689	1	51.689	29.845	
	Quadratic	Linear	1.292	1	1.292	.472	
RegionofInterest * PairedTone * finalgroups	Linear	Linear	7.342	1	7.342	4.239	
	Quadratic	Linear	1.635	1	1.635	.597	
Error(RegionofInterest*PairedTone)	Linear	Linear	88.327	51	1.732		
	Quadratic	Linear	139.614	51	2.738		

Tests of Within-Subjects Contrasts	
Measure:   MEASURE_1  	
Source	RegionofInterest	PairedTone	Sig.	Partial Eta Squared	Noncent. Parameter	Observed Powera	
RegionofInterest	Linear		.000	.790	192.103	1.000	
	Quadratic		.000	.660	99.090	1.000	
RegionofInterest * finalgroups	Linear		.409	.013	.692	.129	
	Quadratic		.752	.002	.101	.061	
Error(RegionofInterest)	Linear						
	Quadratic						
PairedTone		Linear	.044	.077	4.280	.528	
PairedTone * finalgroups		Linear	.016	.108	6.171	.683	
Error(PairedTone)		Linear					
RegionofInterest * PairedTone	Linear	Linear	.000	.369	29.845	1.000	
	Quadratic	Linear	.495	.009	.472	.103	
RegionofInterest * PairedTone * finalgroups	Linear	Linear	.045	.077	4.239	.524	
	Quadratic	Linear	.443	.012	.597	.118	
Error(RegionofInterest*PairedTone)	Linear	Linear					
	Quadratic	Linear					

a. Computed using alpha = .05	

Levene's Test of Equality of Error Variancesa	
	F	df1	df2	Sig.	
BEEP1_MEANAMP_lefttemporal	.398	1	51	.531	
BEEP2_MEANAMP_lefttemporal	.374	1	51	.544	
BEEP1_MeanAmp_rightemporal	2.492	1	51	.121	
BEEP2_MeanAmp_righttemporal	.474	1	51	.494	
beep1_meanamp_central	.285	1	51	.596	
beep2_meanamp_central	2.336	1	51	.133	

Tests the null hypothesis that the error variance of the dependent variable is equal across groups.a	
a. Design: Intercept + finalgroups 
 Within Subjects Design: RegionofInterest + PairedTone + RegionofInterest * PairedTone	

Tests of Between-Subjects Effects	
Measure:   MEASURE_1  	
Transformed Variable:   Average  	
Source	Type III Sum of Squares	df	Mean Square	F	Sig.	Partial Eta Squared	Noncent. Parameter	
Intercept	282.926	1	282.926	71.363	.000	.583	71.363	
finalgroups	.383	1	.383	.097	.757	.002	.097	
Error	202.194	51	3.965					

Tests of Between-Subjects Effects	
Measure:   MEASURE_1  	
Transformed Variable:   Average  	
Source	Observed Powera	
Intercept	1.000	
finalgroups	.061	
Error		

a. Computed using alpha = .05	

Estimated Marginal Means

1. Grand Mean	
Measure:   MEASURE_1  	
Mean	Std. Error	95% Confidence Interval	
		Lower Bound	Upper Bound	
.952	.113	.725	1.178	

2. finalgroups

Estimates	
Measure:   MEASURE_1  	
finalgroups	Mean	Std. Error	95% Confidence Interval	
			Lower Bound	Upper Bound	
szt	.917	.169	.576	1.257	
control	.987	.148	.689	1.285	

Pairwise Comparisons	
Measure:   MEASURE_1  	
(I) finalgroups	(J) finalgroups	Mean Difference (I-J)	Std. Error	Sig.a	95% Confidence Interval for Differencea	
					Lower Bound	Upper Bound	
szt	control	-.070	.225	.757	-.522	.382	
control	szt	.070	.225	.757	-.382	.522	

Based on estimated marginal means	
a. Adjustment for multiple comparisons: Bonferroni.	


Univariate Tests	
Measure:   MEASURE_1  	
	Sum of Squares	df	Mean Square	F	Sig.	Partial Eta Squared	Noncent. Parameter	
Contrast	.064	1	.064	.097	.757	.002	.097	
Error	33.699	51	.661					

Univariate Tests	
Measure:   MEASURE_1  	
	Observed Powera	
Contrast	.061	
Error		

The F tests the effect of finalgroups. This test is based on the linearly independent pairwise comparisons among the estimated marginal means.	
a. Computed using alpha = .05	

3. RegionofInterest

Estimates	
Measure:   MEASURE_1  	
RegionofInterest	Mean	Std. Error	95% Confidence Interval	
			Lower Bound	Upper Bound	
1	2.571	.238	2.093	3.050	
2	2.909	.267	2.373	3.444	
3	-2.625	.196	-3.018	-2.232	

Pairwise Comparisons	
Measure:   MEASURE_1  	
(I) RegionofInterest	(J) RegionofInterest	Mean Difference (I-J)	Std. Error	Sig.b	95% Confidence Interval for Differenceb	
					Lower Bound	Upper Bound	
1	2	-.338	.279	.697	-1.029	.354	
	3	5.196*	.375	.000	4.268	6.124	
2	1	.338	.279	.697	-.354	1.029	
	3	5.534*	.408	.000	4.524	6.543	
3	1	-5.196*	.375	.000	-6.124	-4.268	
	2	-5.534*	.408	.000	-6.543	-4.524	

Based on estimated marginal means	
*. The mean difference is significant at the .05 level.	
b. Adjustment for multiple comparisons: Bonferroni.	

Multivariate Tests	
	Value	F	Hypothesis df	Error df	Sig.	Partial Eta Squared	Noncent. Parameter	
Pillai's trace	.809	105.557a	2.000	50.000	.000	.809	211.113	
Wilks' lambda	.191	105.557a	2.000	50.000	.000	.809	211.113	
Hotelling's trace	4.222	105.557a	2.000	50.000	.000	.809	211.113	
Roy's largest root	4.222	105.557a	2.000	50.000	.000	.809	211.113	

Multivariate Tests	
	Observed Powerb	
Pillai's trace	1.000	
Wilks' lambda	1.000	
Hotelling's trace	1.000	
Roy's largest root	1.000	

Each F tests the multivariate effect of RegionofInterest. These tests are based on the linearly independent pairwise comparisons among the estimated marginal means.	
a. Exact statistic	
b. Computed using alpha = .05	

4. PairedTone

Estimates	
Measure:   MEASURE_1  	
PairedTone	Mean	Std. Error	95% Confidence Interval	
			Lower Bound	Upper Bound	
1	.826	.114	.597	1.054	
2	1.078	.141	.795	1.360	

Pairwise Comparisons	
Measure:   MEASURE_1  	
(I) PairedTone	(J) PairedTone	Mean Difference (I-J)	Std. Error	Sig.b	95% Confidence Interval for Differenceb	
					Lower Bound	Upper Bound	
1	2	-.252*	.122	.044	-.497	-.007	
2	1	.252*	.122	.044	.007	.497	

Based on estimated marginal means	
*. The mean difference is significant at the .05 level.	
b. Adjustment for multiple comparisons: Bonferroni.	

Multivariate Tests	
	Value	F	Hypothesis df	Error df	Sig.	Partial Eta Squared	Noncent. Parameter	
Pillai's trace	.077	4.280a	1.000	51.000	.044	.077	4.280	
Wilks' lambda	.923	4.280a	1.000	51.000	.044	.077	4.280	
Hotelling's trace	.084	4.280a	1.000	51.000	.044	.077	4.280	
Roy's largest root	.084	4.280a	1.000	51.000	.044	.077	4.280	

Multivariate Tests	
	Observed Powerb	
Pillai's trace	.528	
Wilks' lambda	.528	
Hotelling's trace	.528	
Roy's largest root	.528	

Each F tests the multivariate effect of PairedTone. These tests are based on the linearly independent pairwise comparisons among the estimated marginal means.	
a. Exact statistic	
b. Computed using alpha = .05	

5. finalgroups * RegionofInterest	
Measure:   MEASURE_1  	
finalgroups	RegionofInterest	Mean	Std. Error	95% Confidence Interval	
				Lower Bound	Upper Bound	
szt	1	2.723	.359	2.003	3.443	
	2	2.811	.401	2.006	3.617	
	3	-2.785	.295	-3.376	-2.193	
control	1	2.419	.314	1.788	3.050	
	2	3.006	.351	2.301	3.712	
	3	-2.465	.258	-2.983	-1.947	

6. finalgroups * PairedTone	
Measure:   MEASURE_1  	
finalgroups	PairedTone	Mean	Std. Error	95% Confidence Interval	
				Lower Bound	Upper Bound	
szt	1	.639	.171	.295	.983	
	2	1.194	.212	.769	1.619	
control	1	1.012	.150	.711	1.313	
	2	.961	.186	.589	1.334	

7. RegionofInterest * PairedTone	
Measure:   MEASURE_1  	
RegionofInterest	PairedTone	Mean	Std. Error	95% Confidence Interval	
				Lower Bound	Upper Bound	
1	1	2.898	.226	2.444	3.351	
	2	2.244	.328	1.586	2.902	
2	1	2.874	.330	2.210	3.537	
	2	2.944	.284	2.373	3.515	
3	1	-3.295	.233	-3.762	-2.827	
	2	-1.955	.195	-2.347	-1.564	


8. finalgroups * RegionofInterest * PairedTone	
Measure:   MEASURE_1  	
finalgroups	RegionofInterest	PairedTone	Mean	Std. Error	95% Confidence Interval	
					Lower Bound	Upper Bound	
szt	1	1	2.762	.340	2.080	3.444	
		2	2.684	.493	1.694	3.675	
	2	1	2.523	.497	1.525	3.520	
		2	3.100	.428	2.241	3.959	
	3	1	-3.367	.350	-4.070	-2.664	
		2	-2.203	.294	-2.792	-1.614	
control	1	1	3.034	.298	2.436	3.631	
		2	1.804	.432	.937	2.671	
	2	1	3.225	.435	2.351	4.098	
		2	2.788	.375	2.035	3.540	
	3	1	-3.223	.307	-3.838	-2.607	
		2	-1.708	.257	-2.224	-1.192	

General Linear Model - a 2 (group) x 3 (region of interest) x 2 (paired-tone) RM ANOVA to explore the P50  maximum amplitudes in the maternal cohort.

Within-Subjects Factors	
Measure:   MEASURE_1  	
RegionofInterest	PairedTone	Dependent Variable	
1	1	beep1_maxampl_lefttemporal	
	2	beep2_maxampl_lefttemporal	
2	1	beep1_maxampl_righttemporal	
	2	beep2_maxampl_righttemporal	
3	1	beep1_maxampl_central	
	2	beep2_maxampl_central	

Between-Subjects Factors	
	Value Label	N	
finalgroups	1.00	szt	23	
	2.00	control	30	


Descriptive Statistics	
	finalgroups	Mean	Std. Deviation	N	
beep1_maxampl_lefttemporal	szt	3.9213	1.92084	23	
	control	4.2487	1.58786	30	
	Total	4.1066	1.73030	53	
beep2_maxampl_lefttemporal	szt	3.5760	2.54539	23	
	control	2.7658	2.20080	30	
	Total	3.1174	2.36784	53	
beep1_maxampl_righttemporal	szt	3.4536	1.99664	23	
	control	4.0277	2.85177	30	
	Total	3.7786	2.51090	53	
beep2_maxampl_righttemporal	szt	3.5980	2.49020	23	
	control	3.3696	2.69467	30	
	Total	3.4687	2.58576	53	
beep1_maxampl_central	szt	-1.1141	1.54380	23	
	control	-1.4534	1.24239	30	
	Total	-1.3061	1.37766	53	
beep2_maxampl_central	szt	-1.0285	1.44126	23	
	control	-.4191	1.06857	30	
	Total	-.6836	1.26831	53	

Box's Test of Equality of Covariance Matricesa	
Box's M	32.095	
F	1.329	
df1	21	
df2	8229.753	
Sig.	.143	

Tests the null hypothesis that the observed covariance matrices of the dependent variables are equal across groups.a	
a. Design: Intercept + finalgroups 
 Within Subjects Design: RegionofInterest + PairedTone + RegionofInterest * PairedTone	


Multivariate Testsa	
Effect	Value	F	Hypothesis df	Error df	Sig.	
RegionofInterest	Pillai's Trace	.796	97.495b	2.000	50.000	.000	
	Wilks' Lambda	.204	97.495b	2.000	50.000	.000	
	Hotelling's Trace	3.900	97.495b	2.000	50.000	.000	
	Roy's Largest Root	3.900	97.495b	2.000	50.000	.000	
RegionofInterest * finalgroups	Pillai's Trace	.012	.315b	2.000	50.000	.731	
	Wilks' Lambda	.988	.315b	2.000	50.000	.731	
	Hotelling's Trace	.013	.315b	2.000	50.000	.731	
	Roy's Largest Root	.013	.315b	2.000	50.000	.731	
PairedTone	Pillai's Trace	.038	2.021b	1.000	51.000	.161	
	Wilks' Lambda	.962	2.021b	1.000	51.000	.161	
	Hotelling's Trace	.040	2.021b	1.000	51.000	.161	
	Roy's Largest Root	.040	2.021b	1.000	51.000	.161	
PairedTone * finalgroups	Pillai's Trace	.025	1.330b	1.000	51.000	.254	
	Wilks' Lambda	.975	1.330b	1.000	51.000	.254	
	Hotelling's Trace	.026	1.330b	1.000	51.000	.254	
	Roy's Largest Root	.026	1.330b	1.000	51.000	.254	
RegionofInterest * PairedTone	Pillai's Trace	.240	7.889b	2.000	50.000	.001	
	Wilks' Lambda	.760	7.889b	2.000	50.000	.001	
	Hotelling's Trace	.316	7.889b	2.000	50.000	.001	
	Roy's Largest Root	.316	7.889b	2.000	50.000	.001	
RegionofInterest * PairedTone * finalgroups	Pillai's Trace	.152	4.477b	2.000	50.000	.016	
	Wilks' Lambda	.848	4.477b	2.000	50.000	.016	
	Hotelling's Trace	.179	4.477b	2.000	50.000	.016	
	Roy's Largest Root	.179	4.477b	2.000	50.000	.016	

Multivariate Testsa	
Effect	Partial Eta Squared	Noncent. Parameter	Observed Powerc	
RegionofInterest	Pillai's Trace	.796	194.989	1.000	
	Wilks' Lambda	.796	194.989	1.000	
	Hotelling's Trace	.796	194.989	1.000	
	Roy's Largest Root	.796	194.989	1.000	
RegionofInterest * finalgroups	Pillai's Trace	.012	.631	.097	
	Wilks' Lambda	.012	.631	.097	
	Hotelling's Trace	.012	.631	.097	
	Roy's Largest Root	.012	.631	.097	
PairedTone	Pillai's Trace	.038	2.021	.286	
	Wilks' Lambda	.038	2.021	.286	
	Hotelling's Trace	.038	2.021	.286	
	Roy's Largest Root	.038	2.021	.286	
PairedTone * finalgroups	Pillai's Trace	.025	1.330	.205	
	Wilks' Lambda	.025	1.330	.205	
	Hotelling's Trace	.025	1.330	.205	
	Roy's Largest Root	.025	1.330	.205	
RegionofInterest * PairedTone	Pillai's Trace	.240	15.779	.942	
	Wilks' Lambda	.240	15.779	.942	
	Hotelling's Trace	.240	15.779	.942	
	Roy's Largest Root	.240	15.779	.942	
RegionofInterest * PairedTone * finalgroups	Pillai's Trace	.152	8.955	.741	
	Wilks' Lambda	.152	8.955	.741	
	Hotelling's Trace	.152	8.955	.741	
	Roy's Largest Root	.152	8.955	.741	

a. Design: Intercept + finalgroups 
 Within Subjects Design: RegionofInterest + PairedTone + RegionofInterest * PairedTone	
b. Exact statistic	
c. Computed using alpha = .05	

Mauchly's Test of Sphericitya	
Measure:   MEASURE_1  	
Within Subjects Effect	Mauchly's W	Approx. Chi-Square	df	Sig.	Epsilonb	
					Greenhouse-Geisser	Huynh-Feldt	
RegionofInterest	.880	6.405	2	.041	.893	.941	
PairedTone	1.000	.000	0	.	1.000	1.000	
RegionofInterest * PairedTone	.957	2.179	2	.336	.959	1.000	

Mauchly's Test of Sphericitya	
Measure:   MEASURE_1  	
Within Subjects Effect	Epsilon	
	Lower-bound	
RegionofInterest	.500	
PairedTone	1.000	
RegionofInterest * PairedTone	.500	

Tests the null hypothesis that the error covariance matrix of the orthonormalized transformed dependent variables is proportional to an identity matrix.a	
a. Design: Intercept + finalgroups 
 Within Subjects Design: RegionofInterest + PairedTone + RegionofInterest * PairedTone	
b. May be used to adjust the degrees of freedom for the averaged tests of significance. Corrected tests are displayed in the Tests of Within-Subjects Effects table.	


Tests of Within-Subjects Effects	
Measure:   MEASURE_1  	
Source	Type III Sum of Squares	df	Mean Square	F	Sig.	
RegionofInterest	Sphericity Assumed	1484.529	2	742.265	114.278	.000	
	Greenhouse-Geisser	1484.529	1.785	831.515	114.278	.000	
	Huynh-Feldt	1484.529	1.882	788.799	114.278	.000	
	Lower-bound	1484.529	1.000	1484.529	114.278	.000	
RegionofInterest * finalgroups	Sphericity Assumed	2.732	2	1.366	.210	.811	
	Greenhouse-Geisser	2.732	1.785	1.530	.210	.786	
	Huynh-Feldt	2.732	1.882	1.452	.210	.798	
	Lower-bound	2.732	1.000	2.732	.210	.648	
Error(RegionofInterest)	Sphericity Assumed	662.514	102	6.495			
	Greenhouse-Geisser	662.514	91.052	7.276			
	Huynh-Feldt	662.514	95.983	6.902			
	Lower-bound	662.514	51.000	12.990			
PairedTone	Sphericity Assumed	3.241	1	3.241	2.021	.161	
	Greenhouse-Geisser	3.241	1.000	3.241	2.021	.161	
	Huynh-Feldt	3.241	1.000	3.241	2.021	.161	
	Lower-bound	3.241	1.000	3.241	2.021	.161	
PairedTone * finalgroups	Sphericity Assumed	2.133	1	2.133	1.330	.254	
	Greenhouse-Geisser	2.133	1.000	2.133	1.330	.254	
	Huynh-Feldt	2.133	1.000	2.133	1.330	.254	
	Lower-bound	2.133	1.000	2.133	1.330	.254	
Error(PairedTone)	Sphericity Assumed	81.764	51	1.603			
	Greenhouse-Geisser	81.764	51.000	1.603			
	Huynh-Feldt	81.764	51.000	1.603			
	Lower-bound	81.764	51.000	1.603			
RegionofInterest * PairedTone	Sphericity Assumed	28.395	2	14.198	6.403	.002	
	Greenhouse-Geisser	28.395	1.918	14.803	6.403	.003	
	Huynh-Feldt	28.395	2.000	14.198	6.403	.002	
	Lower-bound	28.395	1.000	28.395	6.403	.015	
RegionofInterest * PairedTone * finalgroups	Sphericity Assumed	16.341	2	8.171	3.685	.029	
	Greenhouse-Geisser	16.341	1.918	8.519	3.685	.030	
	Huynh-Feldt	16.341	2.000	8.171	3.685	.029	
	Lower-bound	16.341	1.000	16.341	3.685	.061	
Error(RegionofInterest*PairedTone)	Sphericity Assumed	226.177	102	2.217			
	Greenhouse-Geisser	226.177	97.828	2.312			
	Huynh-Feldt	226.177	102.000	2.217			
	Lower-bound	226.177	51.000	4.435			

Tests of Within-Subjects Effects	
Measure:   MEASURE_1  	
Source	Partial Eta Squared	Noncent. Parameter	Observed Powera	
RegionofInterest	Sphericity Assumed	.691	228.557	1.000	
	Greenhouse-Geisser	.691	204.024	1.000	
	Huynh-Feldt	.691	215.073	1.000	
	Lower-bound	.691	114.278	1.000	
RegionofInterest * finalgroups	Sphericity Assumed	.004	.421	.082	
	Greenhouse-Geisser	.004	.375	.080	
	Huynh-Feldt	.004	.396	.081	
	Lower-bound	.004	.210	.074	
Error(RegionofInterest)	Sphericity Assumed				
	Greenhouse-Geisser				
	Huynh-Feldt				
	Lower-bound				
PairedTone	Sphericity Assumed	.038	2.021	.286	
	Greenhouse-Geisser	.038	2.021	.286	
	Huynh-Feldt	.038	2.021	.286	
	Lower-bound	.038	2.021	.286	
PairedTone * finalgroups	Sphericity Assumed	.025	1.330	.205	
	Greenhouse-Geisser	.025	1.330	.205	
	Huynh-Feldt	.025	1.330	.205	
	Lower-bound	.025	1.330	.205	
Error(PairedTone)	Sphericity Assumed				
	Greenhouse-Geisser				
	Huynh-Feldt				
	Lower-bound				
RegionofInterest * PairedTone	Sphericity Assumed	.112	12.806	.895	
	Greenhouse-Geisser	.112	12.282	.885	
	Huynh-Feldt	.112	12.806	.895	
	Lower-bound	.112	6.403	.699	
RegionofInterest * PairedTone * finalgroups	Sphericity Assumed	.067	7.369	.666	
	Greenhouse-Geisser	.067	7.068	.652	
	Huynh-Feldt	.067	7.369	.666	
	Lower-bound	.067	3.685	.470	
Error(RegionofInterest*PairedTone)	Sphericity Assumed				
	Greenhouse-Geisser				
	Huynh-Feldt				
	Lower-bound				

a. Computed using alpha = .05	

Tests of Within-Subjects Contrasts	
Measure:   MEASURE_1  	
Source	RegionofInterest	PairedTone	Type III Sum of Squares	df	Mean Square	F	
RegionofInterest	Linear		1117.172	1	1117.172	191.557	
	Quadratic		367.357	1	367.357	51.318	
RegionofInterest * finalgroups	Linear		1.846	1	1.846	.316	
	Quadratic		.886	1	.886	.124	
Error(RegionofInterest)	Linear		297.434	51	5.832		
	Quadratic		365.080	51	7.158		
PairedTone		Linear	3.241	1	3.241	2.021	
PairedTone * finalgroups		Linear	2.133	1	2.133	1.330	
Error(PairedTone)		Linear	81.764	51	1.603		
RegionofInterest * PairedTone	Linear	Linear	28.285	1	28.285	15.886	
	Quadratic	Linear	.110	1	.110	.042	
RegionofInterest * PairedTone * finalgroups	Linear	Linear	14.165	1	14.165	7.956	
	Quadratic	Linear	2.176	1	2.176	.820	
Error(RegionofInterest*PairedTone)	Linear	Linear	90.804	51	1.780		
	Quadratic	Linear	135.373	51	2.654		

Tests of Within-Subjects Contrasts	
Measure:   MEASURE_1  	
Source	RegionofInterest	PairedTone	Sig.	Partial Eta Squared	Noncent. Parameter	Observed Powera	
RegionofInterest	Linear		.000	.790	191.557	1.000	
	Quadratic		.000	.502	51.318	1.000	
RegionofInterest * finalgroups	Linear		.576	.006	.316	.086	
	Quadratic		.726	.002	.124	.064	
Error(RegionofInterest)	Linear						
	Quadratic						
PairedTone		Linear	.161	.038	2.021	.286	
PairedTone * finalgroups		Linear	.254	.025	1.330	.205	
Error(PairedTone)		Linear					
RegionofInterest * PairedTone	Linear	Linear	.000	.238	15.886	.974	
	Quadratic	Linear	.839	.001	.042	.055	
RegionofInterest * PairedTone * finalgroups	Linear	Linear	.007	.135	7.956	.790	
	Quadratic	Linear	.370	.016	.820	.144	
Error(RegionofInterest*PairedTone)	Linear	Linear					
	Quadratic	Linear					

a. Computed using alpha = .05	

Levene's Test of Equality of Error Variancesa	
	F	df1	df2	Sig.	
beep1_maxampl_lefttemporal	1.675	1	51	.201	
beep2_maxampl_lefttemporal	.729	1	51	.397	
beep1_maxampl_righttemporal	2.092	1	51	.154	
beep2_maxampl_righttemporal	.000	1	51	.984	
beep1_maxampl_central	.103	1	51	.750	
beep2_maxampl_central	1.745	1	51	.192	

Tests the null hypothesis that the error variance of the dependent variable is equal across groups.a	
a. Design: Intercept + finalgroups 
 Within Subjects Design: RegionofInterest + PairedTone + RegionofInterest * PairedTone	

Tests of Between-Subjects Effects	
Measure:   MEASURE_1  	
Transformed Variable:   Average  	
Source	Type III Sum of Squares	df	Mean Square	F	Sig.	Partial Eta Squared	Noncent. Parameter	
Intercept	1350.226	1	1350.226	219.730	.000	.812	219.730	
finalgroups	.038	1	.038	.006	.937	.000	.006	
Error	313.391	51	6.145					

Tests of Between-Subjects Effects	
Measure:   MEASURE_1  	
Transformed Variable:   Average  	
Source	Observed Powera	
Intercept	1.000	
finalgroups	.051	
Error		

a. Computed using alpha = .05	

Estimated Marginal Means

1. Grand Mean	
Measure:   MEASURE_1  	
Mean	Std. Error	95% Confidence Interval	
		Lower Bound	Upper Bound	
2.079	.140	1.797	2.360	

2. finalgroups
Estimates	
Measure:   MEASURE_1  	
finalgroups	Mean	Std. Error	95% Confidence Interval	
			Lower Bound	Upper Bound	
szt	2.068	.211	1.644	2.491	
control	2.090	.185	1.719	2.461	

Pairwise Comparisons	
Measure:   MEASURE_1  	
(I) finalgroups	(J) finalgroups	Mean Difference (I-J)	Std. Error	Sig.a	95% Confidence Interval for Differencea	
					Lower Bound	Upper Bound	
szt	control	-.022	.280	.937	-.585	.541	
control	szt	.022	.280	.937	-.541	.585	

Based on estimated marginal means	
a. Adjustment for multiple comparisons: Bonferroni.	

Univariate Tests	
Measure:   MEASURE_1  	
	Sum of Squares	df	Mean Square	F	Sig.	Partial Eta Squared	Noncent. Parameter	
Contrast	.006	1	.006	.006	.937	.000	.006	
Error	52.232	51	1.024					

Univariate Tests	
Measure:   MEASURE_1  	
	Observed Powera	
Contrast	.051	
Error		

The F tests the effect of finalgroups. This test is based on the linearly independent pairwise comparisons among the estimated marginal means.	
a. Computed using alpha = .05	

3. RegionofInterest

Estimates	
Measure:   MEASURE_1  	
RegionofInterest	Mean	Std. Error	95% Confidence Interval	
			Lower Bound	Upper Bound	
1	3.628	.244	3.137	4.118	
2	3.612	.316	2.977	4.247	
3	-1.004	.155	-1.315	-.693	

Pairwise Comparisons	
Measure:   MEASURE_1  	
(I) RegionofInterest	(J) RegionofInterest	Mean Difference (I-J)	Std. Error	Sig.b	95% Confidence Interval for Differenceb	
					Lower Bound	Upper Bound	
1	2	.016	.309	1.000	-.749	.780	
	3	4.632*	.335	.000	3.803	5.460	
2	1	-.016	.309	1.000	-.780	.749	
	3	4.616*	.409	.000	3.605	5.627	
3	1	-4.632*	.335	.000	-5.460	-3.803	
	2	-4.616*	.409	.000	-5.627	-3.605	

Based on estimated marginal means	
*. The mean difference is significant at the .05 level.	
b. Adjustment for multiple comparisons: Bonferroni.	

Multivariate Tests	
	Value	F	Hypothesis df	Error df	Sig.	Partial Eta Squared	Noncent. Parameter	
Pillai's trace	.796	97.495a	2.000	50.000	.000	.796	194.989	
Wilks' lambda	.204	97.495a	2.000	50.000	.000	.796	194.989	
Hotelling's trace	3.900	97.495a	2.000	50.000	.000	.796	194.989	
Roy's largest root	3.900	97.495a	2.000	50.000	.000	.796	194.989	

Multivariate Tests	
	Observed Powerb	
Pillai's trace	1.000	
Wilks' lambda	1.000	
Hotelling's trace	1.000	
Roy's largest root	1.000	

Each F tests the multivariate effect of RegionofInterest. These tests are based on the linearly independent pairwise comparisons among the estimated marginal means.	
a. Exact statistic	
b. Computed using alpha = .05	

4. PairedTone

Estimates	
Measure:   MEASURE_1  	
PairedTone	Mean	Std. Error	95% Confidence Interval	
			Lower Bound	Upper Bound	
1	2.181	.141	1.897	2.464	
2	1.977	.172	1.631	2.323	

Pairwise Comparisons	
Measure:   MEASURE_1  	
(I) PairedTone	(J) PairedTone	Mean Difference (I-J)	Std. Error	Sig.a	95% Confidence Interval for Differencea	
					Lower Bound	Upper Bound	
1	2	.204	.143	.161	-.084	.491	
2	1	-.204	.143	.161	-.491	.084	

Based on estimated marginal means	
a. Adjustment for multiple comparisons: Bonferroni.	

Multivariate Tests	
	Value	F	Hypothesis df	Error df	Sig.	Partial Eta Squared	Noncent. Parameter	
Pillai's trace	.038	2.021a	1.000	51.000	.161	.038	2.021	
Wilks' lambda	.962	2.021a	1.000	51.000	.161	.038	2.021	
Hotelling's trace	.040	2.021a	1.000	51.000	.161	.038	2.021	
Roy's largest root	.040	2.021a	1.000	51.000	.161	.038	2.021	

Multivariate Tests	
	Observed Powerb	
Pillai's trace	.286	
Wilks' lambda	.286	
Hotelling's trace	.286	
Roy's largest root	.286	

Each F tests the multivariate effect of PairedTone. These tests are based on the linearly independent pairwise comparisons among the estimated marginal means.	
a. Exact statistic	
b. Computed using alpha = .05	

5. finalgroups * RegionofInterest	
Measure:   MEASURE_1  	
finalgroups	RegionofInterest	Mean	Std. Error	95% Confidence Interval	
				Lower Bound	Upper Bound	
szt	1	3.749	.368	3.011	4.487	
	2	3.526	.476	2.570	4.481	
	3	-1.071	.233	-1.539	-.604	
control	1	3.507	.322	2.861	4.153	
	2	3.699	.417	2.862	4.535	
	3	-.936	.204	-1.346	-.527	

6. finalgroups * PairedTone	
Measure:   MEASURE_1  	
finalgroups	PairedTone	Mean	Std. Error	95% Confidence Interval	
				Lower Bound	Upper Bound	
szt	1	2.087	.213	1.660	2.514	
	2	2.048	.259	1.528	2.569	
control	1	2.274	.186	1.901	2.648	
	2	1.905	.227	1.450	2.361	

7. RegionofInterest * PairedTone	
Measure:   MEASURE_1  	
RegionofInterest	PairedTone	Mean	Std. Error	95% Confidence Interval	
				Lower Bound	Upper Bound	
1	1	4.085	.241	3.601	4.569	
	2	3.171	.326	2.516	3.826	
2	1	3.741	.349	3.040	4.441	
	2	3.484	.361	2.758	4.209	
3	1	-1.284	.191	-1.668	-.900	
	2	-.724	.172	-1.070	-.378	

8. finalgroups * RegionofInterest * PairedTone	
Measure:   MEASURE_1  	
finalgroups	RegionofInterest	PairedTone	Mean	Std. Error	95% Confidence Interval	
					Lower Bound	Upper Bound	
szt	1	1	3.921	.363	3.193	4.649	
		2	3.576	.491	2.590	4.562	
	2	1	3.454	.525	2.399	4.508	
		2	3.598	.544	2.506	4.690	
	3	1	-1.114	.288	-1.692	-.536	
		2	-1.029	.259	-1.549	-.508	
control	1	1	4.249	.318	3.611	4.886	
		2	2.766	.430	1.902	3.629	
	2	1	4.028	.460	3.104	4.951	
		2	3.370	.476	2.413	4.326	
	3	1	-1.453	.252	-1.959	-.947	
		2	-.419	.227	-.875	.037	


T-Test  - Paired-samples t-test to explore the significant main effects and interactions in the maternal cohort.
Paired Samples Statistics	
	Mean	N	Std. Deviation	Std. Error Mean	
Pair 1	BEEP1_MEANAMP_lefttemporal	2.9157	53	1.62021	.22255	
	BEEP2_MEANAMP_lefttemporal	2.1863	53	2.38355	.32741	
Pair 2	BEEP1_MeanAmp_rightemporal	2.9200	53	2.38661	.32783	
	BEEP2_MeanAmp_righttemporal	2.9231	53	2.03920	.28011	
Pair 3	beep1_meanamp_central	-3.2851	53	1.66462	.22865	
	beep2_meanamp_central	-1.9228	53	1.41587	.19449	
Pair 4	beep1_maxampl_lefttemporal	4.1066	53	1.73030	.23767	
	beep2_maxampl_lefttemporal	3.1174	53	2.36784	.32525	
Pair 5	beep1_maxampl_righttemporal	3.7786	53	2.51090	.34490	
	beep2_maxampl_righttemporal	3.4687	53	2.58576	.35518	
Pair 6	beep1_maxampl_central	-1.3061	53	1.37766	.18924	
	beep2_maxampl_central	-.6836	53	1.26831	.17422	

Paired Samples Correlations	
	N	Correlation	Sig.	
Pair 1	BEEP1_MEANAMP_lefttemporal & BEEP2_MEANAMP_lefttemporal	53	.440	.001	
Pair 2	BEEP1_MeanAmp_rightemporal & BEEP2_MeanAmp_righttemporal	53	.484	.000	
Pair 3	beep1_meanamp_central & beep2_meanamp_central	53	.671	.000	
Pair 4	beep1_maxampl_lefttemporal & beep2_maxampl_lefttemporal	53	.446	.001	
Pair 5	beep1_maxampl_righttemporal & beep2_maxampl_righttemporal	53	.576	.000	
Pair 6	beep1_maxampl_central & beep2_maxampl_central	53	.403	.003	

Paired Samples Test	
	Paired Differences	
	Mean	Std. Deviation	Std. Error Mean	95% Confidence Interval of the Difference	
				Lower	Upper	
Pair 1	BEEP1_MEANAMP_lefttemporal - BEEP2_MEANAMP_lefttemporal	.72940	2.21566	.30434	.11869	1.34012	
Pair 2	BEEP1_MeanAmp_rightemporal - BEEP2_MeanAmp_righttemporal	-.00315	2.26686	.31138	-.62797	.62167	
Pair 3	beep1_meanamp_central - beep2_meanamp_central	-1.36235	1.26994	.17444	-1.71239	-1.01231	
Pair 4	beep1_maxampl_lefttemporal - beep2_maxampl_lefttemporal	.98920	2.22432	.30553	.37611	1.60230	
Pair 5	beep1_maxampl_righttemporal - beep2_maxampl_righttemporal	.30986	2.34732	.32243	-.33715	.95686	
Pair 6	beep1_maxampl_central - beep2_maxampl_central	-.62254	1.44807	.19891	-1.02168	-.22341	

Paired Samples Test	
	t	df	Sig. (2-tailed)	
				
				
Pair 1	BEEP1_MEANAMP_lefttemporal - BEEP2_MEANAMP_lefttemporal	2.397	52	.020	
Pair 2	BEEP1_MeanAmp_rightemporal - BEEP2_MeanAmp_righttemporal	-.010	52	.992	
Pair 3	beep1_meanamp_central - beep2_meanamp_central	-7.810	52	.000	
Pair 4	beep1_maxampl_lefttemporal - beep2_maxampl_lefttemporal	3.238	52	.002	
Pair 5	beep1_maxampl_righttemporal - beep2_maxampl_righttemporal	.961	52	.341	
Pair 6	beep1_maxampl_central - beep2_maxampl_central	-3.130	52	.003	

General Linear Model - 2 (group)x 2 (paired-tone) RM ANOVA to explore the infant P50 maximum amplitude in the central region

Within-Subjects Factors	
Measure:   MEASURE_1  	
PairedTone	Dependent Variable	
1	BEEP1_MAXAMP_CENTRAL	
2	BEEP2_MAXAMP_CENTRAL	

Between-Subjects Factors	
	Value Label	N	
FINALGROUPS	1.00	SZT	14	
	2.00	CONTROL	21	

Descriptive Statistics	
	FINALGROUPS	Mean	Std. Deviation	N	
BEEP1_MAXAMP_CENTRAL	SZT	5.5203	3.86024	14	
	CONTROL	5.5485	4.85131	21	
	Total	5.5372	4.42064	35	
BEEP2_MAXAMP_CENTRAL	SZT	3.6150	5.84864	14	
	CONTROL	2.8519	5.49569	21	
	Total	3.1571	5.56679	35	

Box's Test of Equality of Covariance Matricesa	
Box's M	.874	
F	.271	
df1	3	
df2	38506.192	
Sig.	.846	

Tests the null hypothesis that the observed covariance matrices of the dependent variables are equal across groups.a	
a. Design: Intercept + FINALGROUPS 
 Within Subjects Design: PairedTone	

Multivariate Testsa	
Effect	Value	F	Hypothesis df	Error df	Sig.	
PairedTone	Pillai's Trace	.101	3.717b	1.000	33.000	.063	
	Wilks' Lambda	.899	3.717b	1.000	33.000	.063	
	Hotelling's Trace	.113	3.717b	1.000	33.000	.063	
	Roy's Largest Root	.113	3.717b	1.000	33.000	.063	
PairedTone * FINALGROUPS	Pillai's Trace	.003	.110b	1.000	33.000	.742	
	Wilks' Lambda	.997	.110b	1.000	33.000	.742	
	Hotelling's Trace	.003	.110b	1.000	33.000	.742	
	Roy's Largest Root	.003	.110b	1.000	33.000	.742	

Multivariate Testsa	
Effect	Partial Eta Squared	Noncent. Parameter	Observed Powerc	
PairedTone	Pillai's Trace	.101	3.717	.465	
	Wilks' Lambda	.101	3.717	.465	
	Hotelling's Trace	.101	3.717	.465	
	Roy's Largest Root	.101	3.717	.465	
PairedTone * FINALGROUPS	Pillai's Trace	.003	.110	.062	
	Wilks' Lambda	.003	.110	.062	
	Hotelling's Trace	.003	.110	.062	
	Roy's Largest Root	.003	.110	.062	

a. Design: Intercept + FINALGROUPS 
 Within Subjects Design: PairedTone	
b. Exact statistic	
c. Computed using alpha = .05	

Mauchly's Test of Sphericitya	
Measure:   MEASURE_1  	
Within Subjects Effect	Mauchly's W	Approx. Chi-Square	df	Sig.	Epsilonb	
					Greenhouse-Geisser	Huynh-Feldt	
PairedTone	1.000	.000	0	.	1.000	1.000	

Mauchly's Test of Sphericitya	
Measure:   MEASURE_1  	
Within Subjects Effect	Epsilon	
	Lower-bound	
PairedTone	1.000	

Tests the null hypothesis that the error covariance matrix of the orthonormalized transformed dependent variables is proportional to an identity matrix.a	
a. Design: Intercept + FINALGROUPS 
 Within Subjects Design: PairedTone	
b. May be used to adjust the degrees of freedom for the averaged tests of significance. Corrected tests are displayed in the Tests of Within-Subjects Effects table.	

Tests of Within-Subjects Effects	
Measure:   MEASURE_1  	
Source	Type III Sum of Squares	df	Mean Square	F	Sig.	
PairedTone	Sphericity Assumed	88.947	1	88.947	3.717	.063	
	Greenhouse-Geisser	88.947	1.000	88.947	3.717	.063	
	Huynh-Feldt	88.947	1.000	88.947	3.717	.063	
	Lower-bound	88.947	1.000	88.947	3.717	.063	
PairedTone * FINALGROUPS	Sphericity Assumed	2.629	1	2.629	.110	.742	
	Greenhouse-Geisser	2.629	1.000	2.629	.110	.742	
	Huynh-Feldt	2.629	1.000	2.629	.110	.742	
	Lower-bound	2.629	1.000	2.629	.110	.742	
Error(PairedTone)	Sphericity Assumed	789.785	33	23.933			
	Greenhouse-Geisser	789.785	33.000	23.933			
	Huynh-Feldt	789.785	33.000	23.933			
	Lower-bound	789.785	33.000	23.933			

Tests of Within-Subjects Effects	
Measure:   MEASURE_1  	
Source	Partial Eta Squared	Noncent. Parameter	Observed Powera	
PairedTone	Sphericity Assumed	.101	3.717	.465	
	Greenhouse-Geisser	.101	3.717	.465	
	Huynh-Feldt	.101	3.717	.465	
	Lower-bound	.101	3.717	.465	
PairedTone * FINALGROUPS	Sphericity Assumed	.003	.110	.062	
	Greenhouse-Geisser	.003	.110	.062	
	Huynh-Feldt	.003	.110	.062	
	Lower-bound	.003	.110	.062	
Error(PairedTone)	Sphericity Assumed				
	Greenhouse-Geisser				
	Huynh-Feldt				
	Lower-bound				

a. Computed using alpha = .05	

Tests of Within-Subjects Contrasts	
Measure:   MEASURE_1  	
Source	PairedTone	Type III Sum of Squares	df	Mean Square	F	Sig.	Partial Eta Squared	
PairedTone	Linear	88.947	1	88.947	3.717	.063	.101	
PairedTone * FINALGROUPS	Linear	2.629	1	2.629	.110	.742	.003	
Error(PairedTone)	Linear	789.785	33	23.933				

Tests of Within-Subjects Contrasts	
Measure:   MEASURE_1  	
Source	PairedTone	Noncent. Parameter	Observed Powera	
PairedTone	Linear	3.717	.465	
PairedTone * FINALGROUPS	Linear	.110	.062	
Error(PairedTone)	Linear			

a. Computed using alpha = .05	

Levene's Test of Equality of Error Variancesa	
	F	df1	df2	Sig.	
BEEP1_MAXAMP_CENTRAL	.767	1	33	.387	
BEEP2_MAXAMP_CENTRAL	.032	1	33	.860	

Tests the null hypothesis that the error variance of the dependent variable is equal across groups.a	
a. Design: Intercept + FINALGROUPS 
 Within Subjects Design: PairedTone	

Tests of Between-Subjects Effects	
Measure:   MEASURE_1  	
Transformed Variable:   Average  	
Source	Type III Sum of Squares	df	Mean Square	F	Sig.	Partial Eta Squared	Noncent. Parameter	
Intercept	1291.503	1	1291.503	46.156	.000	.583	46.156	
FINALGROUPS	2.268	1	2.268	.081	.778	.002	.081	
Error	923.377	33	27.981					

Tests of Between-Subjects Effects	
Measure:   MEASURE_1  	
Transformed Variable:   Average  	
Source	Observed Powera	
Intercept	1.000	
FINALGROUPS	.059	
Error		

a. Computed using alpha = .05	

Estimated Marginal Means

1. Grand Mean	
Measure:   MEASURE_1  	
Mean	Std. Error	95% Confidence Interval	
		Lower Bound	Upper Bound	
4.384	.645	3.071	5.697	

2. FINALGROUPS
Estimates	
Measure:   MEASURE_1  	
FINALGROUPS	Mean	Std. Error	95% Confidence Interval	
			Lower Bound	Upper Bound	
SZT	4.568	1.000	2.534	6.601	
CONTROL	4.200	.816	2.540	5.861	

Pairwise Comparisons	
Measure:   MEASURE_1  	
(I) FINALGROUPS	(J) FINALGROUPS	Mean Difference (I-J)	Std. Error	Sig.a	95% Confidence Interval for Differencea	
					Lower Bound	Upper Bound	
SZT	CONTROL	.367	1.291	.778	-2.258	2.993	
CONTROL	SZT	-.367	1.291	.778	-2.993	2.258	

Based on estimated marginal means	
a. Adjustment for multiple comparisons: Bonferroni.	

Univariate Tests	
Measure:   MEASURE_1  	
	Sum of Squares	df	Mean Square	F	Sig.	Partial Eta Squared	Noncent. Parameter	
Contrast	1.134	1	1.134	.081	.778	.002	.081	
Error	461.689	33	13.991					

Univariate Tests	
Measure:   MEASURE_1  	
	Observed Powera	
Contrast	.059	
Error		

The F tests the effect of FINALGROUPS. This test is based on the linearly independent pairwise comparisons among the estimated marginal means.	
a. Computed using alpha = .05	

3. PairedTone
Estimates	
Measure:   MEASURE_1  	
PairedTone	Mean	Std. Error	95% Confidence Interval	
			Lower Bound	Upper Bound	
1	5.534	.774	3.959	7.109	
2	3.233	.973	1.255	5.212	

Pairwise Comparisons	
Measure:   MEASURE_1  	
(I) PairedTone	(J) PairedTone	Mean Difference (I-J)	Std. Error	Sig.a	95% Confidence Interval for Differencea	
					Lower Bound	Upper Bound	
1	2	2.301	1.194	.063	-.127	4.729	
2	1	-2.301	1.194	.063	-4.729	.127	

Based on estimated marginal means	
a. Adjustment for multiple comparisons: Bonferroni.	

Multivariate Tests	
	Value	F	Hypothesis df	Error df	Sig.	Partial Eta Squared	Noncent. Parameter	
Pillai's trace	.101	3.717a	1.000	33.000	.063	.101	3.717	
Wilks' lambda	.899	3.717a	1.000	33.000	.063	.101	3.717	
Hotelling's trace	.113	3.717a	1.000	33.000	.063	.101	3.717	
Roy's largest root	.113	3.717a	1.000	33.000	.063	.101	3.717	

Multivariate Tests	
	Observed Powerb	
Pillai's trace	.465	
Wilks' lambda	.465	
Hotelling's trace	.465	
Roy's largest root	.465	

Each F tests the multivariate effect of PairedTone. These tests are based on the linearly independent pairwise comparisons among the estimated marginal means.	
a. Exact statistic	
b. Computed using alpha = .05	

4. FINALGROUPS * PairedTone	
Measure:   MEASURE_1  	
FINALGROUPS	PairedTone	Mean	Std. Error	95% Confidence Interval	
				Lower Bound	Upper Bound	
SZT	1	5.520	1.199	3.080	7.960	
	2	3.615	1.507	.550	6.680	
CONTROL	1	5.548	.979	3.556	7.541	
	2	2.852	1.230	.349	5.355	

Profile Plots


General Linear Model  - 2 (group)x 2 (paired-tone) RM ANOVA to explore the infant P50 mean amplitude in the central region
  


Within-Subjects Factors	
Measure:   MEASURE_1  	
PairedTone	Dependent Variable	
1	BEEP1_MEANAMP_CENTRAL	
2	BEEP2_MEANAMP_CENTRAL	

Between-Subjects Factors	
	Value Label	N	
FINALGROUPS	1.00	SZT	14	
	2.00	CONTROL	21	

Descriptive Statistics	
	FINALGROUPS	Mean	Std. Deviation	N	
BEEP1_MEANAMP_CENTRAL	SZT	2.2991	3.66372	14	
	CONTROL	2.1740	4.42946	21	
	Total	2.2240	4.08379	35	
BEEP2_MEANAMP_CENTRAL	SZT	1.2822	6.18101	14	
	CONTROL	1.0132	5.58720	21	
	Total	1.1208	5.74355	35	

Box's Test of Equality of Covariance Matricesa	
Box's M	.714	
F	.221	
df1	3	
df2	38506.192	
Sig.	.882	

Tests the null hypothesis that the observed covariance matrices of the dependent variables are equal across groups.a	
a. Design: Intercept + FINALGROUPS 
 Within Subjects Design: PairedTone	

Multivariate Testsa	
Effect	Value	F	Hypothesis df	Error df	Sig.	
PairedTone	Pillai's Trace	.023	.772b	1.000	33.000	.386	
	Wilks' Lambda	.977	.772b	1.000	33.000	.386	
	Hotelling's Trace	.023	.772b	1.000	33.000	.386	
	Roy's Largest Root	.023	.772b	1.000	33.000	.386	
PairedTone * FINALGROUPS	Pillai's Trace	.000	.003b	1.000	33.000	.954	
	Wilks' Lambda	1.000	.003b	1.000	33.000	.954	
	Hotelling's Trace	.000	.003b	1.000	33.000	.954	
	Roy's Largest Root	.000	.003b	1.000	33.000	.954	

Multivariate Testsa	
Effect	Partial Eta Squared	Noncent. Parameter	Observed Powerc	
PairedTone	Pillai's Trace	.023	.772	.137	
	Wilks' Lambda	.023	.772	.137	
	Hotelling's Trace	.023	.772	.137	
	Roy's Largest Root	.023	.772	.137	
PairedTone * FINALGROUPS	Pillai's Trace	.000	.003	.050	
	Wilks' Lambda	.000	.003	.050	
	Hotelling's Trace	.000	.003	.050	
	Roy's Largest Root	.000	.003	.050	

a. Design: Intercept + FINALGROUPS 
 Within Subjects Design: PairedTone	
b. Exact statistic	
c. Computed using alpha = .05	

Mauchly's Test of Sphericitya	
Measure:   MEASURE_1  	
Within Subjects Effect	Mauchly's W	Approx. Chi-Square	df	Sig.	Epsilonb	
					Greenhouse-Geisser	Huynh-Feldt	
PairedTone	1.000	.000	0	.	1.000	1.000	

Mauchly's Test of Sphericitya	
Measure:   MEASURE_1  	
Within Subjects Effect	Epsilon	
	Lower-bound	
PairedTone	1.000	

Tests the null hypothesis that the error covariance matrix of the orthonormalized transformed dependent variables is proportional to an identity matrix.a	
a. Design: Intercept + FINALGROUPS 
 Within Subjects Design: PairedTone	
b. May be used to adjust the degrees of freedom for the averaged tests of significance. Corrected tests are displayed in the Tests of Within-Subjects Effects table.	

Tests of Within-Subjects Effects	
Measure:   MEASURE_1  	
Source	Type III Sum of Squares	df	Mean Square	F	Sig.	
PairedTone	Sphericity Assumed	19.917	1	19.917	.772	.386	
	Greenhouse-Geisser	19.917	1.000	19.917	.772	.386	
	Huynh-Feldt	19.917	1.000	19.917	.772	.386	
	Lower-bound	19.917	1.000	19.917	.772	.386	
PairedTone * FINALGROUPS	Sphericity Assumed	.087	1	.087	.003	.954	
	Greenhouse-Geisser	.087	1.000	.087	.003	.954	
	Huynh-Feldt	.087	1.000	.087	.003	.954	
	Lower-bound	.087	1.000	.087	.003	.954	
Error(PairedTone)	Sphericity Assumed	851.118	33	25.791			
	Greenhouse-Geisser	851.118	33.000	25.791			
	Huynh-Feldt	851.118	33.000	25.791			
	Lower-bound	851.118	33.000	25.791			

Tests of Within-Subjects Effects	
Measure:   MEASURE_1  	
Source	Partial Eta Squared	Noncent. Parameter	Observed Powera	
PairedTone	Sphericity Assumed	.023	.772	.137	
	Greenhouse-Geisser	.023	.772	.137	
	Huynh-Feldt	.023	.772	.137	
	Lower-bound	.023	.772	.137	
PairedTone * FINALGROUPS	Sphericity Assumed	.000	.003	.050	
	Greenhouse-Geisser	.000	.003	.050	
	Huynh-Feldt	.000	.003	.050	
	Lower-bound	.000	.003	.050	
Error(PairedTone)	Sphericity Assumed				
	Greenhouse-Geisser				
	Huynh-Feldt				
	Lower-bound				

a. Computed using alpha = .05	


Tests of Within-Subjects Contrasts	
Measure:   MEASURE_1  	
Source	PairedTone	Type III Sum of Squares	df	Mean Square	F	Sig.	Partial Eta Squared	
PairedTone	Linear	19.917	1	19.917	.772	.386	.023	
PairedTone * FINALGROUPS	Linear	.087	1	.087	.003	.954	.000	
Error(PairedTone)	Linear	851.118	33	25.791				

Tests of Within-Subjects Contrasts	
Measure:   MEASURE_1  	
Source	PairedTone	Noncent. Parameter	Observed Powera	
PairedTone	Linear	.772	.137	
PairedTone * FINALGROUPS	Linear	.003	.050	
Error(PairedTone)	Linear			

a. Computed using alpha = .05	

Levene's Test of Equality of Error Variancesa	
	F	df1	df2	Sig.	
BEEP1_MEANAMP_CENTRAL	.691	1	33	.412	
BEEP2_MEANAMP_CENTRAL	.335	1	33	.567	

Tests the null hypothesis that the error variance of the dependent variable is equal across groups.a	
a. Design: Intercept + FINALGROUPS 
 Within Subjects Design: PairedTone	

Tests of Between-Subjects Effects	
Measure:   MEASURE_1  	
Transformed Variable:   Average  	
Source	Type III Sum of Squares	df	Mean Square	F	Sig.	Partial Eta Squared	Noncent. Parameter	
Intercept	192.417	1	192.417	7.588	.009	.187	7.588	
FINALGROUPS	.652	1	.652	.026	.874	.001	.026	
Error	836.779	33	25.357					

Tests of Between-Subjects Effects	
Measure:   MEASURE_1  	
Transformed Variable:   Average  	
Source	Observed Powera	
Intercept	.762	
FINALGROUPS	.053	
Error		

a. Computed using alpha = .05	

Estimated Marginal Means

1. Grand Mean	
Measure:   MEASURE_1  	
Mean	Std. Error	95% Confidence Interval	
		Lower Bound	Upper Bound	
1.692	.614	.442	2.942	

2. FINALGROUPS
Estimates	
Measure:   MEASURE_1  	
FINALGROUPS	Mean	Std. Error	95% Confidence Interval	
			Lower Bound	Upper Bound	
SZT	1.791	.952	-.145	3.727	
CONTROL	1.594	.777	.013	3.174	

Pairwise Comparisons	
Measure:   MEASURE_1  	
(I) FINALGROUPS	(J) FINALGROUPS	Mean Difference (I-J)	Std. Error	Sig.a	95% Confidence Interval for Differencea	
					Lower Bound	Upper Bound	
SZT	CONTROL	.197	1.229	.874	-2.302	2.697	
CONTROL	SZT	-.197	1.229	.874	-2.697	2.302	

Based on estimated marginal means	
a. Adjustment for multiple comparisons: Bonferroni.	

Univariate Tests	
Measure:   MEASURE_1  	
	Sum of Squares	df	Mean Square	F	Sig.	Partial Eta Squared	Noncent. Parameter	
Contrast	.326	1	.326	.026	.874	.001	.026	
Error	418.389	33	12.678					

Univariate Tests	
Measure:   MEASURE_1  	
	Observed Powera	
Contrast	.053	
Error		

The F tests the effect of FINALGROUPS. This test is based on the linearly independent pairwise comparisons among the estimated marginal means.	
a. Computed using alpha = .05	

3. PairedTone

Estimates	
Measure:   MEASURE_1  	
PairedTone	Mean	Std. Error	95% Confidence Interval	
			Lower Bound	Upper Bound	
1	2.237	.715	.782	3.691	
2	1.148	1.005	-.898	3.193	

Pairwise Comparisons	
Measure:   MEASURE_1  	
(I) PairedTone	(J) PairedTone	Mean Difference (I-J)	Std. Error	Sig.a	95% Confidence Interval for Differencea	
					Lower Bound	Upper Bound	
1	2	1.089	1.239	.386	-1.432	3.610	
2	1	-1.089	1.239	.386	-3.610	1.432	

Based on estimated marginal means	
a. Adjustment for multiple comparisons: Bonferroni.	

Multivariate Tests	
	Value	F	Hypothesis df	Error df	Sig.	Partial Eta Squared	Noncent. Parameter	
Pillai's trace	.023	.772a	1.000	33.000	.386	.023	.772	
Wilks' lambda	.977	.772a	1.000	33.000	.386	.023	.772	
Hotelling's trace	.023	.772a	1.000	33.000	.386	.023	.772	
Roy's largest root	.023	.772a	1.000	33.000	.386	.023	.772	

Multivariate Tests	
	Observed Powerb	
Pillai's trace	.137	
Wilks' lambda	.137	
Hotelling's trace	.137	
Roy's largest root	.137	

Each F tests the multivariate effect of PairedTone. These tests are based on the linearly independent pairwise comparisons among the estimated marginal means.	
a. Exact statistic	
b. Computed using alpha = .05	


4. FINALGROUPS * PairedTone	
Measure:   MEASURE_1  	
FINALGROUPS	PairedTone	Mean	Std. Error	95% Confidence Interval	
				Lower Bound	Upper Bound	
SZT	1	2.299	1.108	.045	4.553	
	2	1.282	1.558	-1.887	4.451	
CONTROL	1	2.174	.904	.334	4.014	
	2	1.013	1.272	-1.574	3.601	

Profile Plots


General Linear Model  - A 2 (group) x 2 (paired-tone) RM ANOVA to explore the mean amplitude maternal P50 in the left-temporal region (following significant effects highlighted in earlier analyses).

Within-Subjects Factors	
Measure:   MEASURE_1  	
pairedtone	Dependent Variable	
1	BEEP1_MEANAMP_lefttemporal	
2	BEEP2_MEANAMP_lefttemporal	

Between-Subjects Factors	
	Value Label	N	
finalgroups	1.00	szt	23	
	2.00	control	30	

Descriptive Statistics	
	finalgroups	Mean	Std. Deviation	N	
BEEP1_MEANAMP_lefttemporal	szt	2.7620	1.69528	23	
	control	3.0336	1.57914	30	
	Total	2.9157	1.62021	53	
BEEP2_MEANAMP_lefttemporal	szt	2.6844	2.45118	23	
	control	1.8045	2.29816	30	
	Total	2.1863	2.38355	53	

Box's Test of Equality of Covariance Matricesa	
Box's M	.388	
F	.124	
df1	3	
df2	325739.718	
Sig.	.946	

Tests the null hypothesis that the observed covariance matrices of the dependent variables are equal across groups.a	
a. Design: Intercept + finalgroups 
 Within Subjects Design: pairedtone	

Multivariate Testsa	
Effect	Value	F	Hypothesis df	Error df	Sig.	
pairedtone	Pillai's Trace	.085	4.763b	1.000	51.000	.034	
	Wilks' Lambda	.915	4.763b	1.000	51.000	.034	
	Hotelling's Trace	.093	4.763b	1.000	51.000	.034	
	Roy's Largest Root	.093	4.763b	1.000	51.000	.034	
pairedtone * finalgroups	Pillai's Trace	.068	3.699b	1.000	51.000	.060	
	Wilks' Lambda	.932	3.699b	1.000	51.000	.060	
	Hotelling's Trace	.073	3.699b	1.000	51.000	.060	
	Roy's Largest Root	.073	3.699b	1.000	51.000	.060	

Multivariate Testsa	
Effect	Partial Eta Squared	Noncent. Parameter	Observed Powerc	
pairedtone	Pillai's Trace	.085	4.763	.572	
	Wilks' Lambda	.085	4.763	.572	
	Hotelling's Trace	.085	4.763	.572	
	Roy's Largest Root	.085	4.763	.572	
pairedtone * finalgroups	Pillai's Trace	.068	3.699	.471	
	Wilks' Lambda	.068	3.699	.471	
	Hotelling's Trace	.068	3.699	.471	
	Roy's Largest Root	.068	3.699	.471	

a. Design: Intercept + finalgroups 
 Within Subjects Design: pairedtone	
b. Exact statistic	
c. Computed using alpha = .05	

Mauchly's Test of Sphericitya	
Measure:   MEASURE_1  	
Within Subjects Effect	Mauchly's W	Approx. Chi-Square	df	Sig.	Epsilonb	
					Greenhouse-Geisser	Huynh-Feldt	
pairedtone	1.000	.000	0	.	1.000	1.000	

Mauchly's Test of Sphericitya	
Measure:   MEASURE_1  	
Within Subjects Effect	Epsilon	
	Lower-bound	
pairedtone	1.000	

Tests the null hypothesis that the error covariance matrix of the orthonormalized transformed dependent variables is proportional to an identity matrix.a	
a. Design: Intercept + finalgroups 
 Within Subjects Design: pairedtone	
b. May be used to adjust the degrees of freedom for the averaged tests of significance. Corrected tests are displayed in the Tests of Within-Subjects Effects table.	

Tests of Within-Subjects Effects	
Measure:   MEASURE_1  	
Source	Type III Sum of Squares	df	Mean Square	F	Sig.	
pairedtone	Sphericity Assumed	11.115	1	11.115	4.763	.034	
	Greenhouse-Geisser	11.115	1.000	11.115	4.763	.034	
	Huynh-Feldt	11.115	1.000	11.115	4.763	.034	
	Lower-bound	11.115	1.000	11.115	4.763	.034	
pairedtone * finalgroups	Sphericity Assumed	8.631	1	8.631	3.699	.060	
	Greenhouse-Geisser	8.631	1.000	8.631	3.699	.060	
	Huynh-Feldt	8.631	1.000	8.631	3.699	.060	
	Lower-bound	8.631	1.000	8.631	3.699	.060	
Error(pairedtone)	Sphericity Assumed	119.007	51	2.333			
	Greenhouse-Geisser	119.007	51.000	2.333			
	Huynh-Feldt	119.007	51.000	2.333			
	Lower-bound	119.007	51.000	2.333			

Tests of Within-Subjects Effects	
Measure:   MEASURE_1  	
Source	Partial Eta Squared	Noncent. Parameter	Observed Powera	
pairedtone	Sphericity Assumed	.085	4.763	.572	
	Greenhouse-Geisser	.085	4.763	.572	
	Huynh-Feldt	.085	4.763	.572	
	Lower-bound	.085	4.763	.572	
pairedtone * finalgroups	Sphericity Assumed	.068	3.699	.471	
	Greenhouse-Geisser	.068	3.699	.471	
	Huynh-Feldt	.068	3.699	.471	
	Lower-bound	.068	3.699	.471	
Error(pairedtone)	Sphericity Assumed				
	Greenhouse-Geisser				
	Huynh-Feldt				
	Lower-bound				

a. Computed using alpha = .05	


Tests of Within-Subjects Contrasts	
Measure:   MEASURE_1  	
Source	pairedtone	Type III Sum of Squares	df	Mean Square	F	Sig.	Partial Eta Squared	
pairedtone	Linear	11.115	1	11.115	4.763	.034	.085	
pairedtone * finalgroups	Linear	8.631	1	8.631	3.699	.060	.068	
Error(pairedtone)	Linear	119.007	51	2.333				

Tests of Within-Subjects Contrasts	
Measure:   MEASURE_1  	
Source	pairedtone	Noncent. Parameter	Observed Powera	
pairedtone	Linear	4.763	.572	
pairedtone * finalgroups	Linear	3.699	.471	
Error(pairedtone)	Linear			

a. Computed using alpha = .05	

Levene's Test of Equality of Error Variancesa	
	F	df1	df2	Sig.	
BEEP1_MEANAMP_lefttemporal	.398	1	51	.531	
BEEP2_MEANAMP_lefttemporal	.374	1	51	.544	

Tests the null hypothesis that the error variance of the dependent variable is equal across groups.a	
a. Design: Intercept + finalgroups 
 Within Subjects Design: pairedtone	

Tests of Between-Subjects Effects	
Measure:   MEASURE_1  	
Transformed Variable:   Average  	
Source	Type III Sum of Squares	df	Mean Square	F	Sig.	Partial Eta Squared	Noncent. Parameter	
Intercept	688.508	1	688.508	116.316	.000	.695	116.316	
finalgroups	2.410	1	2.410	.407	.526	.008	.407	
Error	301.884	51	5.919					

Tests of Between-Subjects Effects	
Measure:   MEASURE_1  	
Transformed Variable:   Average  	
Source	Observed Powera	
Intercept	1.000	
finalgroups	.096	
Error		

a. Computed using alpha = .05	

Estimated Marginal Means

1. Grand Mean	
Measure:   MEASURE_1  	
Mean	Std. Error	95% Confidence Interval	
		Lower Bound	Upper Bound	
2.571	.238	2.093	3.050	

2. finalgroups

Estimates	
Measure:   MEASURE_1  	
finalgroups	Mean	Std. Error	95% Confidence Interval	
			Lower Bound	Upper Bound	
szt	2.723	.359	2.003	3.443	
control	2.419	.314	1.788	3.050	

Pairwise Comparisons	
Measure:   MEASURE_1  	
(I) finalgroups	(J) finalgroups	Mean Difference (I-J)	Std. Error	Sig.a	95% Confidence Interval for Differencea	
					Lower Bound	Upper Bound	
szt	control	.304	.477	.526	-.653	1.261	
control	szt	-.304	.477	.526	-1.261	.653	

Based on estimated marginal means	
a. Adjustment for multiple comparisons: Least Significant Difference (equivalent to no adjustments).	

Univariate Tests	
Measure:   MEASURE_1  	
	Sum of Squares	df	Mean Square	F	Sig.	Partial Eta Squared	Noncent. Parameter	
Contrast	1.205	1	1.205	.407	.526	.008	.407	
Error	150.942	51	2.960					

Univariate Tests	
Measure:   MEASURE_1  	
	Observed Powera	
Contrast	.096	
Error		

The F tests the effect of finalgroups. This test is based on the linearly independent pairwise comparisons among the estimated marginal means.	
a. Computed using alpha = .05	

3. pairedtone

Estimates	
Measure:   MEASURE_1  	
pairedtone	Mean	Std. Error	95% Confidence Interval	
			Lower Bound	Upper Bound	
1	2.898	.226	2.444	3.351	
2	2.244	.328	1.586	2.902	

Pairwise Comparisons	
Measure:   MEASURE_1  	
(I) pairedtone	(J) pairedtone	Mean Difference (I-J)	Std. Error	Sig.b	95% Confidence Interval for Differenceb	
					Lower Bound	Upper Bound	
1	2	.653*	.299	.034	.052	1.254	
2	1	-.653*	.299	.034	-1.254	-.052	

Based on estimated marginal means	
*. The mean difference is significant at the .05 level.	
b. Adjustment for multiple comparisons: Least Significant Difference (equivalent to no adjustments).	

Multivariate Tests	
	Value	F	Hypothesis df	Error df	Sig.	Partial Eta Squared	Noncent. Parameter	
Pillai's trace	.085	4.763a	1.000	51.000	.034	.085	4.763	
Wilks' lambda	.915	4.763a	1.000	51.000	.034	.085	4.763	
Hotelling's trace	.093	4.763a	1.000	51.000	.034	.085	4.763	
Roy's largest root	.093	4.763a	1.000	51.000	.034	.085	4.763	

Multivariate Tests	
	Observed Powerb	
Pillai's trace	.572	
Wilks' lambda	.572	
Hotelling's trace	.572	
Roy's largest root	.572	

Each F tests the multivariate effect of pairedtone. These tests are based on the linearly independent pairwise comparisons among the estimated marginal means.	
a. Exact statistic	
b. Computed using alpha = .05	


4. finalgroups * pairedtone

Estimates	
Measure:   MEASURE_1  	
finalgroups	pairedtone	Mean	Std. Error	95% Confidence Interval	
				Lower Bound	Upper Bound	
szt	1	2.762	.340	2.080	3.444	
	2	2.684	.493	1.694	3.675	
control	1	3.034	.298	2.436	3.631	
	2	1.804	.432	.937	2.671	

Pairwise Comparisons	
Measure:   MEASURE_1  	
pairedtone	(I) finalgroups	(J) finalgroups	Mean Difference (I-J)	Std. Error	Sig.a	95% Confidence Interval for Differencea	
						Lower Bound	Upper Bound	
1	szt	control	-.272	.452	.551	-1.179	.636	
	control	szt	.272	.452	.551	-.636	1.179	
2	szt	control	.880	.656	.185	-.436	2.196	
	control	szt	-.880	.656	.185	-2.196	.436	

Based on estimated marginal means	
a. Adjustment for multiple comparisons: Bonferroni.	

Univariate Tests	
Measure:   MEASURE_1  	
pairedtone	Sum of Squares	df	Mean Square	F	Sig.	Partial Eta Squared	Noncent. Parameter	
1	Contrast	.960	1	.960	.361	.551	.007	.361	
	Error	135.544	51	2.658					
2	Contrast	10.081	1	10.081	1.802	.185	.034	1.802	
	Error	285.347	51	5.595					

Univariate Tests	
Measure:   MEASURE_1  	
pairedtone	Observed Powera	
1	Contrast	.091	
	Error		
2	Contrast	.261	
	Error		

Each F tests the simple effects of finalgroups within each level combination of the other effects shown. These tests are based on the linearly independent pairwise comparisons among the estimated marginal means.	
a. Computed using alpha = .05	

5. finalgroups * pairedtone

Estimates	
Measure:   MEASURE_1  	
finalgroups	pairedtone	Mean	Std. Error	95% Confidence Interval	
				Lower Bound	Upper Bound	
szt	1	2.762	.340	2.080	3.444	
	2	2.684	.493	1.694	3.675	
control	1	3.034	.298	2.436	3.631	
	2	1.804	.432	.937	2.671	

Pairwise Comparisons	
Measure:   MEASURE_1  	
finalgroups	(I) pairedtone	(J) pairedtone	Mean Difference (I-J)	Std. Error	Sig.b	95% Confidence Interval for Differenceb	
						Lower Bound	Upper Bound	
szt	1	2	.078	.450	.864	-.827	.982	
	2	1	-.078	.450	.864	-.982	.827	
control	1	2	1.229*	.394	.003	.437	2.021	
	2	1	-1.229*	.394	.003	-2.021	-.437	

Based on estimated marginal means	
*. The mean difference is significant at the .05 level.	
b. Adjustment for multiple comparisons: Bonferroni.	

Multivariate Tests	
finalgroups	Value	F	Hypothesis df	Error df	Sig.	Partial Eta Squared	Noncent. Parameter	
szt	Pillai's trace	.001	.030a	1.000	51.000	.864	.001	.030	
	Wilks' lambda	.999	.030a	1.000	51.000	.864	.001	.030	
	Hotelling's trace	.001	.030a	1.000	51.000	.864	.001	.030	
	Roy's largest root	.001	.030a	1.000	51.000	.864	.001	.030	
control	Pillai's trace	.160	9.711a	1.000	51.000	.003	.160	9.711	
	Wilks' lambda	.840	9.711a	1.000	51.000	.003	.160	9.711	
	Hotelling's trace	.190	9.711a	1.000	51.000	.003	.160	9.711	
	Roy's largest root	.190	9.711a	1.000	51.000	.003	.160	9.711	

Multivariate Tests	
finalgroups	Observed Powerb	
szt	Pillai's trace	.053	
	Wilks' lambda	.053	
	Hotelling's trace	.053	
	Roy's largest root	.053	
control	Pillai's trace	.864	
	Wilks' lambda	.864	
	Hotelling's trace	.864	
	Roy's largest root	.864	

Each F tests the multivariate simple effects of pairedtone within each level combination of the other effects shown. These tests are based on the linearly independent pairwise comparisons among the estimated marginal means.	
a. Exact statistic	
b. Computed using alpha = .05	

Profile Plots


General Linear Model -  -  A 2 (group) x 2 (paired-tone) RM ANOVA to explore the maximum amplitude maternal P50 in the central region (following significant effects highlighted in earlier analyses).

Within-Subjects Factors	
Measure:   MEASURE_1  	
pairedtone	Dependent Variable	
1	beep1_maxampl_central	
2	beep2_maxampl_central	

Between-Subjects Factors	
	Value Label	N	
finalgroups	1.00	szt	23	
	2.00	control	30	

Descriptive Statistics	
	finalgroups	Mean	Std. Deviation	N	
beep1_maxampl_central	szt	-1.1141	1.54380	23	
	control	-1.4534	1.24239	30	
	Total	-1.3061	1.37766	53	
beep2_maxampl_central	szt	-1.0285	1.44126	23	
	control	-.4191	1.06857	30	
	Total	-.6836	1.26831	53	

Box's Test of Equality of Covariance Matricesa	
Box's M	3.997	
F	1.274	
df1	3	
df2	325739.718	
Sig.	.281	

Tests the null hypothesis that the observed covariance matrices of the dependent variables are equal across groups.a	
a. Design: Intercept + finalgroups 
 Within Subjects Design: pairedtone	

Multivariate Testsa	
Effect	Value	F	Hypothesis df	Error df	Sig.	
pairedtone	Pillai's Trace	.144	8.555b	1.000	51.000	.005	
	Wilks' Lambda	.856	8.555b	1.000	51.000	.005	
	Hotelling's Trace	.168	8.555b	1.000	51.000	.005	
	Roy's Largest Root	.168	8.555b	1.000	51.000	.005	
pairedtone * finalgroups	Pillai's Trace	.107	6.139b	1.000	51.000	.017	
	Wilks' Lambda	.893	6.139b	1.000	51.000	.017	
	Hotelling's Trace	.120	6.139b	1.000	51.000	.017	
	Roy's Largest Root	.120	6.139b	1.000	51.000	.017	

Multivariate Testsa	
Effect	Partial Eta Squared	Noncent. Parameter	Observed Powerc	
pairedtone	Pillai's Trace	.144	8.555	.818	
	Wilks' Lambda	.144	8.555	.818	
	Hotelling's Trace	.144	8.555	.818	
	Roy's Largest Root	.144	8.555	.818	
pairedtone * finalgroups	Pillai's Trace	.107	6.139	.681	
	Wilks' Lambda	.107	6.139	.681	
	Hotelling's Trace	.107	6.139	.681	
	Roy's Largest Root	.107	6.139	.681	

a. Design: Intercept + finalgroups 
 Within Subjects Design: pairedtone	
b. Exact statistic	
c. Computed using alpha = .05	

Mauchly's Test of Sphericitya	
Measure:   MEASURE_1  	
Within Subjects Effect	Mauchly's W	Approx. Chi-Square	df	Sig.	Epsilonb	
					Greenhouse-Geisser	Huynh-Feldt	
pairedtone	1.000	.000	0	.	1.000	1.000	

Mauchly's Test of Sphericitya	
Measure:   MEASURE_1  	
Within Subjects Effect	Epsilon	
	Lower-bound	
pairedtone	1.000	

Tests the null hypothesis that the error covariance matrix of the orthonormalized transformed dependent variables is proportional to an identity matrix.a	
a. Design: Intercept + finalgroups 
 Within Subjects Design: pairedtone	
b. May be used to adjust the degrees of freedom for the averaged tests of significance. Corrected tests are displayed in the Tests of Within-Subjects Effects table.	

Tests of Within-Subjects Effects	
Measure:   MEASURE_1  	
Source	Type III Sum of Squares	df	Mean Square	F	Sig.	
pairedtone	Sphericity Assumed	8.162	1	8.162	8.555	.005	
	Greenhouse-Geisser	8.162	1.000	8.162	8.555	.005	
	Huynh-Feldt	8.162	1.000	8.162	8.555	.005	
	Lower-bound	8.162	1.000	8.162	8.555	.005	
pairedtone * finalgroups	Sphericity Assumed	5.858	1	5.858	6.139	.017	
	Greenhouse-Geisser	5.858	1.000	5.858	6.139	.017	
	Huynh-Feldt	5.858	1.000	5.858	6.139	.017	
	Lower-bound	5.858	1.000	5.858	6.139	.017	
Error(pairedtone)	Sphericity Assumed	48.662	51	.954			
	Greenhouse-Geisser	48.662	51.000	.954			
	Huynh-Feldt	48.662	51.000	.954			
	Lower-bound	48.662	51.000	.954			

Tests of Within-Subjects Effects	
Measure:   MEASURE_1  	
Source	Partial Eta Squared	Noncent. Parameter	Observed Powera	
pairedtone	Sphericity Assumed	.144	8.555	.818	
	Greenhouse-Geisser	.144	8.555	.818	
	Huynh-Feldt	.144	8.555	.818	
	Lower-bound	.144	8.555	.818	
pairedtone * finalgroups	Sphericity Assumed	.107	6.139	.681	
	Greenhouse-Geisser	.107	6.139	.681	
	Huynh-Feldt	.107	6.139	.681	
	Lower-bound	.107	6.139	.681	
Error(pairedtone)	Sphericity Assumed				
	Greenhouse-Geisser				
	Huynh-Feldt				
	Lower-bound				

a. Computed using alpha = .05	

Tests of Within-Subjects Contrasts	
Measure:   MEASURE_1  	
Source	pairedtone	Type III Sum of Squares	df	Mean Square	F	Sig.	Partial Eta Squared	
pairedtone	Linear	8.162	1	8.162	8.555	.005	.144	
pairedtone * finalgroups	Linear	5.858	1	5.858	6.139	.017	.107	
Error(pairedtone)	Linear	48.662	51	.954				

Tests of Within-Subjects Contrasts	
Measure:   MEASURE_1  	
Source	pairedtone	Noncent. Parameter	Observed Powera	
pairedtone	Linear	8.555	.818	
pairedtone * finalgroups	Linear	6.139	.681	
Error(pairedtone)	Linear			

a. Computed using alpha = .05	

Levene's Test of Equality of Error Variancesa	
	F	df1	df2	Sig.	
beep1_maxampl_central	.103	1	51	.750	
beep2_maxampl_central	1.745	1	51	.192	

Tests the null hypothesis that the error variance of the dependent variable is equal across groups.a	
a. Design: Intercept + finalgroups 
 Within Subjects Design: pairedtone	

Tests of Between-Subjects Effects	
Measure:   MEASURE_1  	
Transformed Variable:   Average  	
Source	Type III Sum of Squares	df	Mean Square	F	Sig.	Partial Eta Squared	Noncent. Parameter	
Intercept	104.943	1	104.943	42.028	.000	.452	42.028	
finalgroups	.475	1	.475	.190	.665	.004	.190	
Error	127.346	51	2.497					

Tests of Between-Subjects Effects	
Measure:   MEASURE_1  	
Transformed Variable:   Average  	
Source	Observed Powera	
Intercept	1.000	
finalgroups	.071	
Error		

a. Computed using alpha = .05	

Estimated Marginal Means

1. Grand Mean	
Measure:   MEASURE_1  	
Mean	Std. Error	95% Confidence Interval	
		Lower Bound	Upper Bound	
-1.004	.155	-1.315	-.693	

2. finalgroups

Estimates	
Measure:   MEASURE_1  	
finalgroups	Mean	Std. Error	95% Confidence Interval	
			Lower Bound	Upper Bound	
szt	-1.071	.233	-1.539	-.604	
control	-.936	.204	-1.346	-.527	

Pairwise Comparisons	
Measure:   MEASURE_1  	
(I) finalgroups	(J) finalgroups	Mean Difference (I-J)	Std. Error	Sig.a	95% Confidence Interval for Differencea	
					Lower Bound	Upper Bound	
szt	control	-.135	.310	.665	-.757	.487	
control	szt	.135	.310	.665	-.487	.757	

Based on estimated marginal means	
a. Adjustment for multiple comparisons: Least Significant Difference (equivalent to no adjustments).	

Univariate Tests	
Measure:   MEASURE_1  	
	Sum of Squares	df	Mean Square	F	Sig.	Partial Eta Squared	Noncent. Parameter	
Contrast	.238	1	.238	.190	.665	.004	.190	
Error	63.673	51	1.248					

Univariate Tests	
Measure:   MEASURE_1  	
	Observed Powera	
Contrast	.071	
Error		

The F tests the effect of finalgroups. This test is based on the linearly independent pairwise comparisons among the estimated marginal means.	
a. Computed using alpha = .05	

3. pairedtone
Estimates	
Measure:   MEASURE_1  	
pairedtone	Mean	Std. Error	95% Confidence Interval	
			Lower Bound	Upper Bound	
1	-1.284	.191	-1.668	-.900	
2	-.724	.172	-1.070	-.378	

Pairwise Comparisons	
Measure:   MEASURE_1  	
(I) pairedtone	(J) pairedtone	Mean Difference (I-J)	Std. Error	Sig.b	95% Confidence Interval for Differenceb	
					Lower Bound	Upper Bound	
1	2	-.560*	.191	.005	-.944	-.176	
2	1	.560*	.191	.005	.176	.944	

Based on estimated marginal means	
*. The mean difference is significant at the .05 level.	
b. Adjustment for multiple comparisons: Least Significant Difference (equivalent to no adjustments).	

Multivariate Tests	
	Value	F	Hypothesis df	Error df	Sig.	Partial Eta Squared	Noncent. Parameter	
Pillai's trace	.144	8.555a	1.000	51.000	.005	.144	8.555	
Wilks' lambda	.856	8.555a	1.000	51.000	.005	.144	8.555	
Hotelling's trace	.168	8.555a	1.000	51.000	.005	.144	8.555	
Roy's largest root	.168	8.555a	1.000	51.000	.005	.144	8.555	

Multivariate Tests	
	Observed Powerb	
Pillai's trace	.818	
Wilks' lambda	.818	
Hotelling's trace	.818	
Roy's largest root	.818	

Each F tests the multivariate effect of pairedtone. These tests are based on the linearly independent pairwise comparisons among the estimated marginal means.	
a. Exact statistic	
b. Computed using alpha = .05	

4. finalgroups * pairedtone

Estimates	
Measure:   MEASURE_1  	
finalgroups	pairedtone	Mean	Std. Error	95% Confidence Interval	
				Lower Bound	Upper Bound	
szt	1	-1.114	.288	-1.692	-.536	
	2	-1.029	.259	-1.549	-.508	
control	1	-1.453	.252	-1.959	-.947	
	2	-.419	.227	-.875	.037	

Pairwise Comparisons	
Measure:   MEASURE_1  	
pairedtone	(I) finalgroups	(J) finalgroups	Mean Difference (I-J)	Std. Error	Sig.a	95% Confidence Interval for Differencea	
						Lower Bound	Upper Bound	
1	szt	control	.339	.383	.379	-.429	1.107	
	control	szt	-.339	.383	.379	-1.107	.429	
2	szt	control	-.609	.345	.083	-1.301	.082	
	control	szt	.609	.345	.083	-.082	1.301	

Based on estimated marginal means	
a. Adjustment for multiple comparisons: Bonferroni.	

Univariate Tests	
Measure:   MEASURE_1  	
pairedtone	Sum of Squares	df	Mean Square	F	Sig.	Partial Eta Squared	Noncent. Parameter	
1	Contrast	1.498	1	1.498	.786	.379	.015	.786	
	Error	97.196	51	1.906					
2	Contrast	4.835	1	4.835	3.129	.083	.058	3.129	
	Error	78.812	51	1.545					

Univariate Tests	
Measure:   MEASURE_1  	
pairedtone	Observed Powera	
1	Contrast	.140	
	Error		
2	Contrast	.411	
	Error		

Each F tests the simple effects of finalgroups within each level combination of the other effects shown. These tests are based on the linearly independent pairwise comparisons among the estimated marginal means.	
a. Computed using alpha = .05	

5. finalgroups * pairedtone
Estimates	
Measure:   MEASURE_1  	
finalgroups	pairedtone	Mean	Std. Error	95% Confidence Interval	
				Lower Bound	Upper Bound	
szt	1	-1.114	.288	-1.692	-.536	
	2	-1.029	.259	-1.549	-.508	
control	1	-1.453	.252	-1.959	-.947	
	2	-.419	.227	-.875	.037	

Pairwise Comparisons	
Measure:   MEASURE_1  	
finalgroups	(I) pairedtone	(J) pairedtone	Mean Difference (I-J)	Std. Error	Sig.b	95% Confidence Interval for Differenceb	
						Lower Bound	Upper Bound	
szt	1	2	-.086	.288	.768	-.664	.493	
	2	1	.086	.288	.768	-.493	.664	
control	1	2	-1.034*	.252	.000	-1.541	-.528	
	2	1	1.034*	.252	.000	.528	1.541	

Based on estimated marginal means	
*. The mean difference is significant at the .05 level.	
b. Adjustment for multiple comparisons: Bonferroni.	

Multivariate Tests	
finalgroups	Value	F	Hypothesis df	Error df	Sig.	Partial Eta Squared	Noncent. Parameter	
szt	Pillai's trace	.002	.088a	1.000	51.000	.768	.002	.088	
	Wilks' lambda	.998	.088a	1.000	51.000	.768	.002	.088	
	Hotelling's trace	.002	.088a	1.000	51.000	.768	.002	.088	
	Roy's largest root	.002	.088a	1.000	51.000	.768	.002	.088	
control	Pillai's trace	.248	16.815a	1.000	51.000	.000	.248	16.815	
	Wilks' lambda	.752	16.815a	1.000	51.000	.000	.248	16.815	
	Hotelling's trace	.330	16.815a	1.000	51.000	.000	.248	16.815	
	Roy's largest root	.330	16.815a	1.000	51.000	.000	.248	16.815	

Multivariate Tests	
finalgroups	Observed Powerb	
szt	Pillai's trace	.060	
	Wilks' lambda	.060	
	Hotelling's trace	.060	
	Roy's largest root	.060	
control	Pillai's trace	.980	
	Wilks' lambda	.980	
	Hotelling's trace	.980	
	Roy's largest root	.980	

Each F tests the multivariate simple effects of pairedtone within each level combination of the other effects shown. These tests are based on the linearly independent pairwise comparisons among the estimated marginal means.	
a. Exact statistic	
b. Computed using alpha = .05	

Profile Plots


General Linear Model  -  A 2 (group) x 2 (paired-tone) RM ANOVA to explore the maximum amplitude maternal P50 in the left-temporal region (following significant effects highlighted in earlier analyses).

Within-Subjects Factors	
Measure:   MEASURE_1  	
pairedtone	Dependent Variable	
1	beep1_maxampl_lefttemporal	
2	beep2_maxampl_lefttemporal	

Between-Subjects Factors	
	Value Label	N	
finalgroups	1.00	szt	23	
	2.00	control	30	

Descriptive Statistics	
	finalgroups	Mean	Std. Deviation	N	
beep1_maxampl_lefttemporal	szt	3.9213	1.92084	23	
	control	4.2487	1.58786	30	
	Total	4.1066	1.73030	53	
beep2_maxampl_lefttemporal	szt	3.5760	2.54539	23	
	control	2.7658	2.20080	30	
	Total	3.1174	2.36784	53	

Box's Test of Equality of Covariance Matricesa	
Box's M	2.040	
F	.650	
df1	3	
df2	325739.718	
Sig.	.583	

Tests the null hypothesis that the observed covariance matrices of the dependent variables are equal across groups.a	
a. Design: Intercept + finalgroups 
 Within Subjects Design: pairedtone	

Multivariate Testsa	
Effect	Value	F	Hypothesis df	Error df	Sig.	
pairedtone	Pillai's Trace	.153	9.230b	1.000	51.000	.004	
	Wilks' Lambda	.847	9.230b	1.000	51.000	.004	
	Hotelling's Trace	.181	9.230b	1.000	51.000	.004	
	Roy's Largest Root	.181	9.230b	1.000	51.000	.004	
pairedtone * finalgroups	Pillai's Trace	.065	3.574b	1.000	51.000	.064	
	Wilks' Lambda	.935	3.574b	1.000	51.000	.064	
	Hotelling's Trace	.070	3.574b	1.000	51.000	.064	
	Roy's Largest Root	.070	3.574b	1.000	51.000	.064	

Multivariate Testsa	
Effect	Partial Eta Squared	Noncent. Parameter	Observed Powerc	
pairedtone	Pillai's Trace	.153	9.230	.846	
	Wilks' Lambda	.153	9.230	.846	
	Hotelling's Trace	.153	9.230	.846	
	Roy's Largest Root	.153	9.230	.846	
pairedtone * finalgroups	Pillai's Trace	.065	3.574	.458	
	Wilks' Lambda	.065	3.574	.458	
	Hotelling's Trace	.065	3.574	.458	
	Roy's Largest Root	.065	3.574	.458	

a. Design: Intercept + finalgroups 
 Within Subjects Design: pairedtone	
b. Exact statistic	
c. Computed using alpha = .05	

Mauchly's Test of Sphericitya	
Measure:   MEASURE_1  	
Within Subjects Effect	Mauchly's W	Approx. Chi-Square	df	Sig.	Epsilonb	
					Greenhouse-Geisser	Huynh-Feldt	
pairedtone	1.000	.000	0	.	1.000	1.000	

Mauchly's Test of Sphericitya	
Measure:   MEASURE_1  	
Within Subjects Effect	Epsilon	
	Lower-bound	
pairedtone	1.000	

Tests the null hypothesis that the error covariance matrix of the orthonormalized transformed dependent variables is proportional to an identity matrix.a	
a. Design: Intercept + finalgroups 
 Within Subjects Design: pairedtone	
b. May be used to adjust the degrees of freedom for the averaged tests of significance. Corrected tests are displayed in the Tests of Within-Subjects Effects table.	

Tests of Within-Subjects Effects	
Measure:   MEASURE_1  	
Source	Type III Sum of Squares	df	Mean Square	F	Sig.	
pairedtone	Sphericity Assumed	21.756	1	21.756	9.230	.004	
	Greenhouse-Geisser	21.756	1.000	21.756	9.230	.004	
	Huynh-Feldt	21.756	1.000	21.756	9.230	.004	
	Lower-bound	21.756	1.000	21.756	9.230	.004	
pairedtone * finalgroups	Sphericity Assumed	8.424	1	8.424	3.574	.064	
	Greenhouse-Geisser	8.424	1.000	8.424	3.574	.064	
	Huynh-Feldt	8.424	1.000	8.424	3.574	.064	
	Lower-bound	8.424	1.000	8.424	3.574	.064	
Error(pairedtone)	Sphericity Assumed	120.214	51	2.357			
	Greenhouse-Geisser	120.214	51.000	2.357			
	Huynh-Feldt	120.214	51.000	2.357			
	Lower-bound	120.214	51.000	2.357			

Tests of Within-Subjects Effects	
Measure:   MEASURE_1  	
Source	Partial Eta Squared	Noncent. Parameter	Observed Powera	
pairedtone	Sphericity Assumed	.153	9.230	.846	
	Greenhouse-Geisser	.153	9.230	.846	
	Huynh-Feldt	.153	9.230	.846	
	Lower-bound	.153	9.230	.846	
pairedtone * finalgroups	Sphericity Assumed	.065	3.574	.458	
	Greenhouse-Geisser	.065	3.574	.458	
	Huynh-Feldt	.065	3.574	.458	
	Lower-bound	.065	3.574	.458	
Error(pairedtone)	Sphericity Assumed				
	Greenhouse-Geisser				
	Huynh-Feldt				
	Lower-bound				

a. Computed using alpha = .05	

Tests of Within-Subjects Contrasts	
Measure:   MEASURE_1  	
Source	pairedtone	Type III Sum of Squares	df	Mean Square	F	Sig.	Partial Eta Squared	
pairedtone	Linear	21.756	1	21.756	9.230	.004	.153	
pairedtone * finalgroups	Linear	8.424	1	8.424	3.574	.064	.065	
Error(pairedtone)	Linear	120.214	51	2.357				

Tests of Within-Subjects Contrasts	
Measure:   MEASURE_1  	
Source	pairedtone	Noncent. Parameter	Observed Powera	
pairedtone	Linear	9.230	.846	
pairedtone * finalgroups	Linear	3.574	.458	
Error(pairedtone)	Linear			

a. Computed using alpha = .05	

Levene's Test of Equality of Error Variancesa	
	F	df1	df2	Sig.	
beep1_maxampl_lefttemporal	1.675	1	51	.201	
beep2_maxampl_lefttemporal	.729	1	51	.397	

Tests the null hypothesis that the error variance of the dependent variable is equal across groups.a	
a. Design: Intercept + finalgroups 
 Within Subjects Design: pairedtone	

Tests of Between-Subjects Effects	
Measure:   MEASURE_1  	
Transformed Variable:   Average  	
Source	Type III Sum of Squares	df	Mean Square	F	Sig.	Partial Eta Squared	Noncent. Parameter	
Intercept	1370.828	1	1370.828	220.491	.000	.812	220.491	
finalgroups	1.518	1	1.518	.244	.623	.005	.244	
Error	317.075	51	6.217					

Tests of Between-Subjects Effects	
Measure:   MEASURE_1  	
Transformed Variable:   Average  	
Source	Observed Powera	
Intercept	1.000	
finalgroups	.077	
Error		

a. Computed using alpha = .05	

Estimated Marginal Means

1. Grand Mean	
Measure:   MEASURE_1  	
Mean	Std. Error	95% Confidence Interval	
		Lower Bound	Upper Bound	
3.628	.244	3.137	4.118	

2. finalgroups

Estimates	
Measure:   MEASURE_1  	
finalgroups	Mean	Std. Error	95% Confidence Interval	
			Lower Bound	Upper Bound	
szt	3.749	.368	3.011	4.487	
control	3.507	.322	2.861	4.153	

Pairwise Comparisons	
Measure:   MEASURE_1  	
(I) finalgroups	(J) finalgroups	Mean Difference (I-J)	Std. Error	Sig.a	95% Confidence Interval for Differencea	
					Lower Bound	Upper Bound	
szt	control	.241	.489	.623	-.740	1.222	
control	szt	-.241	.489	.623	-1.222	.740	

Based on estimated marginal means	
a. Adjustment for multiple comparisons: Least Significant Difference (equivalent to no adjustments).	

Univariate Tests	
Measure:   MEASURE_1  	
	Sum of Squares	df	Mean Square	F	Sig.	Partial Eta Squared	Noncent. Parameter	
Contrast	.759	1	.759	.244	.623	.005	.244	
Error	158.538	51	3.109					

Univariate Tests	
Measure:   MEASURE_1  	
	Observed Powera	
Contrast	.077	
Error		

The F tests the effect of finalgroups. This test is based on the linearly independent pairwise comparisons among the estimated marginal means.	
a. Computed using alpha = .05	

3. pairedtone

Estimates	
Measure:   MEASURE_1  	
pairedtone	Mean	Std. Error	95% Confidence Interval	
			Lower Bound	Upper Bound	
1	4.085	.241	3.601	4.569	
2	3.171	.326	2.516	3.826	

Pairwise Comparisons	
Measure:   MEASURE_1  	
(I) pairedtone	(J) pairedtone	Mean Difference (I-J)	Std. Error	Sig.b	95% Confidence Interval for Differenceb	
					Lower Bound	Upper Bound	
1	2	.914*	.301	.004	.310	1.518	
2	1	-.914*	.301	.004	-1.518	-.310	

Based on estimated marginal means	
*. The mean difference is significant at the .05 level.	
b. Adjustment for multiple comparisons: Least Significant Difference (equivalent to no adjustments).	

Multivariate Tests	
	Value	F	Hypothesis df	Error df	Sig.	Partial Eta Squared	Noncent. Parameter	
Pillai's trace	.153	9.230a	1.000	51.000	.004	.153	9.230	
Wilks' lambda	.847	9.230a	1.000	51.000	.004	.153	9.230	
Hotelling's trace	.181	9.230a	1.000	51.000	.004	.153	9.230	
Roy's largest root	.181	9.230a	1.000	51.000	.004	.153	9.230	

Multivariate Tests	
	Observed Powerb	
Pillai's trace	.846	
Wilks' lambda	.846	
Hotelling's trace	.846	
Roy's largest root	.846	

Each F tests the multivariate effect of pairedtone. These tests are based on the linearly independent pairwise comparisons among the estimated marginal means.	
a. Exact statistic	
b. Computed using alpha = .05	

4. finalgroups * pairedtone

Estimates	
Measure:   MEASURE_1  	
finalgroups	pairedtone	Mean	Std. Error	95% Confidence Interval	
				Lower Bound	Upper Bound	
szt	1	3.921	.363	3.193	4.649	
	2	3.576	.491	2.590	4.562	
control	1	4.249	.318	3.611	4.886	
	2	2.766	.430	1.902	3.629	

Pairwise Comparisons	
Measure:   MEASURE_1  	
pairedtone	(I) finalgroups	(J) finalgroups	Mean Difference (I-J)	Std. Error	Sig.a	95% Confidence Interval for Differencea	
						Lower Bound	Upper Bound	
1	szt	control	-.327	.482	.500	-1.295	.640	
	control	szt	.327	.482	.500	-.640	1.295	
2	szt	control	.810	.653	.220	-.500	2.121	
	control	szt	-.810	.653	.220	-2.121	.500	

Based on estimated marginal means	
a. Adjustment for multiple comparisons: Bonferroni.	

Univariate Tests	
Measure:   MEASURE_1  	
pairedtone	Sum of Squares	df	Mean Square	F	Sig.	Partial Eta Squared	Noncent. Parameter	
1	Contrast	1.395	1	1.395	.461	.500	.009	.461	
	Error	154.289	51	3.025					
2	Contrast	8.546	1	8.546	1.540	.220	.029	1.540	
	Error	283.000	51	5.549					

Univariate Tests	
Measure:   MEASURE_1  	
pairedtone	Observed Powera	
1	Contrast	.102	
	Error		
2	Contrast	.230	
	Error		

Each F tests the simple effects of finalgroups within each level combination of the other effects shown. These tests are based on the linearly independent pairwise comparisons among the estimated marginal means.	
a. Computed using alpha = .05	

5. finalgroups * pairedtone

Estimates	
Measure:   MEASURE_1  	
finalgroups	pairedtone	Mean	Std. Error	95% Confidence Interval	
				Lower Bound	Upper Bound	
szt	1	3.921	.363	3.193	4.649	
	2	3.576	.491	2.590	4.562	
control	1	4.249	.318	3.611	4.886	
	2	2.766	.430	1.902	3.629	

Pairwise Comparisons	
Measure:   MEASURE_1  	
finalgroups	(I) pairedtone	(J) pairedtone	Mean Difference (I-J)	Std. Error	Sig.b	95% Confidence Interval for Differenceb	
						Lower Bound	Upper Bound	
szt	1	2	.345	.453	.449	-.564	1.254	
	2	1	-.345	.453	.449	-1.254	.564	
control	1	2	1.483*	.396	.000	.687	2.279	
	2	1	-1.483*	.396	.000	-2.279	-.687	

Based on estimated marginal means	
*. The mean difference is significant at the .05 level.	
b. Adjustment for multiple comparisons: Bonferroni.	

Multivariate Tests	
finalgroups	Value	F	Hypothesis df	Error df	Sig.	Partial Eta Squared	Noncent. Parameter	
szt	Pillai's trace	.011	.582a	1.000	51.000	.449	.011	.582	
	Wilks' lambda	.989	.582a	1.000	51.000	.449	.011	.582	
	Hotelling's trace	.011	.582a	1.000	51.000	.449	.011	.582	
	Roy's largest root	.011	.582a	1.000	51.000	.449	.011	.582	
control	Pillai's trace	.215	13.993a	1.000	51.000	.000	.215	13.993	
	Wilks' lambda	.785	13.993a	1.000	51.000	.000	.215	13.993	
	Hotelling's trace	.274	13.993a	1.000	51.000	.000	.215	13.993	
	Roy's largest root	.274	13.993a	1.000	51.000	.000	.215	13.993	

Multivariate Tests	
finalgroups	Observed Powerb	
szt	Pillai's trace	.116	
	Wilks' lambda	.116	
	Hotelling's trace	.116	
	Roy's largest root	.116	
control	Pillai's trace	.956	
	Wilks' lambda	.956	
	Hotelling's trace	.956	
	Roy's largest root	.956	

Each F tests the multivariate simple effects of pairedtone within each level combination of the other effects shown. These tests are based on the linearly independent pairwise comparisons among the estimated marginal means.	
a. Exact statistic	
b. Computed using alpha = .05	
Profile Plots


General Linear Model -  A 2 (group) x 2 (paired-tone) RM ANOVA to explore the maximum amplitude INFANT P50 in the central region.

Within-Subjects Factors	
Measure:   MEASURE_1  	
PairedTone	Dependent Variable	
1	BEEP1_MAXAMP_CENTRAL	
2	BEEP2_MAXAMP_CENTRAL	

Between-Subjects Factors	
	Value Label	N	
FINALGROUPS	1.00	SZT	14	
	2.00	CONTROL	21	

Descriptive Statistics	
	FINALGROUPS	Mean	Std. Deviation	N	
BEEP1_MAXAMP_CENTRAL	SZT	5.5203	3.86024	14	
	CONTROL	5.5485	4.85131	21	
	Total	5.5372	4.42064	35	
BEEP2_MAXAMP_CENTRAL	SZT	3.6150	5.84864	14	
	CONTROL	2.8519	5.49569	21	
	Total	3.1571	5.56679	35	

Box's Test of Equality of Covariance Matricesa	
Box's M	.874	
F	.271	
df1	3	
df2	38506.192	
Sig.	.846	

Tests the null hypothesis that the observed covariance matrices of the dependent variables are equal across groups.a	
a. Design: Intercept + FINALGROUPS 
 Within Subjects Design: PairedTone	

Multivariate Testsa	
Effect	Value	F	Hypothesis df	Error df	Sig.	
PairedTone	Pillai's Trace	.101	3.717b	1.000	33.000	.063	
	Wilks' Lambda	.899	3.717b	1.000	33.000	.063	
	Hotelling's Trace	.113	3.717b	1.000	33.000	.063	
	Roy's Largest Root	.113	3.717b	1.000	33.000	.063	
PairedTone * FINALGROUPS	Pillai's Trace	.003	.110b	1.000	33.000	.742	
	Wilks' Lambda	.997	.110b	1.000	33.000	.742	
	Hotelling's Trace	.003	.110b	1.000	33.000	.742	
	Roy's Largest Root	.003	.110b	1.000	33.000	.742	

Multivariate Testsa	
Effect	Partial Eta Squared	Noncent. Parameter	Observed Powerc	
PairedTone	Pillai's Trace	.101	3.717	.465	
	Wilks' Lambda	.101	3.717	.465	
	Hotelling's Trace	.101	3.717	.465	
	Roy's Largest Root	.101	3.717	.465	
PairedTone * FINALGROUPS	Pillai's Trace	.003	.110	.062	
	Wilks' Lambda	.003	.110	.062	
	Hotelling's Trace	.003	.110	.062	
	Roy's Largest Root	.003	.110	.062	

a. Design: Intercept + FINALGROUPS 
 Within Subjects Design: PairedTone	
b. Exact statistic	
c. Computed using alpha = .05	

Mauchly's Test of Sphericitya	
Measure:   MEASURE_1  	
Within Subjects Effect	Mauchly's W	Approx. Chi-Square	df	Sig.	Epsilonb	
					Greenhouse-Geisser	Huynh-Feldt	
PairedTone	1.000	.000	0	.	1.000	1.000	

Mauchly's Test of Sphericitya	
Measure:   MEASURE_1  	
Within Subjects Effect	Epsilon	
	Lower-bound	
PairedTone	1.000	

Tests the null hypothesis that the error covariance matrix of the orthonormalized transformed dependent variables is proportional to an identity matrix.a	
a. Design: Intercept + FINALGROUPS 
 Within Subjects Design: PairedTone	
b. May be used to adjust the degrees of freedom for the averaged tests of significance. Corrected tests are displayed in the Tests of Within-Subjects Effects table.	

Tests of Within-Subjects Effects	
Measure:   MEASURE_1  	
Source	Type III Sum of Squares	df	Mean Square	F	Sig.	
PairedTone	Sphericity Assumed	88.947	1	88.947	3.717	.063	
	Greenhouse-Geisser	88.947	1.000	88.947	3.717	.063	
	Huynh-Feldt	88.947	1.000	88.947	3.717	.063	
	Lower-bound	88.947	1.000	88.947	3.717	.063	
PairedTone * FINALGROUPS	Sphericity Assumed	2.629	1	2.629	.110	.742	
	Greenhouse-Geisser	2.629	1.000	2.629	.110	.742	
	Huynh-Feldt	2.629	1.000	2.629	.110	.742	
	Lower-bound	2.629	1.000	2.629	.110	.742	
Error(PairedTone)	Sphericity Assumed	789.785	33	23.933			
	Greenhouse-Geisser	789.785	33.000	23.933			
	Huynh-Feldt	789.785	33.000	23.933			
	Lower-bound	789.785	33.000	23.933			

Tests of Within-Subjects Effects	
Measure:   MEASURE_1  	
Source	Partial Eta Squared	Noncent. Parameter	Observed Powera	
PairedTone	Sphericity Assumed	.101	3.717	.465	
	Greenhouse-Geisser	.101	3.717	.465	
	Huynh-Feldt	.101	3.717	.465	
	Lower-bound	.101	3.717	.465	
PairedTone * FINALGROUPS	Sphericity Assumed	.003	.110	.062	
	Greenhouse-Geisser	.003	.110	.062	
	Huynh-Feldt	.003	.110	.062	
	Lower-bound	.003	.110	.062	
Error(PairedTone)	Sphericity Assumed				
	Greenhouse-Geisser				
	Huynh-Feldt				
	Lower-bound				

a. Computed using alpha = .05	

Tests of Within-Subjects Contrasts	
Measure:   MEASURE_1  	
Source	PairedTone	Type III Sum of Squares	df	Mean Square	F	Sig.	Partial Eta Squared	
PairedTone	Linear	88.947	1	88.947	3.717	.063	.101	
PairedTone * FINALGROUPS	Linear	2.629	1	2.629	.110	.742	.003	
Error(PairedTone)	Linear	789.785	33	23.933				

Tests of Within-Subjects Contrasts	
Measure:   MEASURE_1  	
Source	PairedTone	Noncent. Parameter	Observed Powera	
PairedTone	Linear	3.717	.465	
PairedTone * FINALGROUPS	Linear	.110	.062	
Error(PairedTone)	Linear			

a. Computed using alpha = .05	

Levene's Test of Equality of Error Variancesa	
	F	df1	df2	Sig.	
BEEP1_MAXAMP_CENTRAL	.767	1	33	.387	
BEEP2_MAXAMP_CENTRAL	.032	1	33	.860	

Tests the null hypothesis that the error variance of the dependent variable is equal across groups.a	
a. Design: Intercept + FINALGROUPS 
 Within Subjects Design: PairedTone	

Tests of Between-Subjects Effects	
Measure:   MEASURE_1  	
Transformed Variable:   Average  	
Source	Type III Sum of Squares	df	Mean Square	F	Sig.	Partial Eta Squared	Noncent. Parameter	
Intercept	1291.503	1	1291.503	46.156	.000	.583	46.156	
FINALGROUPS	2.268	1	2.268	.081	.778	.002	.081	
Error	923.377	33	27.981					

Tests of Between-Subjects Effects	
Measure:   MEASURE_1  	
Transformed Variable:   Average  	
Source	Observed Powera	
Intercept	1.000	
FINALGROUPS	.059	
Error		

a. Computed using alpha = .05	

Estimated Marginal Means

1. Grand Mean	
Measure:   MEASURE_1  	
Mean	Std. Error	95% Confidence Interval	
		Lower Bound	Upper Bound	
4.384	.645	3.071	5.697	

2. FINALGROUPS
Estimates	
Measure:   MEASURE_1  	
FINALGROUPS	Mean	Std. Error	95% Confidence Interval	
			Lower Bound	Upper Bound	
SZT	4.568	1.000	2.534	6.601	
CONTROL	4.200	.816	2.540	5.861	

Pairwise Comparisons	
Measure:   MEASURE_1  	
(I) FINALGROUPS	(J) FINALGROUPS	Mean Difference (I-J)	Std. Error	Sig.a	95% Confidence Interval for Differencea	
					Lower Bound	Upper Bound	
SZT	CONTROL	.367	1.291	.778	-2.258	2.993	
CONTROL	SZT	-.367	1.291	.778	-2.993	2.258	

Based on estimated marginal means	
a. Adjustment for multiple comparisons: Least Significant Difference (equivalent to no adjustments).	

Univariate Tests	
Measure:   MEASURE_1  	
	Sum of Squares	df	Mean Square	F	Sig.	Partial Eta Squared	Noncent. Parameter	
Contrast	1.134	1	1.134	.081	.778	.002	.081	
Error	461.689	33	13.991					

Univariate Tests	
Measure:   MEASURE_1  	
	Observed Powera	
Contrast	.059	
Error		

The F tests the effect of FINALGROUPS. This test is based on the linearly independent pairwise comparisons among the estimated marginal means.	
a. Computed using alpha = .05	

3. PairedTone
Estimates	
Measure:   MEASURE_1  	
PairedTone	Mean	Std. Error	95% Confidence Interval	
			Lower Bound	Upper Bound	
1	5.534	.774	3.959	7.109	
2	3.233	.973	1.255	5.212	

Pairwise Comparisons	
Measure:   MEASURE_1  	
(I) PairedTone	(J) PairedTone	Mean Difference (I-J)	Std. Error	Sig.a	95% Confidence Interval for Differencea	
					Lower Bound	Upper Bound	
1	2	2.301	1.194	.063	-.127	4.729	
2	1	-2.301	1.194	.063	-4.729	.127	

Based on estimated marginal means	
a. Adjustment for multiple comparisons: Least Significant Difference (equivalent to no adjustments).	

Multivariate Tests	
	Value	F	Hypothesis df	Error df	Sig.	Partial Eta Squared	Noncent. Parameter	
Pillai's trace	.101	3.717a	1.000	33.000	.063	.101	3.717	
Wilks' lambda	.899	3.717a	1.000	33.000	.063	.101	3.717	
Hotelling's trace	.113	3.717a	1.000	33.000	.063	.101	3.717	
Roy's largest root	.113	3.717a	1.000	33.000	.063	.101	3.717	

Multivariate Tests	
	Observed Powerb	
Pillai's trace	.465	
Wilks' lambda	.465	
Hotelling's trace	.465	
Roy's largest root	.465	

Each F tests the multivariate effect of PairedTone. These tests are based on the linearly independent pairwise comparisons among the estimated marginal means.	
a. Exact statistic	
b. Computed using alpha = .05	


4. FINALGROUPS * PairedTone

Estimates	
Measure:   MEASURE_1  	
FINALGROUPS	PairedTone	Mean	Std. Error	95% Confidence Interval	
				Lower Bound	Upper Bound	
SZT	1	5.520	1.199	3.080	7.960	
	2	3.615	1.507	.550	6.680	
CONTROL	1	5.548	.979	3.556	7.541	
	2	2.852	1.230	.349	5.355	

Pairwise Comparisons	
Measure:   MEASURE_1  	
PairedTone	(I) FINALGROUPS	(J) FINALGROUPS	Mean Difference (I-J)	Std. Error	Sig.a	95% Confidence Interval for Differencea	
						Lower Bound	
1	SZT	CONTROL	-.028	1.548	.986	-3.178	
	CONTROL	SZT	.028	1.548	.986	-3.122	
2	SZT	CONTROL	.763	1.945	.697	-3.194	
	CONTROL	SZT	-.763	1.945	.697	-4.720	

Pairwise Comparisons	
Measure:   MEASURE_1  	
PairedTone	(I) FINALGROUPS	(J) FINALGROUPS	95% Confidence Interval for Difference	
			Upper Bound	
1	SZT	CONTROL	3.122	
	CONTROL	SZT	3.178	
2	SZT	CONTROL	4.720	
	CONTROL	SZT	3.194	

Based on estimated marginal means	
a. Adjustment for multiple comparisons: Bonferroni.	

Univariate Tests	
Measure:   MEASURE_1  	
PairedTone	Sum of Squares	df	Mean Square	F	Sig.	Partial Eta Squared	Noncent. Parameter	
1	Contrast	.007	1	.007	.000	.986	.000	.000	
	Error	664.423	33	20.134					
2	Contrast	4.891	1	4.891	.154	.697	.005	.154	
	Error	1048.739	33	31.780					

Univariate Tests	
Measure:   MEASURE_1  	
PairedTone	Observed Powera	
1	Contrast	.050	
	Error		
2	Contrast	.067	
	Error		

Each F tests the simple effects of FINALGROUPS within each level combination of the other effects shown. These tests are based on the linearly independent pairwise comparisons among the estimated marginal means.	
a. Computed using alpha = .05	

5. FINALGROUPS * PairedTone
Estimates	
Measure:   MEASURE_1  	
FINALGROUPS	PairedTone	Mean	Std. Error	95% Confidence Interval	
				Lower Bound	Upper Bound	
SZT	1	5.520	1.199	3.080	7.960	
	2	3.615	1.507	.550	6.680	
CONTROL	1	5.548	.979	3.556	7.541	
	2	2.852	1.230	.349	5.355	

Pairwise Comparisons	
Measure:   MEASURE_1  	
FINALGROUPS	(I) PairedTone	(J) PairedTone	Mean Difference (I-J)	Std. Error	Sig.a	95% Confidence Interval for Differencea	
						Lower Bound	Upper Bound	
SZT	1	2	1.905	1.849	.310	-1.857	5.667	
	2	1	-1.905	1.849	.310	-5.667	1.857	
CONTROL	1	2	2.697	1.510	.083	-.375	5.768	
	2	1	-2.697	1.510	.083	-5.768	.375	

Based on estimated marginal means	
a. Adjustment for multiple comparisons: Bonferroni.	

Multivariate Tests	
FINALGROUPS	Value	F	Hypothesis df	Error df	Sig.	Partial Eta Squared	
SZT	Pillai's trace	.031	1.062a	1.000	33.000	.310	.031	
	Wilks' lambda	.969	1.062a	1.000	33.000	.310	.031	
	Hotelling's trace	.032	1.062a	1.000	33.000	.310	.031	
	Roy's largest root	.032	1.062a	1.000	33.000	.310	.031	
CONTROL	Pillai's trace	.088	3.190a	1.000	33.000	.083	.088	
	Wilks' lambda	.912	3.190a	1.000	33.000	.083	.088	
	Hotelling's trace	.097	3.190a	1.000	33.000	.083	.088	
	Roy's largest root	.097	3.190a	1.000	33.000	.083	.088	

Multivariate Tests	
FINALGROUPS	Noncent. Parameter	Observed Powerb	
SZT	Pillai's trace	1.062	.170	
	Wilks' lambda	1.062	.170	
	Hotelling's trace	1.062	.170	
	Roy's largest root	1.062	.170	
CONTROL	Pillai's trace	3.190	.411	
	Wilks' lambda	3.190	.411	
	Hotelling's trace	3.190	.411	
	Roy's largest root	3.190	.411	

Each F tests the multivariate simple effects of PairedTone within each level combination of the other effects shown. These tests are based on the linearly independent pairwise comparisons among the estimated marginal means.	
a. Exact statistic	
b. Computed using alpha = .05	
Profile Plots


General Linear Model -  A 2 (group) x 2 (paired-tone) RM ANOVA to explore the mean amplitude INFANT P50 in the central region.
  
Within-Subjects Factors	
Measure:   MEASURE_1  	
PairedTone	Dependent Variable	
1	BEEP1_MEANAMP_CENTRAL	
2	BEEP2_MEANAMP_CENTRAL	

Between-Subjects Factors	
	Value Label	N	
FINALGROUPS	1.00	SZT	14	
	2.00	CONTROL	21	

Descriptive Statistics	
	FINALGROUPS	Mean	Std. Deviation	N	
BEEP1_MEANAMP_CENTRAL	SZT	2.2991	3.66372	14	
	CONTROL	2.1740	4.42946	21	
	Total	2.2240	4.08379	35	
BEEP2_MEANAMP_CENTRAL	SZT	1.2822	6.18101	14	
	CONTROL	1.0132	5.58720	21	
	Total	1.1208	5.74355	35	

Box's Test of Equality of Covariance Matricesa	
Box's M	.714	
F	.221	
df1	3	
df2	38506.192	
Sig.	.882	

Tests the null hypothesis that the observed covariance matrices of the dependent variables are equal across groups.a	
a. Design: Intercept + FINALGROUPS 
 Within Subjects Design: PairedTone	

Multivariate Testsa	
Effect	Value	F	Hypothesis df	Error df	Sig.	
PairedTone	Pillai's Trace	.023	.772b	1.000	33.000	.386	
	Wilks' Lambda	.977	.772b	1.000	33.000	.386	
	Hotelling's Trace	.023	.772b	1.000	33.000	.386	
	Roy's Largest Root	.023	.772b	1.000	33.000	.386	
PairedTone * FINALGROUPS	Pillai's Trace	.000	.003b	1.000	33.000	.954	
	Wilks' Lambda	1.000	.003b	1.000	33.000	.954	
	Hotelling's Trace	.000	.003b	1.000	33.000	.954	
	Roy's Largest Root	.000	.003b	1.000	33.000	.954	

Multivariate Testsa	
Effect	Partial Eta Squared	Noncent. Parameter	Observed Powerc	
PairedTone	Pillai's Trace	.023	.772	.137	
	Wilks' Lambda	.023	.772	.137	
	Hotelling's Trace	.023	.772	.137	
	Roy's Largest Root	.023	.772	.137	
PairedTone * FINALGROUPS	Pillai's Trace	.000	.003	.050	
	Wilks' Lambda	.000	.003	.050	
	Hotelling's Trace	.000	.003	.050	
	Roy's Largest Root	.000	.003	.050	

a. Design: Intercept + FINALGROUPS 
 Within Subjects Design: PairedTone	
b. Exact statistic	
c. Computed using alpha = .05	

Mauchly's Test of Sphericitya	
Measure:   MEASURE_1  	
Within Subjects Effect	Mauchly's W	Approx. Chi-Square	df	Sig.	Epsilonb	
					Greenhouse-Geisser	Huynh-Feldt	
PairedTone	1.000	.000	0	.	1.000	1.000	

Mauchly's Test of Sphericitya	
Measure:   MEASURE_1  	
Within Subjects Effect	Epsilon	
	Lower-bound	
PairedTone	1.000	

Tests the null hypothesis that the error covariance matrix of the orthonormalized transformed dependent variables is proportional to an identity matrix.a	
a. Design: Intercept + FINALGROUPS 
 Within Subjects Design: PairedTone	
b. May be used to adjust the degrees of freedom for the averaged tests of significance. Corrected tests are displayed in the Tests of Within-Subjects Effects table.	

Tests of Within-Subjects Effects	
Measure:   MEASURE_1  	
Source	Type III Sum of Squares	df	Mean Square	F	Sig.	
PairedTone	Sphericity Assumed	19.917	1	19.917	.772	.386	
	Greenhouse-Geisser	19.917	1.000	19.917	.772	.386	
	Huynh-Feldt	19.917	1.000	19.917	.772	.386	
	Lower-bound	19.917	1.000	19.917	.772	.386	
PairedTone * FINALGROUPS	Sphericity Assumed	.087	1	.087	.003	.954	
	Greenhouse-Geisser	.087	1.000	.087	.003	.954	
	Huynh-Feldt	.087	1.000	.087	.003	.954	
	Lower-bound	.087	1.000	.087	.003	.954	
Error(PairedTone)	Sphericity Assumed	851.118	33	25.791			
	Greenhouse-Geisser	851.118	33.000	25.791			
	Huynh-Feldt	851.118	33.000	25.791			
	Lower-bound	851.118	33.000	25.791			

Tests of Within-Subjects Effects	
Measure:   MEASURE_1  	
Source	Partial Eta Squared	Noncent. Parameter	Observed Powera	
PairedTone	Sphericity Assumed	.023	.772	.137	
	Greenhouse-Geisser	.023	.772	.137	
	Huynh-Feldt	.023	.772	.137	
	Lower-bound	.023	.772	.137	
PairedTone * FINALGROUPS	Sphericity Assumed	.000	.003	.050	
	Greenhouse-Geisser	.000	.003	.050	
	Huynh-Feldt	.000	.003	.050	
	Lower-bound	.000	.003	.050	
Error(PairedTone)	Sphericity Assumed				
	Greenhouse-Geisser				
	Huynh-Feldt				
	Lower-bound				

a. Computed using alpha = .05	

Tests of Within-Subjects Contrasts	
Measure:   MEASURE_1  	
Source	PairedTone	Type III Sum of Squares	df	Mean Square	F	Sig.	Partial Eta Squared	
PairedTone	Linear	19.917	1	19.917	.772	.386	.023	
PairedTone * FINALGROUPS	Linear	.087	1	.087	.003	.954	.000	
Error(PairedTone)	Linear	851.118	33	25.791				

Tests of Within-Subjects Contrasts	
Measure:   MEASURE_1  	
Source	PairedTone	Noncent. Parameter	Observed Powera	
PairedTone	Linear	.772	.137	
PairedTone * FINALGROUPS	Linear	.003	.050	
Error(PairedTone)	Linear			

a. Computed using alpha = .05	

Levene's Test of Equality of Error Variancesa	
	F	df1	df2	Sig.	
BEEP1_MEANAMP_CENTRAL	.691	1	33	.412	
BEEP2_MEANAMP_CENTRAL	.335	1	33	.567	

Tests the null hypothesis that the error variance of the dependent variable is equal across groups.a	
a. Design: Intercept + FINALGROUPS 
 Within Subjects Design: PairedTone	

Tests of Between-Subjects Effects	
Measure:   MEASURE_1  	
Transformed Variable:   Average  	
Source	Type III Sum of Squares	df	Mean Square	F	Sig.	Partial Eta Squared	Noncent. Parameter	
Intercept	192.417	1	192.417	7.588	.009	.187	7.588	
FINALGROUPS	.652	1	.652	.026	.874	.001	.026	
Error	836.779	33	25.357					

Tests of Between-Subjects Effects	
Measure:   MEASURE_1  	
Transformed Variable:   Average  	
Source	Observed Powera	
Intercept	.762	
FINALGROUPS	.053	
Error		

a. Computed using alpha = .05	

Estimated Marginal Means
1. Grand Mean	
Measure:   MEASURE_1  	
Mean	Std. Error	95% Confidence Interval	
		Lower Bound	Upper Bound	
1.692	.614	.442	2.942	

2. FINALGROUPS
Estimates	
Measure:   MEASURE_1  	
FINALGROUPS	Mean	Std. Error	95% Confidence Interval	
			Lower Bound	Upper Bound	
SZT	1.791	.952	-.145	3.727	
CONTROL	1.594	.777	.013	3.174	

Pairwise Comparisons	
Measure:   MEASURE_1  	
(I) FINALGROUPS	(J) FINALGROUPS	Mean Difference (I-J)	Std. Error	Sig.a	95% Confidence Interval for Differencea	
					Lower Bound	Upper Bound	
SZT	CONTROL	.197	1.229	.874	-2.302	2.697	
CONTROL	SZT	-.197	1.229	.874	-2.697	2.302	

Based on estimated marginal means	
a. Adjustment for multiple comparisons: Least Significant Difference (equivalent to no adjustments).	

Univariate Tests	
Measure:   MEASURE_1  	
	Sum of Squares	df	Mean Square	F	Sig.	Partial Eta Squared	Noncent. Parameter	
Contrast	.326	1	.326	.026	.874	.001	.026	
Error	418.389	33	12.678					

Univariate Tests	
Measure:   MEASURE_1  	
	Observed Powera	
Contrast	.053	
Error		

The F tests the effect of FINALGROUPS. This test is based on the linearly independent pairwise comparisons among the estimated marginal means.	
a. Computed using alpha = .05	

3. PairedTone
Estimates	
Measure:   MEASURE_1  	
PairedTone	Mean	Std. Error	95% Confidence Interval	
			Lower Bound	Upper Bound	
1	2.237	.715	.782	3.691	
2	1.148	1.005	-.898	3.193	

Pairwise Comparisons	
Measure:   MEASURE_1  	
(I) PairedTone	(J) PairedTone	Mean Difference (I-J)	Std. Error	Sig.a	95% Confidence Interval for Differencea	
					Lower Bound	Upper Bound	
1	2	1.089	1.239	.386	-1.432	3.610	
2	1	-1.089	1.239	.386	-3.610	1.432	

Based on estimated marginal means	
a. Adjustment for multiple comparisons: Least Significant Difference (equivalent to no adjustments).	

Multivariate Tests	
	Value	F	Hypothesis df	Error df	Sig.	Partial Eta Squared	Noncent. Parameter	
Pillai's trace	.023	.772a	1.000	33.000	.386	.023	.772	
Wilks' lambda	.977	.772a	1.000	33.000	.386	.023	.772	
Hotelling's trace	.023	.772a	1.000	33.000	.386	.023	.772	
Roy's largest root	.023	.772a	1.000	33.000	.386	.023	.772	

Multivariate Tests	
	Observed Powerb	
Pillai's trace	.137	
Wilks' lambda	.137	
Hotelling's trace	.137	
Roy's largest root	.137	

Each F tests the multivariate effect of PairedTone. These tests are based on the linearly independent pairwise comparisons among the estimated marginal means.	
a. Exact statistic	
b. Computed using alpha = .05	


4. FINALGROUPS * PairedTone
Estimates	
Measure:   MEASURE_1  	
FINALGROUPS	PairedTone	Mean	Std. Error	95% Confidence Interval	
				Lower Bound	Upper Bound	
SZT	1	2.299	1.108	.045	4.553	
	2	1.282	1.558	-1.887	4.451	
CONTROL	1	2.174	.904	.334	4.014	
	2	1.013	1.272	-1.574	3.601	

Pairwise Comparisons	
Measure:   MEASURE_1  	
PairedTone	(I) FINALGROUPS	(J) FINALGROUPS	Mean Difference (I-J)	Std. Error	Sig.a	95% Confidence Interval for Differencea	
						Lower Bound	
1	SZT	CONTROL	.125	1.430	.931	-2.784	
	CONTROL	SZT	-.125	1.430	.931	-3.035	
2	SZT	CONTROL	.269	2.011	.894	-3.822	
	CONTROL	SZT	-.269	2.011	.894	-4.360	

Pairwise Comparisons	
Measure:   MEASURE_1  	
PairedTone	(I) FINALGROUPS	(J) FINALGROUPS	95% Confidence Interval for Difference	
			Upper Bound	
1	SZT	CONTROL	3.035	
	CONTROL	SZT	2.784	
2	SZT	CONTROL	4.360	
	CONTROL	SZT	3.822	

Based on estimated marginal means	
a. Adjustment for multiple comparisons: Bonferroni.	

Univariate Tests	
Measure:   MEASURE_1  	
PairedTone	Sum of Squares	df	Mean Square	F	Sig.	Partial Eta Squared	Noncent. Parameter	
1	Contrast	.131	1	.131	.008	.931	.000	.008	
	Error	566.898	33	17.179					
2	Contrast	.608	1	.608	.018	.894	.001	.018	
	Error	1120.998	33	33.970					

Univariate Tests	
Measure:   MEASURE_1  	
PairedTone	Observed Powera	
1	Contrast	.051	
	Error		
2	Contrast	.052	
	Error		

Each F tests the simple effects of FINALGROUPS within each level combination of the other effects shown. These tests are based on the linearly independent pairwise comparisons among the estimated marginal means.	
a. Computed using alpha = .05	

5. FINALGROUPS * PairedTone
Estimates	
Measure:   MEASURE_1  	
FINALGROUPS	PairedTone	Mean	Std. Error	95% Confidence Interval	
				Lower Bound	Upper Bound	
SZT	1	2.299	1.108	.045	4.553	
	2	1.282	1.558	-1.887	4.451	
CONTROL	1	2.174	.904	.334	4.014	
	2	1.013	1.272	-1.574	3.601	

Pairwise Comparisons	
Measure:   MEASURE_1  	
FINALGROUPS	(I) PairedTone	(J) PairedTone	Mean Difference (I-J)	Std. Error	Sig.a	95% Confidence Interval for Differencea	
						Lower Bound	Upper Bound	
SZT	1	2	1.017	1.920	.600	-2.888	4.922	
	2	1	-1.017	1.920	.600	-4.922	2.888	
CONTROL	1	2	1.161	1.567	.464	-2.028	4.349	
	2	1	-1.161	1.567	.464	-4.349	2.028	

Based on estimated marginal means	
a. Adjustment for multiple comparisons: Bonferroni.	

Multivariate Tests	
FINALGROUPS	Value	F	Hypothesis df	Error df	Sig.	Partial Eta Squared	
SZT	Pillai's trace	.008	.281a	1.000	33.000	.600	.008	
	Wilks' lambda	.992	.281a	1.000	33.000	.600	.008	
	Hotelling's trace	.009	.281a	1.000	33.000	.600	.008	
	Roy's largest root	.009	.281a	1.000	33.000	.600	.008	
CONTROL	Pillai's trace	.016	.549a	1.000	33.000	.464	.016	
	Wilks' lambda	.984	.549a	1.000	33.000	.464	.016	
	Hotelling's trace	.017	.549a	1.000	33.000	.464	.016	
	Roy's largest root	.017	.549a	1.000	33.000	.464	.016	

Multivariate Tests	
FINALGROUPS	Noncent. Parameter	Observed Powerb	
SZT	Pillai's trace	.281	.081	
	Wilks' lambda	.281	.081	
	Hotelling's trace	.281	.081	
	Roy's largest root	.281	.081	
CONTROL	Pillai's trace	.549	.111	
	Wilks' lambda	.549	.111	
	Hotelling's trace	.549	.111	
	Roy's largest root	.549	.111	

Each F tests the multivariate simple effects of PairedTone within each level combination of the other effects shown. These tests are based on the linearly independent pairwise comparisons among the estimated marginal means.	
a. Exact statistic	
b. Computed using alpha = .05	
Profile Plots
